# Supplementary material for: A cost efficient spatially balanced hierarchical sampling design for monitoring boreal birds incorporating access costs and habitat stratification
Source: PLoS One. 2020 Jun 16;15(6):e0234494. doi: 10.1371/journal.pone.0234494 (PMC7297386; doi:10.1371/journal.pone.0234494)
Supplement: S5 Data — (ZIP) [file pone.0234494.s005.zip › .checkpoint/R-3.5.3/compiler/html/00Index.html]

R: The R Compiler Package

# The R Compiler Package

---

## Documentation for package ‘compiler’ version 3.5.3

- DESCRIPTION file.

## Help Pages

|  |  |
| --- | --- |
| cmpfile | Byte Code Compiler |
| cmpfun | Byte Code Compiler |
| compile | Byte Code Compiler |
| compilePKGS | Byte Code Compiler |
| disassemble | Byte Code Compiler |
| enableJIT | Byte Code Compiler |
| getCompilerOption | Byte Code Compiler |
| loadcmp | Byte Code Compiler |
| setCompilerOptions | Byte Code Compiler |
