# Supplementary material for: A cost efficient spatially balanced hierarchical sampling design for monitoring boreal birds incorporating access costs and habitat stratification
Source: PLoS One. 2020 Jun 16;15(6):e0234494. doi: 10.1371/journal.pone.0234494 (PMC7297386; doi:10.1371/journal.pone.0234494)
Supplement: S5 Data — (ZIP) [file pone.0234494.s005.zip › .checkpoint/R-3.5.3/compiler/html/compile.html]

R: Byte Code Compiler

|  |  |
| --- | --- |
| compile {compiler} | R Documentation |

## Byte Code Compiler

### Description

These functions provide an interface to a byte code compiler for **R**.

### Usage

```
cmpfun(f, options = NULL)
compile(e, env = .GlobalEnv, options = NULL, srcref = NULL)
cmpfile(infile, outfile, ascii = FALSE, env = .GlobalEnv,
        verbose = FALSE, options = NULL)
loadcmp(file, envir = .GlobalEnv, chdir = FALSE)
disassemble(code)
enableJIT(level)
compilePKGS(enable)
getCompilerOption(name, options)
setCompilerOptions(...)
```

### Arguments

|  |  |
| --- | --- |
| `f` | a closure. |
| `options` | list of named compiler options: see ‘Details’. |
| `env` | the top level environment for the compiling. |
| `srcref` | initial source reference for the expression. |
| `file,infile,outfile` | pathnames; outfile defaults to infile with a ‘.Rc’ extension in place of any existing extension. |
| `ascii` | logical; should the compiled file be saved in ascii format? |
| `verbose` | logical; should the compiler show what is being compiled? |
| `envir` | environment to evaluate loaded expressions in. |
| `chdir` | logical; change directory before evaluation? |
| `code` | byte code expression or compiled closure |
| `e` | expression to compile. |
| `level` | integer; the JIT level to use (`0` to `3`). |
| `enable` | logical; enable compiling packages if `TRUE`. |
| `name` | character string; name of option to return. |
| `...` | named compiler options to set. |

### Details

The function `cmpfun` compiles the body of a closure and
returns a new closure with the same formals and the body replaced by
the compiled body expression.

`compile` compiles an expression into a byte code object; the
object can then be evaluated with `eval`.

`cmpfile` parses the expressions in `infile`, compiles
them, and writes the compiled expressions to `outfile`. If
`outfile` is not provided, it is formed from `infile` by
replacing or appending a `.Rc` suffix.

`loadcmp` is used to load compiled files. It is similar to
`sys.source`, except that its default loading environment is the
global environment rather than the base environment.

`disassemble` produces a printed representation of the code
that may be useful to give a hint of what is going on.

`enableJIT` enables or disables just-in-time (JIT)
compilation. JIT is disabled if the argument is 0. If `level` is
1 then larger closures are compiled before their first use. If
`level` is 2, then some small closures are also compiled before
their second use. If `level` is 3 then in addition
all top level loops are compiled before they are executed. JIT level
3 requires the compiler option `optimize` to be 2 or 3. The JIT
level can also be selected by starting **R** with the environment
variable `R_ENABLE_JIT` set to one of these values. Calling
`enableJIT` with a negative argument returns the current JIT
level. The default JIT level is `3`.

`compilePKGS` enables or disables compiling packages when they
are installed. This requires that the package uses lazy loading as
compilation occurs as functions are written to the lazy loading data
base. This can also be enabled by starting **R** with the environment
variable `_R_COMPILE_PKGS_` set to a positive integer value.
This should not be enabled outside package installation, because it
causes any serialized function to be compiled, which comes with
time and space overhead. `R_COMPILE_PKGS` can be used, instead,
to instruct `INSTALL` to enable/disable compilation of packages
during installation.

Currently the compiler warns about a variety of things. It does
this by using `cat` to print messages. Eventually this should
use the condition handling mechanism.

The `options` argument can be used to control compiler operation.
There are currently four options: `optimize`, `suppressAll`,
`suppressUndefined`, and `suppressNoSuperAssignVar`.
`optimize` specifies the optimization level, an integer from `0`
to `3` (the current out-of-the-box default is `2`).
`suppressAll` should be a scalar logical; if `TRUE` no messages
will be shown (this is the default). `suppressUndefined` can be
`TRUE` to suppress all messages about undefined variables, or it can
be a character vector of the names of variables for which messages should
not be shown. `suppressNoSuperAssignVar` can be `TRUE` to
suppress messages about super assignments to a variable for which no
binding is visible at compile time. During compilation of packages,
`suppressAll` is currently `FALSE`, `suppressUndefined` is
`TRUE` and `suppressNoSuperAssignVar` is `TRUE`.

`getCompilerOption` returns the value of the specified option.
The default value is returned unless a value is supplied in the
`options` argument; the `options` argument is primarily for
internal use. `setCompilerOption` sets the default option
values. Options to set are identified by argument names, e.g.
`setCompilerOptions(suppressAll = TRUE, optimize = 3)`.
It returns a named list of the previous values.

Calling the compiler a byte code compiler is actually a bit of a
misnomer: the external representation of code objects currently uses
`int` operands, and when compiled with `gcc` the internal
representation is actually threaded code rather than byte code.

### Author(s)

Luke Tierney

### Examples

```
oldJIT <- enableJIT(0)
# a simple example
f <- function(x) x+1
fc <- cmpfun(f)
fc(2)
disassemble(fc)

# old R version of lapply
la1 <- function(X, FUN, ...) {
    FUN <- match.fun(FUN)
    if (!is.list(X))
	X <- as.list(X)
    rval <- vector("list", length(X))
    for(i in seq(along = X))
	rval[i] <- list(FUN(X[[i]], ...))
    names(rval) <- names(X)		  # keep `names' !
    return(rval)
}
# a small variation
la2 <- function(X, FUN, ...) {
    FUN <- match.fun(FUN)
    if (!is.list(X))
	X <- as.list(X)
    rval <- vector("list", length(X))
    for(i in seq(along = X)) {
        v <- FUN(X[[i]], ...)
        if (is.null(v)) rval[i] <- list(v)
        else rval[[i]] <- v
    }
    names(rval) <- names(X)		  # keep `names' !
    return(rval)
}
# Compiled versions
la1c <- cmpfun(la1)
la2c <- cmpfun(la2)
# some timings
x <- 1:10
y <- 1:100

system.time(for (i in 1:10000) lapply(x, is.null))
system.time(for (i in 1:10000) la1(x, is.null))
system.time(for (i in 1:10000) la1c(x, is.null))
system.time(for (i in 1:10000) la2(x, is.null))
system.time(for (i in 1:10000) la2c(x, is.null))
system.time(for (i in 1:1000) lapply(y, is.null))
system.time(for (i in 1:1000) la1(y, is.null))
system.time(for (i in 1:1000) la1c(y, is.null))
system.time(for (i in 1:1000) la2(y, is.null))
system.time(for (i in 1:1000) la2c(y, is.null))


enableJIT(oldJIT)
```

---

[Package *compiler* version 3.5.3 Index]
