# Supplementary material for: A cost efficient spatially balanced hierarchical sampling design for monitoring boreal birds incorporating access costs and habitat stratification
Source: PLoS One. 2020 Jun 16;15(6):e0234494. doi: 10.1371/journal.pone.0234494 (PMC7297386; doi:10.1371/journal.pone.0234494)
Supplement: S7 Data — (ZIP) [file pone.0234494.s007.zip › Supporting Information S7_Geoprocessing and Cost Model details for Manitoba.docx]

**Supporting Information S7: Geoprocessing and Cost Model details for Manitoba.**

Zhong Li^1^, Kyle Swiston^1^, Caitin Mader^1^

^1^Environment and Climate Change Canada, Edmonton, AB, Canada

# **Geoprocessing methods:**

## **1. Road access**

**Overview:** The most cost effective method for accessing sites. Assumes crews can access PSUs that intersect with road networks via truck, and walk the remaining 0-5 km to point count locations.

**Base data:**

### 1.1 Partial Manitoba road data 1 [1]:

Linear Referencing System (LRS) road network (2018), obtained from the Manitoba Land use initiative, 2018.

<http://mli2.gov.mb.ca/roads_hwys/shp_zip_files/trn_lrs_highway_network_2018_shp.zip>

### 1.2 Partial Manitoba road data 2 [2]:

Manitoba-National Road Network, obtained from the Manitoba Land use initiative, 2018.

<http://mli2.gov.mb.ca/roads_hwys/shp_zip_files/nrn_rrn_mb_shp_en.zip>

### 1.3 **Processing:**

1. The two datasets (1.1 & 1.2) were combined to create a complete map of local and regional roads.
2. The combined dataset was split by road class into roads that are navigable year round (1.4), and roads navigable only in winter (1.5).

**Final datasets:**

### 1.4 Final Year-round roads surface:

“Truck_cost” attribute in MB_brand_hexagons_2020 dataset

**Truck cost Assumptions**

Cost includes transportation and crew costs

Cost is driven primarily by distance to starting PC which translates to reduced productivity

Assume productivity after the first PC is the same

Walking speed = 2km/h

**Truck Cost application:**

Daily cost = $600 ($450 prof+camp + $150 vehicle)

Apply uniform cost to hexagons intersecting road network.

### 1.5 Final Winter roads surface:

“Win_road_c” attribute in MB_brand_hexagons_2020 dataset

**Winter road Assumptions**

Will need to stay in hotels etc with long travel distances (no camping).

**Winter road Cost application:**
Assume daily cost = ($600 + 200 (accom)) * 1.5 (assume getting half as many done per day due to cost of added travel.
Apply uniform cost to all hexagons intersecting winter roads.

## **2. Rail access**

**Overview:** Two passenger rail companies operate in boreal Manitoba- Keewatin Rail and Via rail. Keewatin Rail allows between station dropoff and pickup, while Via Rail does not.

**Base data:**

### 2.1 Northern Rail Line (stations and track) [3]:

Canvec_50k_MB_Transport_shp/track_segment_1.shp
Obtained from: <http://ftp.maps.canada.ca/pub/nrcan_rncan/vector/canvec/shp/Transport/>

### 2.2 Processing:

1. Clip to Manitoba border
 2. Separate track sections by rail operator

 **Final datasets:**

### **2.3 Final Keewatin Rail surface:**

“Keewatin” field in MB_brandt_hext_2020

**Keewatin rail assumptions**

- The Pas to Pukatawagan line runs twice a week.
- Can set up pick up and drop off at any point on route, even between stations.
- Assume 1 day travel time (1/2 day per way) back to major station/town. Apply the standard $450 camp cost to this day of travel.
- Fares are $40 per technician per way
- Pick ups and drop offs to between-station stops need to be set up with Keewatin rail in advance.

$$Cost= \frac{\$80 fare+\$50x2x2 bike transport+\$450 camp cost during travel+\$1350 camp costs at sites}{2 sites}=\$1040 per site$$

Cost application:

Apply uniform cost value to all hexagons intersecting track section

### **2.4 Final Via Rail surface:**

“ViaTra_cst” attribute in MB_brandt_hex_2020 dataset

**Via rail assumptions**

- Winnipeg to Churchill line runs three times a week.
- Can set up pick up and drop off at any one of 63 stations/sign posts on route
- Stations are typically located 15-25km apart. Model assumes that two independent sites could be sampled in the approximately 72 hour window between trail drop off and pick up. Daily camp costs will need to be applied 3 times.
- Crews will use mountain bikes to move between stations. Charge of $50 per oversized luggage piece per day.
- Assume 1 day travel time (1/2 day per way) back to major station/town. Apply the standard $450 camp cost to this day of travel.
- Fares vary from $25 to $250 per technician per way depending on how far you are travelling on the Winnipeg-Churchill line. Under the assumption that crews would not travel more than a third of the line from their starting point, fare was generalized to $100 per direction per technician ($200x2 = $400 round trip).
- Pick ups and drop offs to minor stops need to be set up with Via rail at least 2 weeks in advance.

Overall train model

$$Cost= \frac{\$400 fare+\$50x2x2 bike transport+\$450 camp cost during travel+\$1350 camp costs at sites}{2 sites}=\$1200 per site$$

Apply to hexagons within 200m of rail line

Cost application:

Apply uniform cost value to all hexagons intersecting track section

## **ATV access**

**Overview:** Identifies hexagons accessible through a combination of road access with a truck, and off-road access with ATVs. Assumes that if crews can access PSUs using ATV trails, they can walk any remaining distance (up to 5km) to point count locations.

**Base data:**

### 3.1 Trails layer [4]:

Canvec_50k_MB_Transport_shp/trail1.shp
Obtained from: <http://ftp.maps.canada.ca/pub/nrcan_rncan/vector/canvec/shp/Transport/>

### 3.2 Processing:

Trails layer was pruned to cover trails with road access, using a stepwise selection process of polylines intersecting with trails features branching from roads.

**Final dataset:**

### 3.3 Final ATV access surface:

“atv_cost” attribute in MB_brandt_hex_2020 dataset

**Assumptions**

Cost includes transportation and crew costs

Cost is driven primarily by distance to starting PC which translates to reduced productivity

Assume productivity after the first PC is the same

Walking speed = 2km/h

**Cost application**

Daily cost = $750 ($450 prof+camp + $150 ATV)

If snowmobile AND leaving ARUs that CANNOT be retrieved in summer, then double the cost for lost opportunity cost (Not included in this estimate).

Apply to hexagons intersecting trails.

## **Helicopter and Float plane access**

**Overview:**

**Base data:**

### 4.1 Locations of air bases servicing Manitoba [5]:

Compiled from list of contracts on air charter services website. <https://buyandsell.gc.ca/procurement-data/tender-notice/PW-ZL-102-25299/list-of-interested-suppliers>
Lat/Long verified from NCR Toporama - <http://atlas.nrcan.gc.ca/toporama/en/index.html>

**Processing:**

### 4.2 Manual creation of shapefile:

Use online data to create dataset containing locations, pricing and available aircraft at each listed air base.

### 4.3 Generate Euclidean distance rasters:

1. Calculate Euclidean distance for each float (and heliport) plane base, which was subsequently converted to distance in miles (e.g., mlswollaston). The distance datasets in miles were used to calculate the costs as air charter quotes are costs per mile.
2. Create two versions of the air base dataset, one which includes only float plane data, and one which includes only helicopter data. Many bases offer both, so there will be repeats.

### 4.4 Helicopter Cost model application:

All active heliports were modeled using the same generalized helicopter cost model. Minimum charge for MB helicopter charters is typically for 4 hours, at ~2250 per hour wet rate (fuel included). These figures were confirmed with larger charter companies operating in Manitoba. All helicopter data is based on A-star 350 models, which have a maximum range of ~150 km. Since flight time for this maximum range is less than the daily minimum, trip cost up to 150 km = $9000.

### 4.5 Final Helicopter access surface:

“Min_hel_co” attribute in MB_brandt_hex_2020

### 4.6 Addition of fuel caching at Shamattawa:

Access by helicopter to the Northwestern corner of Manitoba requires fuel caching, as it is out of range of a single trip. Custom helicopters’ fuel cache in Shamattawa offers some extension of this range, but additional fuel caching is necessary. Calculate these costs, and apply them to hexagons that fall within these ranges to create layer 4.7, Helicopter Access caching

### 4.7 Final Heli cache surface:

“Min_Sham_Y” field in MB_brandt_hex_2020

### 4.8 Application of floatplane flight cost models:

**Floatplane cost Layers –** Raster layers of Euclidean distance were calculated for each active waterdrome with access to a Turbo Otter or Twin Otter floatplane charter. A range of 600 km (1200 km round trip) was set for these layers. This was based on an estimate of the published maximum range of ~850 miles (1360km) for these planes. It is possible that a range of 500-550km is more probable for older planes. Because the Euclidean distance tool automatically sets the extent of rasters to the UTM Zone 14N for layers in that projection, the extent had to be manually set the boreal border outline layer.

Distance layers in miles were derived by dividing the distance layers in meters by 1609.34 in the raster calculator.

Cost layers were generated using the raster calculator, with distance layers in miles as the input, along with price information gathered from http://aircharter.pwgsc.gc.ca/index.cfm?fuseaction=catalogue.home&lang=e and from contacting individual charters via email or telephone. See below for the individual models. Fuel prices were estimated from airport info in each community

**Flin Flon – Wings over Kississing DHC3 Otter Turbine – Min cost 938.6, RPM (wet rate) = 13.41**

Con("MlsFFlon" <= 35, 2327.2, (900 + ("MlsFFlon" * 2 * 13.41)* 2)) ### assume Turbo Otter 2327.2 = 450 camp cost + 938.6*2, $13.41 per mile

**Lynn Lake – Transwest Air DHC3 Otter Turbine – Min cost 1440, RPM (dry) = 11.52, LPM = 2.19**

Con("MlsLynnL" <= 45, 3330, (900 + (("MlsLynnL" * 2 * 11.52) + ("MlsLynnL" * 2 * 2.19 * 1.9)) * 2)) ### assume Turbo Otter 3330 = 450 camp cost + 1440*2, 1.4 lpm, $1.90 /L, $11.52 per mile

**Pine Falls – Blue Water Aviation DHC3 Otter Turbine – Min cost 432, RPM (dry) = 9.1, LPM = 1.64**

Con("MlsPineF" <= 20, 1314, (900 + (("MlsPineF" * 2 * 9.10) + ("MlsPineF" * 2 * 1.4 * 1.80)) * 2)) ### assume Turbo Otter 1314 = 450 camp cost + 432*2, 1.4 lpm, $1.80 /L, $9.10 per mile

**Thompson – Wings over Kississing DHC3 Otter Turbine – Min cost 938.6, RPM (wet rate) = 13.41**

Con("MlsThomp" <= 35, 2327.2, (900 + ("MlsThomp" * 2 * 13.41)* 2)) ### assume Turbo Otter 2327.2 = 450 camp cost + 938.6*2, $13.41 per mile

**Wollaston Lake – Transwest Air DHC6 twin Otter – Min cost 1557, RPM (dry) = 12.11**

Con("MlsWollaston" <= 40, 3564, (900 + (("MlsWollaston" * 2 * 12.11) + ("MlsWollaston" * 2 * 3.44 * 2.29)) * 2)) ### assume Twin Otter, 3564 = 450 camp cost + 1557*2, 3.44 lpm, $2.29 /L, $12.11 per mile

Create a final float cost model:

Calculate the minimum of all the individual float plane cost models (assume people will work from the optimal aerodrome). There is a cost column in this dataset, and the value is the minimum of all the cost assumptions (Steve did some via interactive selection, e.g. selected all hexagons touching roads and applied road cost assumptions).

The cost models to examine are:

“minfltcost” = the minimum cost of all the individual float plane models (for different aerodromes)

“costbuffalo” = float plane cost model for Buffalo Narrows aerodrome

“costlaronge” = float plane cost model for Lac La Ronge aerodrome

“costotterlake” = float plane cost model for Otter Lake (Missinippe) aerodrome

“costpntsnorth” = float plane cost model for Points North Landing aerodrome

“coststony” = float plane cost model for Stony Rapids aerodrome

“costwollaston” = float plane cost model for Wollaston Lake aerodrome

Combine all cost models into single cost-distance raster (layer 4.9). Where cost-distances overlap, choose the lowest value.

### 4.9 Floatplane cost distances:

Intermediate dataset. Requires merging with float-accessible lakes layer.

### 4.10 Create layer of Floatplane accessible waterbody sections:

The goal is to create province-wide layer of places where a pilot could land a floatplane, given the specifications that a float plane needs a straight piece of water at least 250m wide and 3000m long to take off and land.

1. Start with “MB_connected_waterbody” layer (dataset 6.3)
2. Add a negative buffer of 126m. This eliminates sections of waterways less than 250m wide.
3. Convert buffered layer from multipart to singlepart.
4. From singlepart layer, select only polygons with a length of over 3000m, and convert this to its own layer
5. Buffer resulting layer by 200m. This is your new clip template.
6. Clip original “MB_connected_waterbody” layer to the clip template you just create. The clipped result will be the new “Float_accessible_waterways” layer

### 4.11 “Float_accessible_waterways” layer:

Intermediate layer representing sections (not whole waterbodies) of lakes and rivers where a floatplane could likely take off and land. Consultation with float plane pilot that the model did a reasonable job of identifying “probably landable” waterway sections. Individual assessment of landing sites by contracted pilots obviously still required.

### 4.12 Extract data from Floatplane cost distance raster (4.9):

Use to find minimum cost for float plane access for hexagon containing accessole waterbody, resulting in dataset 4.13

### 4.13 Final Floatplane access surface:

Float_new” field in MB_brandt_hex_2020

## **5. Waterbodies**

**Overview:**

A complete shapefile of all waterbodies in Manitoba was necessary. The waterbodies layer itself does not provide a cost model, but is used as base data for other cost models: The Floatplane, Canoe river trip, Lake Winnipeg powerboat, flatwater canoe and flatwater powerboat access models were all built using this waterbodies layer.

**Base data:**

### 5.1 Water (Lakes, Rivers and Streams) [6]: water_course1.shp, water_body2.shp in canvec_250K_MB_Hydro_shp.zip (tried 50k version but data set was 8.5GB and really slowed down processing) From: <http://ftp.maps.canada.ca/pub/nrcan_rncan/vector/canvec/shp/Hydro/>

**Processing:**

### 5.2 Merge water_course1.shp and water_body2.shp:

Dissolve to remove tile cuts and allow only single part features to make disconnected waterbodies individual features.

**Resulting dataset:**

### 5.3 “MB_connected_waterbody” dataset:

Intermediate dataset used for construction of Floatplane (4.13), Lake Winnipeg powerboat (6.5), Canoe river trip (7.5), flatwater canoe (8.6) and flatwater powerboat (8.7) access models.

### 5.4 Processing to cut waterbodies at probable rapids:

Flatwater access layer generation: Creates polygon layer that more realistically represents navigable connectivity between waterbodies by cutting polygons at locations likely to be rapids. Canoe access trips planned individually can expand from this map by adding portages, but for the purposes of generating a “starting point” map, we want to assume no portages. These steps separate water bodies at “pinch points” of 100m or less, which are likely to be rapids. This number is an estimate based on examining the Thlewiaza river and Nueltin Lake, to find a distance that would cut at river rapids but not unnecessarily segment lakes with small bottlenecks.

1. Dissolve MB float plane lakes layer to get rid of water bodies split by tiles
2. Inside buffer (-50 m) on waterbodies layer
3. Multipoint to singlepoint.
4. Manually cut out Hudson’s Bay and adjoining rivers, up to first navigable lake, as navigable rivers leading into the bay have already been included in the River Routes surface, and adjoining flatwater detours will be added separately.
5. Nelson River was manually removed, as hydro dams make its navigation laborious and dangerous.

**Resulting dataset:**

### 5.5 Accessible waterbodies dataset:

Polygon layer of all waterbodies in Manitoba, with polygons cut at “bottlenecks” that are not likely navigable by canoe or powerboat. Used as base data for river canoe route (7.5), and flatwater access (8.6, 8.7)

## Powerboat access on Lake Winnipeg

Overview: Identifies and estimates access costs for areas on the shores of Lake Winnipeg that could be reached by powerboat.

### 6.1 Lake Winnipeg boat launch data:

**Lake Winnipeg Boat Launches –** Road accessible public boat launches for Lake Winnipeg communities without marinas were gathered from various internet sources, and confirmed on Google Earth and NCR Toporama.

**Marinas -** Canvec_50k_MB_Transport_shp/marina_0.shp [7]

From: <http://ftp.maps.canada.ca/pub/nrcan_rncan/vector/canvec/shp/Transport/>

Mainly focused on Marinas around Lake Winnipeg, clipping those in the southern portion of the province

### **6.2 Processing:**

**Lake Winnipeg –** A single polygon for Lake Winnipeg was created by dissolving the Lake Winnipeg polygons in the water body layer. Island vertexes were manually deleted using the editor. The lake polygon was then converted to a polyline feature. The polyline was then simplified by using the simplify line tool in the cartography toobox. The Simplify line tool was set to the bend_simplify algorithm, at a tolerance of 1000 m. This eliminated some of the smaller shoreline features that would have artificially inflated travel distances by a boat following the coast of Lake Winnipeg.

**Lake Winnipeg shoreline routes –** 3 road accessible boat launches and 4 marinas were selected along the shoreline of Lake Winnipeg as cut points in the simplified shoreline polyline generated above. This generated 7 lines, representing shoreline boat routes between adjacent marinas/boat launches. There are additional marinas in the southern basin of Lake Winnipeg, but several are very close together (<10km). In these cases, a single marina was selected to represent the clustered group of ports. Shoreline hex layers were then generated by selecting boreal hexagons that were within 200m of route polylines. Centroid points for each of the shoreline hex layers were then generated using the features>features to point tool in the data management toolbox.

**Lake Winnipeg shoreline distances from Marinas/Boat Launches –** The Create Routes tool in the Linear Referencing toolbox was used to created measured polylines for the two routes starting at each marina or boat launch (typically a north route or a south route from the point feature). This generated a total of 14 routes, two directions for each of the line segments created above. The “Locate Features Along Route” tool in the same toolbox was then used to measure the distance of each hexagon centroid located along each route. A search radius of 5000 metres was used for this operation. The resulting distance measurements were then joined to the attribute tables of the shoreline hex layers.

### 6.3 Pricing model:

Powerboats to be rented from:

<http://www.parksiderentals.ca/>

- About 35km from the base of Lake Winnipeg, off of Red River.

PRICING DETAILS FROM PHONE:

18ft Lund with 60HP Mercury - oil injected engine

Roughly $1000 for a 7 day rental. More per day for short rentals

(figure $200 per day for short rentals?)

Oil supplied. We supply gas. Bring jerry cans.

MILEAGE CALCULATION

Roughly 6 gallons per hour at open throttle = 22.8 L/hr

Open throttle speed of around 34mph = 55kmph

55 / 22.8 = 2.4 kmpl = 42 L per 100km or 0.42 L per km

**Assumptions**

- one day of truck/camp costs included ($600)

- assumed $200 per day boat rental. If surveying many lake adjacent sites, the per day rental fee would decrease.

- Estimated fuel cost at $1.50/litre

- set the maximum range at 250km. This will largely depend on fuel reserves.

1) Set LakWin_Cos = LWin_Min_D

2) Run python script in field calc

def reclass(a):

if a > 250000:

a = 999999 #set cost to high value to avoid zero values later

else:

a = 600 + 200 + a / 1000 * 0.42 * 1.5

return a

LakWin_Cos = reclass(!LakWin_Cos!)

### Final Lake Winnipeg Powerboat surface:

“LakWin_Cos” Field in MB_brand_hex_2020

## **River Canoe Routes**

**Overview:**

Manually created and priced canoe routes, identifying areas that can be reached by crews on multi-day canoe trips. Usually one-way, with pickup and dropoff points reached by some combination of truck, train, or float plane access. Pricing depends on multiple sites being surveyed along the route in order to combine pickup/dropoff costs.

### 7.1 Base data:

Manually selected from Wilderness Rivers of Manitoba by Hap Wilson and Stephanie Ackroyd [8].

### 7.2 Processing:

**MB River Access and River Routes (Canoe routes)** – Canoe routes were gathered from Wilderness Rivers of Manitoba book, and several online sources. Routes were chosen to be traversable by open canoe by paddlers with novice-intermediate experience (Class 1-2 rapids, or Class 3+ rapids with portage options). Lat/Lon Coordinates for river route start and end reaches were confirmed on NCR Toporama and/or Google Earth. Polylines for river routes were manually traced from water_course and water_body layers. These draw layers were dissolved and cleaned up using the vertex editor, so that each route was a single continuous polyline.

**Individual logistic and pricing:**

Look for canoe routes in AB and MB and determine the logistics of their use - where the access is, access method, access costs (e.g., if a float plane drop off or extraction). These are applied to the cost models for canoe accessible routes.

**Assiniboine River**

60km

3 days

3 surveys

Road to Road

$Cost= \frac{\$600 drop off+\$600 pick up+450*\left( 3*1.5 \right)}{3}$

$Cost=\$1075 per sample unit$

**Berens River**

175km

10 days

8 surveys

road on both ends

$Cost= \frac{\$600 drop off+\$600 pick up +\left( 10*1.5*450 \right)}{8}$

$Cost=\$993.75 per sample unit$

**Bird River**

55km

3 days

2 surveys (this is a loop, so one way distance is 27km)

Road on both ends

$Cost= \frac{\$600 drop off+\$600 pick up+450*\left( 3*1.5 \right)}{2}$

$Cost=\$1612.50 per sample unit$

**Bloodvein River**

225km

14 days

11 surveys

Floatplane drop off. Road at end.

$Cost= \frac{1637.37+\$600 pick up+450*\left( 14*1.5 \right)}{11}$

$Cost=\$1062.49 per sample unit$

**Cochrane River to Northern Seal**

90km

5 days

4 surveys

Landing strip on one end. Need float plane for other. (Transwest Air does not have Otter at Wollaston. Using Lynn Lake)

$Cost= \frac{3704.64 + 4657.15+\left( 5*1.5*450 \right)}{4}$

$Cost=\$2934.20 per sample unit$

**Deer River**

120km

7 days

6 surveys

Via Rail on both ends

$Cost= \frac{\left( \$790 train drop off+2*450 \right)+\left( \$790 train pick up+2*450 \right)+450*\left( 7*1.5 \right)}{6}$

$Cost=\$1350.83 per sample unit$

**Gammon River**

106km

7 days

5 surveys

Road on both ends

$Cost= \frac{\$600 drop off+\$600 pick up+\$450*\left( 7*1.5 \right)}{5}$

$Cost=\$1185 per sample unit$

**Grass River**

370km

24 days

18 surveys

Roads on both ends

$Cost= \frac{\$600 drop off+\$600 pick up+\$450*\left( 24*1.5 \right)}{18}$

$Cost=\$966.67 per sample unit$

**Hayes River – Norway House to Oxford House**

230km

10 days

11 surveys

Road drop off and floatplane egress

$Cost= \frac{3078.06+\$600 drop off+450*\left( 14*1.5 \right)}{11}$

$Cost=\$1193.46 per sample unit$

**Hayes River – Oxford House to York Factory**

380km

18 days

19 surveys

Float on both ends (from Thompson on both ends)

$Cost= \frac{3078.06 + 6138.25 + \left( 18*1.5*450 \right)}{19}$

$Cost=\$1124.54 per sample unit$

**Manigotagan River**

103km

7 days

5 surveys

Road on both ends

$Cost= \frac{\$600 drop off+\$600 pick up+\$450*\left( 7*1.5 \right)}{5}$

$Cost=\$1185 per sample unit$

**Oskar Lake Route**

20km

1 day

2 surveys

Road on both ends

$Cost= \frac{\$600 drop off+\$600 pick up+\$450*\left( 2 \right)}{2}$

$Cost=\$1050 per sample unit$

**Pigeon River**

160km

14 days

8 surveys

Float plane on both ends

$Cost= \frac{600+600+\left( 14*1.5*450 \right)}{8}$

$Cost=\$1331.25 per sample unit$

**Poplar River**

244km

10 days

12 surveys

Float plane on both ends

$Cost= \frac{3547.49+ 2988.37+\left( 10*1.5*450 \right)}{8}$

$Cost=\$1660.73 per sample unit$

**Sasaginnigak River / Leyond River**

200km

10 days

10 surveys

Float plane start, ends on Lake Winnipeg where a ferry can be obtained (cannot find price for ferry. Assuming $100)

$Cost= \frac{\$1568.96+\$100 ferry+\$600 pick up+\$450*\left( 10*1.5 \right)}{10}$

$Cost=\$901.90 per sample unit$

**Thlewiaza River**

170km

8 days

8 surveys

Float plane on both ends (Wollaston does not have Otters stationed this year. From Lynn Lake)

$Cost= \frac{4657.15+6393.05+\left( 8*1.5*450 \right)}{8}$

$Cost=\$2056.28 per sample unit$

**Little Churchill River**

110km

8 days

5 surveys

Float plane on both ends

$Cost= \frac{3062.74 + 4140.13+\left( 8*1.5*450 \right)}{5}$

$Cost=\$2520.57 per sample unit$

**Burntwood River**

110km

10 days

5 surveys

Float plane drop off. Truck Pick up

$Cost= \frac{\$1968.71 +\$600 pick up+\left( 10*1.5*450 \right)}{5}$

$Cost=\$1863.74 per sample unit$

**Souris River**

60km

3 days

3 surveys

Roads on both ends

$Cost= \frac{\$600 drop off+\$600 pick up+\$450*\left( 7*1.5 \right)}{5}$

$Cost=\$1185 per sample unit$

**Rocky River / Watson River**

80km

6 days

4 surveys

Float plane on both ends

$Cost= \frac{4317.10 + 4627.65 + \left( 6*1.5*450 \right)}{4}$

$Cost=\$3248.69 per sample unit$

**Big Sand Lake to Tadoule Lake**

260 km

10 days

8 surveys

Float plane on both ends

$Cost= \frac{4886.69 + 7886.00 + \left( 10*1.5*450 \right)}{8}$

$Cost=\$2440.34 per sample unit$

**Sandy Bay to Pukatawagan**

Unable to find records of logistics

**Pukatawagan to Leaf Rapids**

Unable to find records of logistics

### 7.3 Canoe routes dataset:

Shapefile outlining linear canoe routes, with per-site cost estimates in attributes

### 7.4 Expand linear canoe route data to containing waterbody polygons to identify accessible shorelines:

Cost Distance and Euclidean Distance Raster generation

1. Converted polygon of >20km^2^ lakes with 200m buffer to raster of value 1.
2. Use Cost Distance tool to create cost distance raster using roads layer as source cost unit is 1 km. Will convert to time and then monetary cost later.
3. Use Cost Distance tool to create another cost distance raster using “all_rivers” layer as source raster and “MB_connected_waterbody_accessible_100m” as a cost raster. Cost unit also 1 km. Clip raster to values under 85,000m.
4. Use Euclidean Distance tool with float operating aerodromes as sources to create distance raster for provincial extent. Used raster calculator to mask Euclidean distance values to mask raster.

**River detour model generation**

1. Convert new clipped raster to polygon, titled “detours_under_85km”
2. Clip polygon layer containing route data to “detours_under_85km” layer.
3. Perform a spatial join with the following parameters:

Target features =MB_boreal_hex_2020_UTM

Join features = clipped canoe route polygon

Output feature class = canoe_access_cost

Join operation = JOIN_ONE_TO_ONE

Keep all target features checked

Field Map of Join Features: delete all attributes except route cost. Right click and set merge rule to minimum.

Match option = Intersect

The resulting layer is the new cost surface hexagons for canoe route access.

### 7.5 Final canoe trip surface:

“canoetrip” attribute in MB_brandt_hex_2020

## **Flatwater models**

**Overview:** Identifies areas accessible over water from a road-accessible launch point. Models built for both canoe and powerboat access, spatial extents are similar, but cost models applied differently.

### 8.1 Base data:

Uses two previously modified datasets: “Accessible waterbodies” (5.6) and “year-round roads” (1.6)

### 8.2 Cost-distance raster generation for road accessible waterbodies:

1. “MB_connected_waterbody_accessible_100m” to raster
2. Merge MB and SK road layers, buffer them by 100m, and convert them to raster
3. Create new distance-from –road raster using cost-distance tool. Road raster is source raster, “MB_connected_waterbody_accessible_100m” is cost raster.
4. This is the cost distance raster we will use as a base for the powerboat model

### 8.3 Road access cost distances dataset:

Raster dataset covering the extent of all waterbodies in Manitoba that have have some portion within 100m of a road. Values of each pixel represent least-cost-distance over water to a road, in kms.

### 8.4 Flatwater Canoe road access cost model:

**Road access Canoe Assumptions:**

Cover 15km/day, works out to passing ~5 shoreline hexagons/day

Survey 1 in 5 hexagons

Complete 1 survey every day, with 1 day/trip buffer

Days on the water = N hexagons/5 +1

Hexes surveyed = N hexagons/5

Assume techs making loop. Same pickup/dropoff spot, as they can survey along lakeside both ways. Options for one-way may present themselves, but would have to be individually coded in.

**Excluding <600m wide islands from flatwater land and hex access**These are removed because they are too small to fit point count grids. As this portion of the cost surface is partially based on the amount of surveyable land available, these islands must be removed from the calculation.

(See “Surveyable_land” model in “flatwater” toolbox)

1. Flatwater_access_land dissolve, multipart to singlepart, to get rid of hexagon separations
2. Buffer Dissolve_singlepart land polygon by -300m
3. Select polygons in Dissolve_singlepart that overlap with Dissolve_singlepart_buffer.
4. Use “identity” tool on singlepart_merged_selection to separate back into multipart hexagons with hexagon OBJECTID
5. Dissolve by hexagon TARGET_ID, allowing multipart hexagons.

Select from Singlepart_merged_selection_identity only polygons with >= 720,000 square metres.

(Creates layer of hexagons with enough land for at least two blocks of SSUs)

**Road access flatwater canoe model:**

1. Use cost-distance raster to remove portions of waterbody polygons greater than 85 km from roads. Does not otherwise use cost distance values for trip pricing.
2. Uses the number of surveyable hexagons (hexagons containing at least 720,000 square meters adjoining each lake to estimate both trip distance and number of sites. This is contained in the “Lakes_w_accessible_land_area_720” layer
3. Create site_cost field in “Lakes_w_surveyable_land_720” layer
4. Apply the following formula to calculate the cost per site on each lake:

$Cost= \frac{\$600 drop off+\$600 pick up+\$450*\left( \frac{Nhexagons}{5}+1 \right)}{Nhexagons /5}$

Where Nhexagons is obtained by the “N_hexagons” field in the “Lakes_w_surveyable_land_720” layer. Explanation of this formula is in the “MB_formulae” document.

1. Selected polygons of over 20 square kilometers. (arbitrary, but based on inclusion of sections of Nueltin Lake.
2. Selected hexagons that fell within 250m of negative buffered lakes (50m to counteract the -50m buffer on the lake polygons, +200m to for a walkable distance to access hexagons near the lakes.
3. Using Select by location between hexagons and the ORIGINAL waterbodies layer, deleted hexagons that fell completely within water polygons, ie, hexagons that contained only water.

   The result is the final “Flatwater_access_hex” layer.
4. Using erase tool, removed water covered area from “flatwater_access_hex” & calculated area of remaining polygons. Result is “flatwater_access_land” layer, which gives how much land is contained in each flatwater access hexagon
5. Spatial join of “flatwater_access_land” to “lakes_over_20km_buff200”

Result is layer of lake polygons, each with the amount of land accessible in adjacent hexagons by canoe once the lake is accessed. Add “Canoe_access” field and categorize these lakes by road, floatplane, train, minor river detour, and major river detour.

### 8.5 Final Flatwater canoe surface:

“Canoeroad” attribute in MB_brand_hex_2020

### 8.6 Flatwater powerboat road access cost model:

Distance limited to fuel rather than time, cost of access assessed individually rather than with “bulk discount” as with canoe access. However, if multiple sites selected on one lake, real cost can go down.

Cost formula is combination of pickup & dropoff costs, daily professional and boat rental costs, and fuel costs.

Professional costs assume cost of $450/day for a crew and multiplier of 1.4 included for lost opportunity cost. The number of days per site is equal to the least-cost distance to the road, /50, rounded up. This estimate is based off an assumed maximum daily travel distance of 100km (4 hours at 25 km/hr), and the need to double travel distances to include the return trip.

$$Cost= drop off+ pick up+[prof+boat rental]*days+fuel$$

Where: professional costs = $\left( \$450*1.4 \right)$

Boat rental = $\left( \$150 \right)$

Pickup and dropoff = ($600 X2)

Fuel = $(\frac{\$0.48}{Nkm})$

Days = $(\frac{Nkm}{50},rounded up)$

**Applying the powerboat access model to cost-distance raster:**

1. Select polygons in “MB_sask_accessible_waterbodies” layer that intersect with “main_and_summer_roads” layer to create “road_access” polygons
2. Convert “road_access” polygons to points using “generate points along lines” tool
3. Use “extract to point” tool to extract values from road access cost distance model to newly created point layer.
4. Spatial join of point layer to hexagon layer, with join statistics set to “minimum”, and search distance set to 200m.
5. Rename the resulting join field “road access distance”
6. Create new field titled “PB_cost”. Apply the following formula to calculate cost estimate for each hexagon
7. $Cost= \frac{\$600 drop off+\$600 pick up+\left[ prof, \left( \$450*1.4 \right)+boat rental\left( \$150 \right) \right]* days, (\frac{Nkm}{50},rounded up)+fuel (\frac{\$0.48}{Nkm})}{1}$

Explanation of this formula is in the “MB_formulae” document. Calculation may need to be done in R or Microsoft excel to account for whole number rounding to obtain trip day estimate.

1. The resulting PB_cost field can be integrated into the MB_boreal_hex layer

Application of powerboat trip cost model

### 8.7 Final powerboat flatwater surface:

“Powerboat” field in MB_brandt_hex_2020 dataset

# **Combining cost estimates**

**MB Boreal Hexagons –** From the Boreal Monitoring Strategy 5km Hexagon layer, clipped to the MB Boreal.

**MB_Brandt_hex_2020 Layer**

- Results of all models were joined to the Brandt Boreal Hexagons layer.
- Values of 999999 were used when a transportation method was not available for a hexagon
- Minimum costs per hexagon were based on a comparison of 13 cost fields (see formula below)
- An overall minimum cost was calculated in the Min_Cost field using the following python formula:

min([ !canoe_cost!, !truck_cost!, !win_road_c!, !min_hel_co!, !atv_cost!, !ViaTra_cst!, !LakWin_Cos!, !powerboat!, !Min_Sham_Y!, !canoetrip!, !canoeroad!, !keewatin!, !float_new!])

# **Graphical Summaries of data Processing**


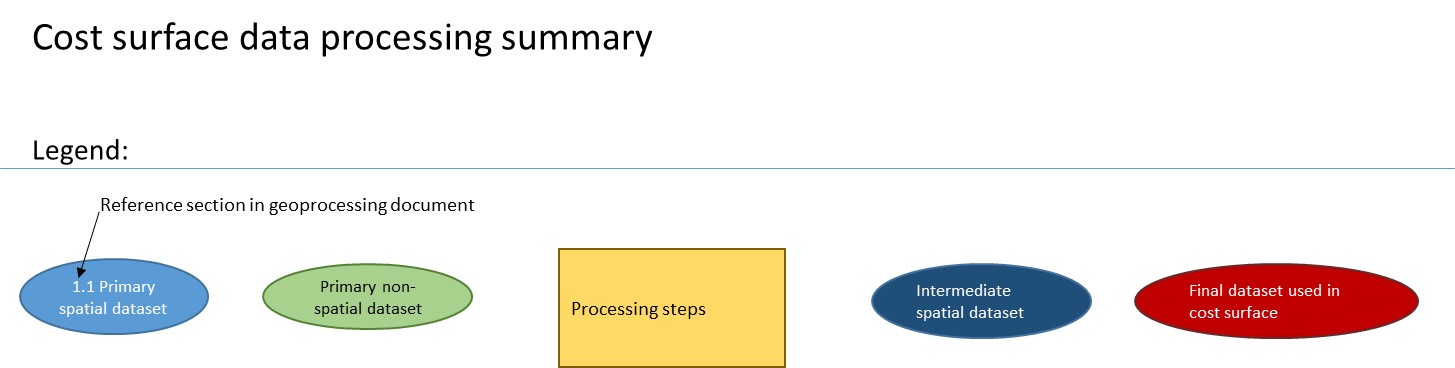


# **
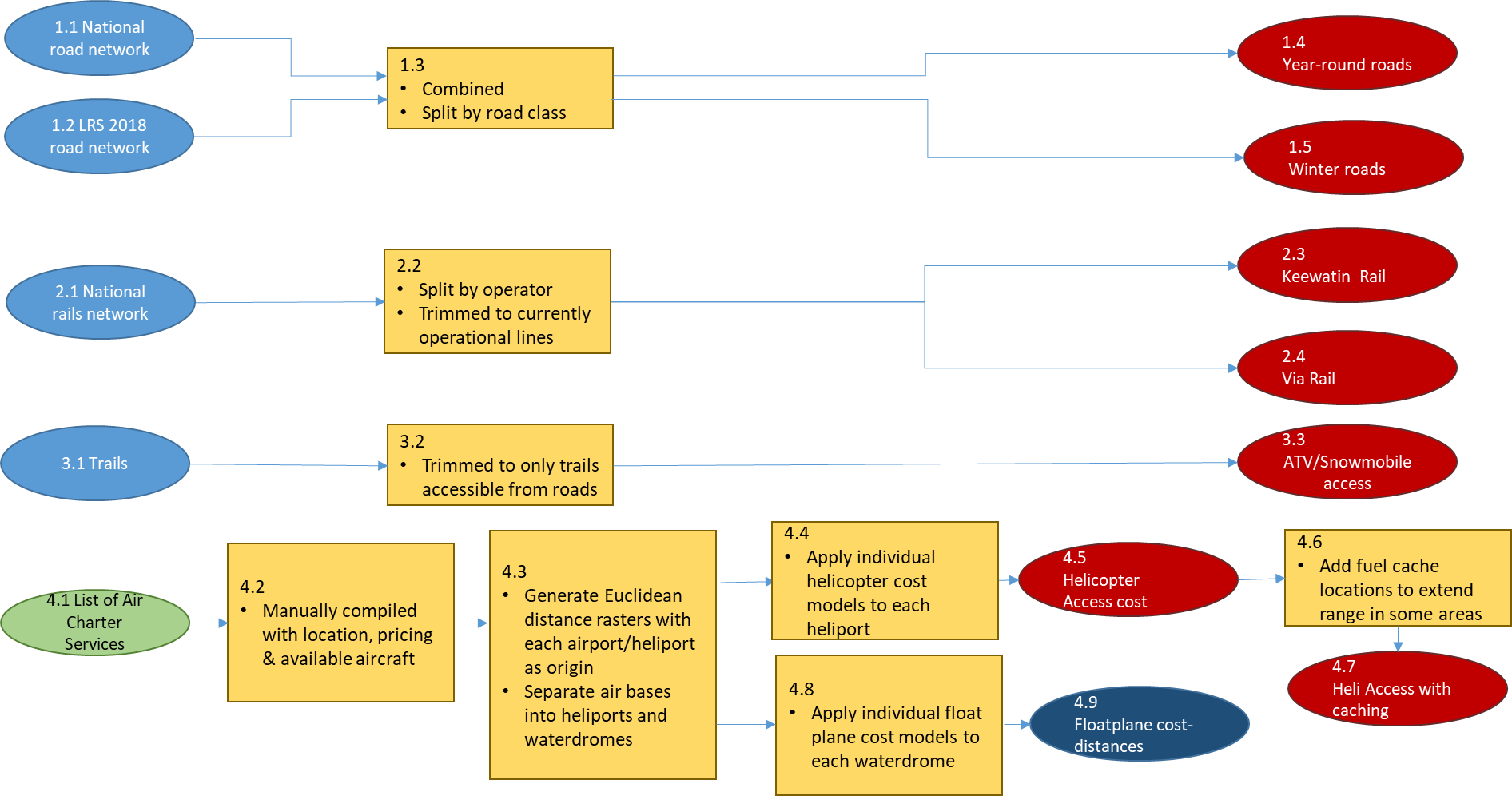
**


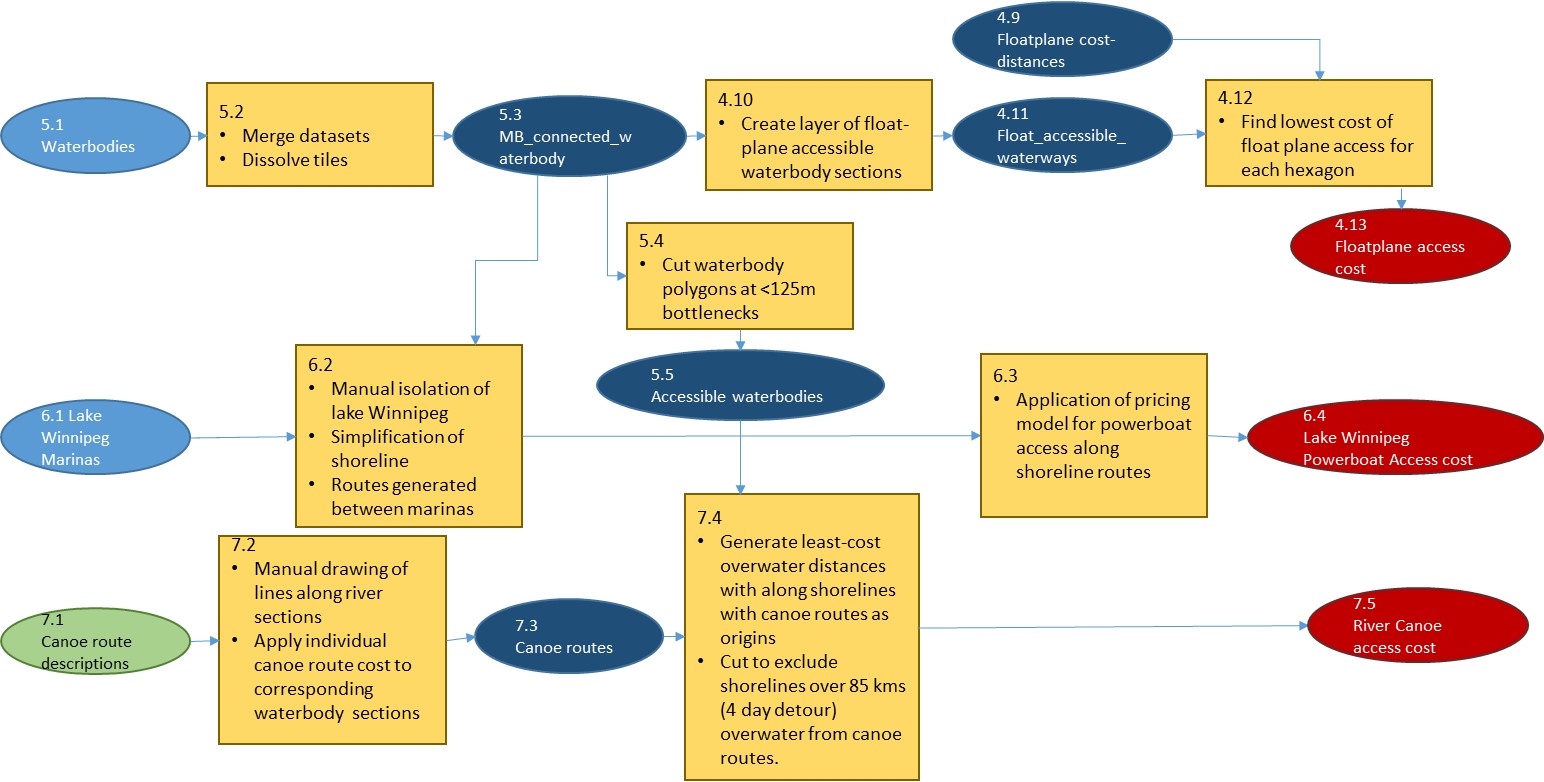


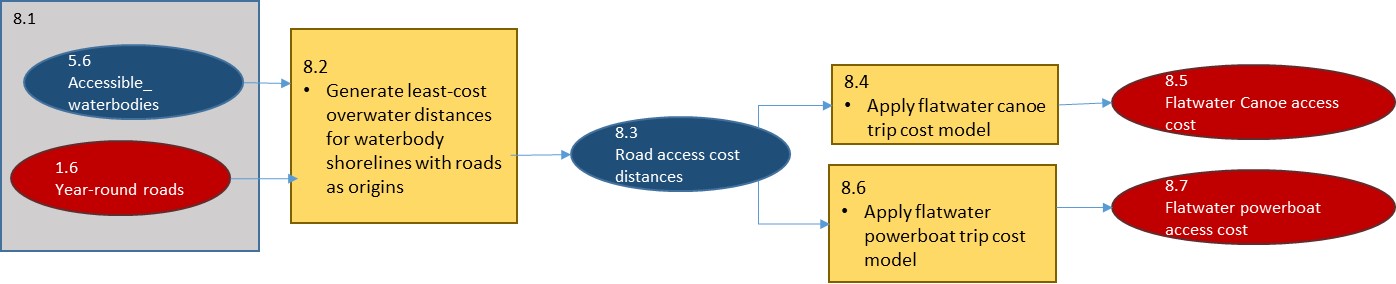


# **References:**

1. Manitoba Infrastructure, Planning and Design Branch, Geographic and Records Management Section. LRS road network; 2018 [cited 2020 May 10]. Database: Manitoba Land Use Initiative [Internet]. Available from: <http://mli2.gov.mb.ca/roads_hwys/shp_zip_files/trn_lrs_highway_network_2018_shp.zip>
2. Manitoba Conservation, Lands and Geomatics Branch. Manitoba-National Road Network; 2008[cited 2020 May 11]. Database: Manitoba Land Use Initiative [Internet]. Available from: <http://mli2.gov.mb.ca/roads_hwys/shp_zip_files/nrn_rrn_mb_shp_en.zip>
3. Government of Canada, Natural Resources Canada, Earth Sciences Sector. Track_segment_1.shp; 2017 [cited 2020 May 10]. Database: Transport networks in Canada CanVec – Transport Features [Internet]. Available from: <http://ftp.maps.canada.ca/pub/nrcan_rncan/vector/canvec/shp/Transport/canvec_50K_MB_Transport_shp.zip>
4. Government of Canada, Natural Resources Canada, Earth Sciences Sector. Trail1.shp; 2017 [cited 2020 May 10]. Database: Transport networks in Canada CanVec – Transport Features [Internet]. Available from: <http://ftp.maps.canada.ca/pub/nrcan_rncan/vector/canvec/shp/Transport/canvec_50K_MB_Transport_shp.zip>
5. Government of Canada, Public Works and Government Services Canada. List of Interested Suppliers for Air Charter Services; 2020 [cited 10 May 2020]. In: buyandsell.gc.ca [Internet]. Available from: <https://buyandsell.gc.ca/procurement-data/tender-notice/PW-ZL-102-25299/list-of-interested-suppliers>
6. Government of Canada, Natural Resources Canada, Earth Sciences Sector. water_course1.shp, water_body2.shp; 2017 [cited 2020 May 10]. Database: Lakes and rivers in Canada CanVec – Hydro Features [Internet]. Available from: <http://ftp.maps.canada.ca/pub/nrcan_rncan/vector/canvec/shp/Hydro/canvec_250K_MB_Hydro_shp.zip>
7. Government of Canada, Natural Resources Canada, Earth Sciences Sector. Marina_0.shp; 2017 [cited 2020 May 10]. Database: Transport networks in Canada CanVec – Transport Features [Internet]. Available from: <http://ftp.maps.canada.ca/pub/nrcan_rncan/vector/canvec/shp/Transport/canvec_50K_MB_Transport_shp.zip>
8. Wilson H, Aykroyd S. Wilderness Rivers of Manitoba: Journey by Canoe Through the Land Where the Spirit Lives. 2^nd^ ed. Erin: Boston Mills Press; 2004.
