# Supplementary material for: A cost efficient spatially balanced hierarchical sampling design for monitoring boreal birds incorporating access costs and habitat stratification
Source: PLoS One. 2020 Jun 16;15(6):e0234494. doi: 10.1371/journal.pone.0234494 (PMC7297386; doi:10.1371/journal.pone.0234494)
Supplement: Attachment — Submitted filename: PONE-D-19-35579_reviewer_s.pdf [file pone.0234494.s009.pdf]

PLOS ONE

A cost efficient spatially balanced hierarchical sampling design for monitoring boreal birds incorporating access costs and habitat stratification

--Manuscript Draft--

**Manuscript Number:**	PONE-D-19-35579
**Article Type:**	Research Article
**Full Title:**	A cost efficient spatially balanced hierarchical sampling design for monitoring boreal birds incorporating access costs and habitat stratification
**Short Title:**	A cost efficient spatially balanced hierarchical sampling design for monitoring in remote areas
**Corresponding Author:**	Steven Van Wilgenburg<br>Canadian Wildlife Service<br>Saskatoon, CANADA
**Keywords:**	optimal sampling; boreal; monitoring; birds; stratification; spatially balanced sampling; cost efficient
**Abstract:**	Predicting and mitigating impacts of climate change and development within the boreal biome requires a sound understanding of factors influencing the abundance, distribution, and population dynamics of species inhabiting this vast biome. Unfortunately, the limited accessibility of the boreal biome has resulted in sparse and spatially biased sampling, and thus our understanding of boreal bird population dynamics is limited. To implement effective conservation of boreal birds, a cost effective approach to sampling the boreal will be needed. Our objective was to devise a sampling scheme for monitoring boreal birds that would improve our ability to model species-habitat relationships and monitor changes in population size and distribution. A statistically rigorous design to achieve these objectives would have to be spatially balanced and hierarchically structured with respect to ecozones, ecoregions and political jurisdictions. Therefore, we developed a multi-stage hierarchically-structured sampling design known as the Boreal Optimal Sampling Strategy (BOSS) that included cost constraints, habitat stratification, and optimization to provide a cost-effective alternative to other common monitoring designs. Our design provided similar habitat and spatial representation to habitat stratification and equal-probability spatially balanced designs, respectively. Not only was our design able to achieve the desired habitat representation and spatial balance necessary to meet our objectives, it was also significantly less expensive (1.3−2.6 times less) than alternative designs we considered. Field implementation of the design in three vastly different regions over three field seasons showed that the approach can be implemented in a wide variety of logistical scenarios and ecological conditions. We provide worked examples and scripts to allow our approach to be implemented or adapted elsewhere. We also provide recommendations for possible future refinements to our approach, but recommend that our design be implemented to provide unbiased information to assess the status of boreal birds and inform conservation and management within the boreal biome.
**Order of Authors:**	Steven Van Wilgenburg
	C. Lisa Mahon
	Greg Campbell
	Logan McLeod
	Margaret Campbell
	Dean Evans
	Wendy Easton
	Charles M Francis
	Samuel Haché
	Craig S Machtans
	Rhiannon Pankratz

Powered by Editorial Manager® and ProduXion Manager® from Aries Systems Corporation

	Rich Russell
	Adam C Smith
	Peter Thomas
	Judith D Toms
	Junior A Tremblay
**Additional Information:**	
**Question**	**Response**
**Financial Disclosure**<br><br>Enter a financial disclosure statement that describes the sources of funding for the work included in this submission. Review the submission guidelines for detailed requirements. View published research articles from *PLOS ONE* for specific examples.<br><br>This statement is required for submission and **will appear in the published article** if the submission is accepted. Please make sure it is accurate.<br><br>**Unfunded studies**<br>Enter: *The author(s) received no specific funding for this work.*<br><br>**Funded studies**<br>Enter a statement with the following details:<br>• Initials of the authors who received each award<br>• Grant numbers awarded to each author<br>• The full name of each funder<br>• URL of each funder website<br>• Did the sponsors or funders play any role in the study design, data collection and analysis, decision to publish, or preparation of the manuscript?<br>• **NO** - Include this sentence at the end of your statement: *The funders had no role in study design, data collection and analysis, decision to publish, or preparation of the manuscript.*<br>• **YES** - Specify the role(s) played.<br><br>* typeset	The author(s) received no specific funding for this work.
**Competing Interests**<br><br>Use the instructions below to enter a competing interest statement for this submission. On behalf of all authors,	The authors have declared that no competing interests exist.

disclose any competing interests that could be perceived to bias this work—acknowledging all financial support and any other relevant financial or non-financial competing interests.<br><br>This statement **will appear in the published article** if the submission is accepted. Please make sure it is accurate. View published research articles from *PLOS ONE* for specific examples.<br><br>**NO authors have competing interests**<br><br>Enter: *The authors have declared that no competing interests exist.*<br><br>**Authors with competing interests**<br><br>Enter competing interest details beginning with this statement:<br><br>*I have read the journal's policy and the authors of this manuscript have the following competing interests: [insert competing interests here]*<br><br>* typeset	
**Ethics Statement**<br><br>Enter an ethics statement for this submission. This statement is required if the study involved:<br><br>• Human participants<br>• Human specimens or tissue<br>• Vertebrate animals or cephalopods<br>• Vertebrate embryos or tissues<br>• Field research<br><br>Write "N/A" if the submission does not require an ethics statement.<br><br>General guidance is provided below. Consult the submission guidelines for detailed instructions. **Make sure that all information entered here is included in the Methods section of the manuscript.**	N/A

**Format for specific study types**<br><br>**Human Subject Research (involving human participants and/or tissue)**<br>• Give the name of the institutional review board or ethics committee that approved the study<br>• Include the approval number and/or a statement indicating approval of this research<br>• Indicate the form of consent obtained (written/oral) or the reason that consent was not obtained (e.g. the data were analyzed anonymously)<br><br>**Animal Research (involving vertebrate animals, embryos or tissues)**<br>• Provide the name of the Institutional Animal Care and Use Committee (IACUC) or other relevant ethics board that reviewed the study protocol, and indicate whether they approved this research or granted a formal waiver of ethical approval<br>• Include an approval number if one was obtained<br>• If the study involved *non-human primates*, add *additional details* about animal welfare and steps taken to ameliorate suffering<br>• If anesthesia, euthanasia, or any kind of animal sacrifice is part of the study, include briefly which substances and/or methods were applied<br><br>**Field Research**<br><br>Include the following details if this study involves the collection of plant, animal, or other materials from a natural setting:<br>• Field permit number<br>• Name of the institution or relevant body that granted permission	
**Data Availability**<br><br>Authors are required to make all data underlying the findings described fully available, without restriction, and from the time of publication. PLOS allows rare exceptions to address legal and ethical concerns. See the PLOS Data Policy and FAQ for detailed information.	Yes - all data are fully available without restriction

A Data Availability Statement describing where the data can be found is required at submission. Your answers to this question constitute the Data Availability Statement and **will be published in the article**, if accepted.<br><br>**Important:** Stating 'data available on request from the author' is not sufficient. If your data are only available upon request, select 'No' for the first question and explain your exceptional situation in the text box.<br><br>Do the authors confirm that all data underlying the findings described in their manuscript are fully available without restriction?	
**Describe where the data may be found in full sentences. If you are copying our sample text, replace any instances of XXX with the appropriate details.**<br><br>• If the data are **held or will be held in a public repository**, include URLs, accession numbers or DOIs. If this information will only be available after acceptance, indicate this by ticking the box below. For example: *All XXX files are available from the XXX database (accession number(s) XXX, XXX.*).<br>• If the data are all contained **within the manuscript and/or Supporting Information files**, enter the following: *All relevant data are within the manuscript and its Supporting Information files.*<br>• If neither of these applies but you are able to provide **details of access elsewhere**, with or without limitations, please do so. For example:<br><br>*Data cannot be shared publicly because of [XXX]. Data are available from the XXX Institutional Data Access / Ethics Committee (contact via XXX) for researchers who meet the criteria for access to confidential data.*<br><br>*The data underlying the results presented in the study are available*	The data underlying the results presented in the study are available from the primary author (SVW) who may be contacted at steven.vanwilgenburg [at] Canada.ca. Data will be placed on the Research Gate profile of SVW if the manuscript is accepted.

Powered by Editorial Manager® and ProduXion Manager® from Aries Systems Corporation

from (include the name of the third party and contact information or URL).<br>• This text is appropriate if the data are owned by a third party and authors do not have permission to share the data.<br><br>* typeset	
Additional data availability information:	

Manuscript
Click here to download Manuscript BOSS _PLoS ONE_Bibliography_converted.docx

RESEARCH ARTICLE

A cost efficient spatially balanced hierarchical sampling design for monitoring boreal birds incorporating access costs and habitat stratification

Steven L. Van Wilgenburg[1]*, C. Lisa Mahon[2,3], Greg Campbell[4], Logan McLeod[2], Margaret Campbell[2], Dean Evans[5], Wendy Easton[6], Charles M. Francis[7,8], Samuel Haché[5], Craig S. Machtans[2], Rhiannon F. Pankratz[5], Rich Russell[7], Adam C. Smith[7,8], Peter Thomas[9], Judith D. Toms[3,10], Junior A. Tremblay[11]

[1]Environment and Climate Change Canada, Saskatoon, SK, Canada

[2]Environment and Climate Change Canada, Whitehorse, YT, Canada

[3]Department of Biological Sciences, University of Alberta, Edmonton, AB, Canada

[4]Environment and Climate Change Canada, Mount Pearl, NL, Canada

[5]Environment and Climate Change Canada, Yellowknife, NT, Canada

[6]Environment and Climate Change Canada, Vancouver, BC, Canada

[7]Environment and Climate Change Canada, Ottawa, ON, Canada

[8]Department of Biology, Carlton University, Ottawa, ON, Canada

[9]Environment and Climate Change Canada, Sackville, NB, Canada

[10]Environment and Climate Change Canada, Edmonton, AB, Canada

[11]Environment and Climate Change Canada, Quebec City, QC, Canada

*Corresponding author: steven.vanwilgenburg@Canada.ca (SVW)

Abstract

Predicting and mitigating impacts of climate change and development within the boreal biome requires a sound understanding of factors influencing the abundance, distribution, and population dynamics of species inhabiting this vast biome. Unfortunately, the limited accessibility of the boreal biome has resulted in sparse and spatially biased sampling, and thus our understanding of boreal bird population dynamics is limited. To implement effective conservation of boreal birds, a cost effective approach to sampling the boreal will be needed. Our objective was to devise a sampling scheme for monitoring boreal birds that would improve our ability to model species-habitat relationships and monitor changes in population size and distribution. A statistically rigorous design to achieve these objectives would have to be spatially balanced and hierarchically structured with respect to ecozones, ecoregions and political jurisdictions. Therefore, we developed a multi-stage hierarchically-structured sampling design known as the Boreal Optimal Sampling Strategy (BOSS) that included cost constraints, habitat stratification, and optimization to provide a cost-effective alternative to other common monitoring designs. Our design provided similar habitat and spatial representation to habitat stratification and equal-probability spatially balanced designs, respectively. Not only was our design able to achieve the desired habitat representation and spatial balance necessary to meet our objectives, it was also significantly less expensive (1.3−2.6 times less) than alternative designs we considered. Field implementation of the design in three vastly different regions over three field seasons showed that the approach can be implemented in a wide variety of logistical scenarios and ecological conditions. We provide worked examples and scripts to allow

our approach to be implemented or adapted elsewhere. We also provide recommendations for possible future refinements to our approach, but recommend that our design be implemented to provide unbiased information to assess the status of boreal birds and inform conservation and management within the boreal biome.

Introduction

Tackling ongoing [1-3] and projected [4,5] global biodiversity losses will require difficult decisions about resource allocation and the need to consider when, where, and how to focus conservation efforts. Species conservation is often most successful and least expensive when implemented early, before species require dedicated recovery efforts [6]. Early identification of species declines should improve the likelihood of successful conservation. In addition, developing effective conservation strategies requires identifying the locations and factors contributing to species declines [7]. Sparse or biased data may inaccurately identify which species require conservation actions or incorrectly identify key drivers, resulting in misdirected or ineffective conservation actions [8]. Thus, well-designed ecological monitoring is necessary to effectively prioritize conservation activities [9-11].

Despite the need for monitoring data, even comparatively well monitored taxa such as terrestrial birds have significant gaps in species coverage that hinder effective status and trend assessments and associated conservation actions [12]. In North America, many species of terrestrial birds are primarily monitored using the North American Breeding Bird Survey (*hereafter* BBS; [12]). However, this survey, which is based on roadside surveys, mainly run by volunteers, has very limited coverage in remote northern locations such as the boreal forest [13,14]. Furthermore, much of the coverage for species that breed in the boreal forest is limited to the southern edges of their range, where population change may be quite different from elsewhere. The lack of data from most of the boreal forest may present significant risks to conservation given

expanding resource development [15-17] and projected climate change impacts on boreal birds [18].

Monitoring data from northern ecoregions are needed to inform conservation actions (e.g. prioritize selection for protected areas), contribute to management decisions (e.g. adaptive management), detect range shifts, assess threats to species (e.g. species response to developments), and meet legislative requirements. In Canada, monitoring data are required to support conservation under the Migratory Birds Convention Act (1994, c. 22), which includes informing listing decisions under Canada's Species at Risk Act (*hereafter* SARA; [19]), impact assessments required by the Impact Assessment Act [20], or assessments required by provinces or territories. Key criteria used by the Committee on the Status of Endangered Wildlife in Canada (*hereafter* COSEWIC) to recommend listing a species under SARA include decline in the total number of mature individuals, population size, and extent of species occurrence [21]. In addition, habitats and region specific estimates of species' abundance or density are required baseline data in environmental impact assessments [20]. To support these diverse requirements, multi-species bird monitoring should be capable of accurately capturing not only trends in species' population sizes, but also changes in both spatial and temporal variation in species status and distribution.

Given the breadth of these objectives and the large and remote areas to be monitored a cost effective sampling approach is required. As most of these locations have limited road networks [22] with few human settlements, there is little potential for volunteer surveys [23]. Similarly, a simple, design-based sampling strategy (e.g., a randomized design in which sample units are selected with the same probabilities) is

logistically and financially impractical [9,24]. Instead, we propose a design-based sample design that integrates multiple approaches from the statistical sampling-theory literature including spatially balanced sampling [25,26], stratified sampling [27-30], cost-constrained sampling [29] and optimization [31]. The integration of these approaches optimizes the spatial balance and habitat representation at minimal program costs, for the entire boreal landbird community.

Here, we describe a cost-effective hierarchically structured unequal probability sampling design known as the Boreal Optimal Sampling Strategy (BOSS). We compared our design against three competing spatially balanced sampling designs: a purely spatial (equal-probability) design, a habitat stratification design, and a design that minimized access costs. We hypothesized that the BOSS design should provide the second best habitat representation after the habitat stratification design since we incorporated habitat in the inclusion probabilities. We also hypothesized that our BOSS design should rank as the second-least expensive alternative after the cost design owing to incorporation of costs within the inclusion probabilities. Specifically, our objectives are to: (1) outline the development of the BOSS sampling design; (2) describe BOSS design implementation in three ecologically distinct regions in Canada (Newfoundland and Labrador, Saskatchewan, Yukon Territory); (3) evaluate how the BOSS design meets spatial balance, habitat representation, and cost objectives in these three distinct regions; and 4) evaluate implementation costs in field trials.

Materials and methods

Sampling frame

We defined the target population as all adult individuals of terrestrial bird populations occupying an ecoregion, province/territory (*hereafter* jurisdiction), and Bird Conservation Region (BCR) within the boreal region of Canada [32] during the breeding season for a particular year. We defined the Primary Sampling Unit (PSU) as a 5 km diameter hexagonal grid cell. We generated a hexagonal grid of PSUs covering the entire sampling frame: the boreal region of Canada, plus a 100 km northern buffer (Fig 1). We used hexagonal geometry because it introduces fewer edge effects and performs well in nearest neighbour analyses since the distances between centroids are the same in all directions [33]. We included a northern buffer to allow the design to capture possible breeding range shifts associated with climatic changes in the north [34]. Each PSU was assigned a unique number/identifier. With the exclusion of PSUs that have cumulative inclusion probabilities of zero (e.g. exclusively open water), all PSUs in the sampling frame were available to be sampled and as a result, the BOSS sampling design can make valid inferences to entire populations of species inhabiting the boreal [35].

Stratification

We developed a hierarchical stratification scheme to estimate population variables at multiple spatial scales. At the first level, we stratified the sampling frame by the intersection of BCRs and jurisdictions (e.g., area of Yukon Territory within BCR 4-Northwestern Interior Forest). At the second level, we further stratified each BCR by jurisdiction combination by ecoregion (e.g., the area of Selwyn Mountains ecoregion within Yukon Territory within BCR 4), where ecoregions are a subdivision of an ecozone

characterized by similarity in surface forms, flora, fauna, hydrology, soil, and macro climate [36]. Regional monitoring programs as part of the BOSS design are defined by the area of each jurisdiction within a BCR. We set target sample sizes for the number of PSUs to select within an ecoregion using the process outlined below (see sample size allocation).

Sample size allocation

Optimal stratified sampling accounts for both the size of the stratum (e.g., land-area for geographic regions), and the variance within the stratum [37]. Greater sampling effort is allocated using Neyman allocation to strata that are larger and more variable to optimize the precision of the overall estimate [27,29]. Specifically, we calculated the sample size of PSUs (n) for a given jurisdiction using the following equation to allocate sampling effort between all (k) ecoregions within the jurisdiction:

$$n_e = N * \frac{Area_e * \sigma_e * r_e}{\sum_{e_{1...k}} Area_e * \sigma_e * r_e} \quad (1)$$

Where n_e = is the target sample size for a given ecoregion within a given jurisdiction, N = total target sample size to sample within the jurisdiction, $Area_e$ = area of ecoregion e, σ_e= is a measure of observed or predicted variation within ecoregion e for the variable(s) of interest, and r_e = described species richness of the bird community in ecoregion e (i.e. the number of all bird species with ranges overlapping the ecoregion derived from overlays of species range maps with the ecodistricts of Canada [36] by GeoInsight Corporation [38]). Since our goal is to monitor multiple species, employing a univariate measure of variance would provide an inadequate representation of variance [29]. We included species richness as a weighting factor on within-stratum variation (σ_e), to

increase sampling in ecoregions where more species occur and improve the (1) likelihood of adequately monitoring a greater number of species, and (2) ability to monitor and predict species distributions.

Many of the ecoregions in our sampling frame have little or no historic sampling from which we could derive estimates of spatial or temporal variance in the bird community. As an alternative, we used proxy variables that correspond with the spatial and/or temporal variance in bird abundance within an ecoregion to set relative PSU sample size targets for the ecoregion. We analyzed the proxy variables [29] representing spatial and temporal variation in bird abundance and distribution by calculating a measure of multivariate dispersion (variance estimate) for each ecoregion following [39,40]. We used several proxy variables to calculate multivariate dispersion given well described links between species abundance and distribution and spatial and temporal climatic variation [41], vegetation [41,42] and variation in forest age [42,43]. Specifically, we used the following proxy variables: (1) variance of mean temperatures for May-July across years (1981-2014); (2) variance of total precipitation for May-July across years (1981-2014); (3) standard deviation of annual burn rates (% area burned by wildfire per year) between 1980 and 2015 within an ecodistrict; (4) richness of landcover classes [44] within an ecodistrict; (5) standard deviation of elevation; and (6) percent area of open water since riparian edges significantly increase avian species richness [45]. We calculated variance in May-July (1) temperatures and (2) precipitation from monthly gridded (0.5 x 0.5 degrees) values from the climate datasets of Harris et al. [46]. We calculated the between year standard deviation of burn rates from data obtained from [47]. Since burn rates from [47] were calculated in sample units of 10,000

km^2 hexels, we first calculated the between year standard deviation of burn rates within hexels, and derived the mean of those values for ecodistricts. Landcover of Canada 2010 [44] is a 250 m/30 m resolution land cover layer of 15 land cover classes from 13,350 Landsat-7 satellite images taken between 2009 and 2011. Ecodistricts (subdivisions of ecoregions characterized by distinctive landforms, relief, geology, soils, water bodies, flora and fauna [36]) were treated as sampling units to allow us to calculate dispersion within ecoregions.

Sampling design

We developed a two-stage sampling design in which the first stage of the design involved selection of PSUs from ecoregions within the sampling frame and the second stage involved selection of Secondary Sampling Units (SSU) within the selected PSUs (Fig 2). The SSU represent on-the-ground locations where point count surveys [48] will be conducted using trained observers and/or Autonomous Recording Units [49].

Selection of primary sampling units

To select PSU within each jurisdiction (Stage 1) we first needed to develop an approach to combine the following three design components: access costs, habitat representation, and spatial balance. We followed the steps outlined below in each of our three jurisdictions (Newfoundland and Labrador, Saskatchewan, and Yukon Territory) to integrate all components of our sampling design in areas spread widely across our sampling frame (Fig 3). First, we created separate cost surfaces for logistically feasible access methods within each of our jurisdictions. Specifically, we developed spatially explicit models predicting the cost to access a given PSU assuming access from roads (trucks), helicopters, float planes, snowmobile and/or all terrain vehicles as appropriate

for a given jurisdiction (see examples in Supporting Information S2). The resulting cost models were represented as pixel based (250 x 250m) estimates of cost, from which we subsequently calculated the average cost of access for all pixels within a PSU separately for each access type (trucks, helicopters, float planes). We assumed that the lowest cost access method will generally be used to access a given PSU and calculated the minimum cost across all access cost surfaces. We subsequently used this value as the estimated access cost for each PSU.

Second, we calculated inclusion probabilities based on landcover classes (hereafter habitat classes). We calculated habitat-based inclusion probabilities in an attempt to achieve balanced representation of habitat classes within an ecoregion. Inclusion probabilities for habitat classes used a weighted sampling approach to balance sampling across common (high occurrence/high proportion) and uncommon (low occurrence/low proportion) habitats within an ecoregion. Weighted habitat inclusion probabilities were calculated as follows:

$$p_{habitat_{pie}} = \frac{\frac{1}{\#\ of\ Habitat\ Classes\ in\ ecoregion_e}}{Area_{ie}} \quad (2)$$

Where the inclusion probability for a given pixel p of habitat class i within a given ecoregion ($p_{habitat_{ice}}$) was a function of the inverse of the number of habitat classes within the ecoregion divided by the area of habitat i within ecoregion e. Our weighted habitat inclusion probability thus results in the cumulative probability of sampling a given habitat within an ecoregion being equal among all habitat classes despite differences in habitat area.

Third, we combined access costs with weighted habitat inclusion probabilities to derive our final summed value for inclusion probabilities for each PSU as follows:

$$P_{SU_k} = \sum \frac{p_{habitat_{pie}} * \frac{1}{\sqrt{cost_k}}}{\sum p_{habitat_{pie}} * \frac{1}{\sqrt{cost_k}}} \quad (3)$$

Where P_{SU_k} = the sum of all inclusion probabilities within a given primary sample unit (PSU) k, $P_{habitat_{pie}}$ = area weighted selection probabilities for habitat class i within pixel p in ecoregion e, and cost for a given PSU was the minimum access cost for sample unit k (as described above). Cost was included as the inverse square root of costs to preferentially (all else being equal) favour sampling at sites that are less expensive to sample in accordance with optimal allocation theory [50].

Fourth, we used unequal-probability sampling based on the calculated inclusion probabilities (P_{SU_k}) to draw a spatially balanced sample within each ecoregion using Generalized Random Tessellation Stratified sampling (GRTS; [25]). The random draw to select PSU within ecoregions in each jurisdiction uses PSU sample size targets for each ecoregion (where ecoregion boundary was clipped to the jurisdiction boundary). We included a 20% oversample of primary sampling units for each ecoregion to allow substitution of inaccessible or unsafe PSUs (e.g. cliffs or other features not visible in the available input layers). All PSUs were labeled with their draw order and oversample PSUs were additionally labeled as oversamples. Should a PSU be inaccessible or unsafe to survey, the oversample and draw order can be used to select a replacement PSU that retains the spatial balance of the sample [25].

Finally, we derived an approximately optimal draw of PSU by a combination of repeated random sampling and multi-criteria evaluation. Specifically, we propose that multiple random unequal-probability GRTS samples can be obtained for which the predicted sampling costs and metrics of habitat representation and spatial balance can be derived. The iteration resulting in the highest combined representativeness of habitat sampling combined with spatial balance for the lowest predicted sampling cost should provide an approximately optimal design. As a metric of habitat representation, we calculated the sum of squared differences (SSD) between the area of each habitat class within all PSUs in a randomized draw versus the proportional area of each habitat class. As a metric of spatial balance, we use Pielou evenness (PE in eq. 4 below) to represent the even spread of PSU across the sample frame [28]. In order to evaluate the optimal draw across multiple (1…j) iterations, we propose evaluating which iteration minimizes predicted costs while maximizing the weighted sum of the two metrics:

$$\left(\left(1-\left(SSD_i-\min\left(SSD_{i\ldots j}\right)\right)\right)\bullet w_h\right)+\left(w_s\bullet PE_i\right) \quad (4)$$

Where SSD for sample draw i is relativized to the minimum SSD across draws 1 through j, and subtract the resulting value from 1 so that the metric scales from 0–1 and thus a value of 1 would represent perfect representation of habitat composition within the draw. We applied equal weights to both the habitat (w_h) and spatial balance (w_s) components as we did not want either component to be more heavily valued than the other in our final design (i.e. $w_h = w_s$ = 0.5). This approach can be generalized by allowing weights to vary between habitat representation and spatial representation if the

desire was to emphasize habitat representation (e.g. environmental impact assessments) versus distribution modeling or vice versa.

Selection of secondary sampling units

To select secondary sample units (SSU) within each PSU in each jurisdiction (Stage 2), we first created a uniform (systematic) grid of potential secondary sample unit locations at 300 m spacing over the extent of each ecoregion. Each SSU was separated by 300 m since the effective detection radius for most boreal songbirds are less than 150 meters [48] and therefore double counting should be infrequent for most species. We used the grid of SSU to query the habitat-based inclusion probabilities. We selected a stratified random sample of four SSU where higher inclusion probability values indicating rare/uncommon habitat classes influenced the selection of SSU. We used the four SSU selected during this draw as plot centroids and the eight SSUs surrounding the plot centroid to construct plots of nine SSU for sampling (see Fig 2). We set a sampling target of two plots of nine SSUs per PSU, but drew a sample of four plots of SSU per PSU to create an oversample in case of inaccessibility (e.g. across a large water barrier) or safety concerns (e.g. hazardous terrain or aggressive wildlife). In addition, we aimed for two plots of nine SSUs so that a team of two staff can complete one PSU per day in most circumstances. Furthermore, having two survey plots allows staff to have a partner(s) within the same PSU to act as a first responder if needed. In addition, our PSU size of 5 km diameter was specifically selected to ensure field staff would be working within a reasonable walking or flight distance if assistance or emergency response is required.

Data collection

We tested the BOSS design within three jurisdictions (Newfoundland and Labrador [NL], Saskatchewan [SK], and Yukon Territory [YT]) across the boreal biome of Canada during 2017, 2018, and 2019. Newfoundland and Labrador is located at the eastern edge of the boreal region within BCR 7 -Taiga Shield and Hudson Plains and BCR 8-Boreal Softwood Shield (Fig 1). Saskatchewan is located in the center within BCR 6, BCR 8, and BCR 7 (see Fig 1); while the Yukon Territory is located at the northwestern edge within BCR 4-Northwestern Interior Forest, BCR 6-Boreal Taiga Plains, and BCR 3-Arctic Plains and Mountains. Jurisdictions were also ecologically distinct, differing in their geology, topography, vegetation, terrestrial bird diversity, and climate as indicated by the number of ecozones (NL: 2; SK: 3; YT: 3), ecoregions (NL: 23; SK: 8; YT: 22), and elevation range (NL: 0−1,652 m; SK: 204−823 m; YT: 0−5,900 m). Differences in the location of mountain ranges, rivers and lakes, human settlements, and primary and secondary road networks within each jurisdiction present distinct logistical challenges and therefore useful comparative test cases. To conduct point count surveys at SSU within selected PSU we followed standard recommended protocols of Ralph et al. (1993) and Matsuoka et al. (2014) and conducted point count surveys during suitable weather conditions within 4-5 hours after sunrise, from the last week of May to the first week of July. We conducted 10 minute point counts using trained observers following protocols outlined by Matsuoka et al. [48] or we analysed 10 minute recordings from Autonomous Recording Units [49]. Draft standards and protocols for describing Autonomous Recording Unit deployment and retrieval schedules, recording schedules, and manual transcription are available from the authors.

We sampled 295 PSUs using the BOSS design between 2017−2019 in each of Newfoundland and Labrador (n = 69), Saskatchewan (n = 103) and Yukon Territory (n = 123). Three PSUs in Saskatchewan had to be replaced with PSUs from the oversample owing to access and safety issues associated with open pit uranium mines. In Newfoundland and Labrador, we sampled 5 out of 9 ecoregions on the island of Newfoundland, with all ecoregions being in BCR 8. PSUs were sampled in the Avalon Forest ecoregion (n = 13, Central Newfoundland (n = 1), Maritime Barrens (n = 8), South Avalon-Burin Oceanic Barrens (n = 23), and Southwestern Newfoundland (n = 24). We sampled seven out of eight ecoregions within Saskatchewan, including Selwyn Lake Upland (BCR 7, n = 12), Tazin Lake Upland (BCR 7, n=9), Athabasca Plain (BCR 8, n= 19), Churchill River Upland (BCR 8, n = 22), Mid-boreal Uplands (BCR 6, n= 28), Mid-boreal Lowland (BCR 6, n= 5), and the Boreal Transition (BCR 6, n= 8). We sampled ten out of 22 ecoregions within Yukon Territory, including sampling in the British-Richardson Mountains (BCR 3, n = 1), Old Crow Basin (BCR 4, n = 9), Old Crow Flats (BCR 4, n = 2), Eagle Plains (BCR 4, n = 3), North Ogilvie Mountains (BCR 4, n = 5), Mackenzie Mountains (BCR 4, n = 8), Klondike Plateau (BCR 4, n = 72), Yukon Plateau Central (BCR 4, n = 10), Yukon Plateau North (BCR 4, n = 10), and Ruby Ranges (BCR 4, n = 3).

Ethics statement

We obtained the required permits from provinces/territories, Provincial and Territorial Parks, Parks Canada Agency, and Yukon First Nations with Traditional Territories and Settlement Areas to conduct avian monitoring to test the BOSS design in

Newfoundland, Saskatchewan, and Yukon Territory. Field observers spend <15 minutes at SSU locations during observer surveys and <30 minutes (time for set up and subsequent retrieval) at SSU locations during Autonomous Recording Unit surveys resulting in minimal disturbance to breeding birds.

Statistical analyses

Prior to analysis of our multi-variate proxy data, we centered and standardized all variables to zero means and unit variance and then calculated Euclidean distances between all points (ecodistricts) in the dataset. We derived the multivariate estimate of dispersion by calculating mean distances to ecoregion centroids using the ‘betadisper’ function in the vegan package [51] in the R statistical computing environment [52]. The resulting estimates of dispersion for all ecodistricts within the boreal region as per Brandt [32] are available in Supporting Information S1.

In order to assess our BOSS design, we used the ‘spsurvey package’ [53] within the R statistical computing environment [52] to draw samples under four alternative spatially balanced sampling designs. Specifically, we drew samples under one equal-probability spatially balanced design and three unequal-probability designs: (a) a habitat stratification design, calculated as per equation (2) above; (b) a cost design in which inclusion probabilities were based solely on access costs as per equation (3); and (c) our BOSS design in which inclusion probabilities included both habitat stratification and access costs as per equation (4) above. For each scenario, we ran 100 iterations in which we drew a random sample of n= 400 PSUs from each of the three jurisdictions considered here. In each case, we drew a spatially balanced sample using the GRTS algorithm [53]. Within each iteration, our script calculated the access costs (CDN $),

spatial balance (Pielou evenness [28,53]), and habitat representation as SSD (see above). All implementations are available within scripts in the supplemental material (Supporting Information S2).

We used linear models to test for differences (for each jurisdiction separately) in each of our response variables (cost, SSD, and Pielou evenness) between the alternative designs described above (i.e. spatial, habitat, cost, and BOSS). Study design was included as a factor, and in each model we specified an *a priori* reference factor level to test our hypotheses. We specified the cost design as the reference factor level for models examining access costs, as we anticipated this design should have the lowest access costs on average. Similarly, we specified the habitat stratification design as the reference level for models examining variation in SSD as we predicted this design should have the lowest SSD.

All four designs we considered employed a GRTS algorithm to generate spatially balanced samples; however, the inclusion of unequal inclusion probabilities based on spatially structured variables could alter the ability of the design to achieve spatial balance. We therefore tested for differences in spatial balance between our designs and we treated the spatial design (equal-probability spatial sampling design) as the reference factor level when comparing spatial balance between sampling designs, but did not have *a priori* predictions. We calculated robust standard errors ('sandwich' estimators) to overcome problems with heteroscedastic errors using the 'lmtest' [54] and 'sandwich' [55] packages within the R statistical computing environment [52]. We applied a Bonferroni correction and considered results statistically significant when $\alpha \leq 0.05/9$.

Results

Sample Size Allocation

Sample size allocation to geographic strata (ecoregions) under the BOSS design resulted in a similar sample size on average as allocating samples based on stratum area as data roughly fall along a 1:1 correspondence line (Fig 4). However, allocation to individual strata differed markedly, with the BOSS design suggesting significantly low sample sizes for some strata than an area-based allocation while other strata were allocated larger sample size than would be assigned based on stratum area (Fig 4). For example, sampling proportional to area would allocate 151 PSUs to the New Québec Central Plateau ecoregion of Québec compared to only 50 based on the BOSS allocation because the stratum has lower than average dispersion (i.e. 0.36 vs average of 1.04). In contrast, 103 PSUs would be allocated to the Abitibi Plains ecoregion of Ontario under sampling proportional to stratum area, but 173 PSUs were allocated to the same stratum under the BOSS design owing to higher than average dispersion (i.e. 20.1 vs average of 1.04). Overall, the BOSS sample size allocation generally results in sampling intensities being greatest in the south and decreasing northward where (Fig 5).

Predicted sampling costs

Predicted costs to access 400 primary sample units varied by the jurisdiction and sampling design considered (Fig 6). Across all three jurisdictions, designs in which the inclusion probabilities derived solely based on access costs (cost design) were the least expensive designs (Fig 6) and all the alternative designs were statistically more

expensive regardless of jurisdiction (Table 1). The BOSS design had the next lowest cost (Fig 6), with costs predicted to be between 1.02 (Yukon Territory) and 1.2 (Saskatchewan) times more expensive on average than the cost design for a given jurisdiction (Table 1). In both Newfoundland and Saskatchewan the equal-probability spatial design had the third lowest cost followed by the habitat stratification design, whereas it was the most expensive design for Yukon Territory (Fig 6); it was predicted to be 1.4−2.6 times more expensive on average (~$362,000−562,000; Table 1) than the cost design (maximum of 1.5−3.0 times; Fig 6). The habitat stratification design was predicted to be 1.4−2.0 times more expensive on average (~$122,000−1,020,000; Table 1), or a maximum of 1.6−2.3 times more expensive across draws and jurisdictions (Fig 6).

Habitat representation

As predicted, the habitat stratification design had the lowest sum of squared differences between area represented within sample draws and the desired equal habitat representation (Fig 7). The BOSS design provided the second most representative sampling of habitat classes on average (Fig 7) with habitat representation in Saskatchewan and Yukon Territory showing no statistical difference, and Newfoundland having statistically lower habitat representation than the habitat design (Table 2). In contrast, both the equal-probability spatial design and the cost design had large (Fig 7) significantly lower habitat representation relative to the habitat stratification design in all three jurisdictions (Table 2).

Spatial representation

All sampling designs provided relatively balanced spatial sampling of all three jurisdictions (range of Pielou evenness across all designs: 0.96−1.01; Fig 8). Interestingly, the spatial balance of the cost design provided the closest approximation to the equal-probability spatial design (Fig 8) and was not statistically different compared to the equal-probability spatial design in either Newfoundland or Saskatchewan and small statistical differences in mean spatial balance in Yukon Territory (Table 3). The BOSS design showed small but statistically significant differences in spatial balance relative to a purely spatial design in both Newfoundland and Yukon Territory (Fig 8, Table 3), but not Saskatchewan (Table 3). The habitat stratification design had the largest overall differences in spatial balance relative to a pure spatial design, with small but statistically significant differences in all three jurisdictions and the largest difference occurring in Yukon Territory (Fig 8, Table 3).

Optimal design selection

The BOSS design provided the most optimal combination of cost savings and both spatial and habitat representation. Regardless of jurisdiction, the BOSS design had costs competitive with the cost design (Fig 6 and 9), with no loss of habitat or spatial representation. For all three jurisdictions combined, selecting the draw that simultaneously maximized representation while minimizing cost (see filled squares in Fig 9) only results in an estimated savings of approximately $2000 relative to the median of all sample draws. However, access costs for 400 PSUs varied substantially between draws (Newfoundland: $1,076,897−1,199,693; Saskatchewan: $1,089,811 −1,348,550; and Yukon Territory: $309,497−371,174) and thus relative to the most

expensive draws for each jurisdiction, the combined savings could be up to an estimated $225,000 for these three jurisdictions when using an optimal draw.

Field implementation

Of the 295 PSUs sampled across all three jurisdictions, we sampled 85 PSUs in remote areas that we predicted would require charter flights to access. In Newfoundland, cost models accurately predicted an average cost of $1,721 (SD = $86) per PSU to access 16 PSUs, compared to actual costs which averaged $1,721 (SD = $441) per PSU (Fig 8). In comparison, the cost models for Saskatchewan predicted an average access cost of $3,741 (SD= $1,928) for the 43 remote PSUs sampled, but the actual access costs were substantially lower (mean = $1,573, SD = $1,562). In Yukon, the mean predicted access cost to reach the 26 remote PSUs sampled was $1,574 (SD = $82; Fig 10) per PSU, but the mean cost to access these PSUs was $3,016 (SD = $132). Ordinary least squares regression suggests across all three jurisdictions the access costs were on average 48% of the model predicted cost on average (β = 0.48, SE = 0.10; Fig 10).

Discussion

We have integrated key concepts from sampling theory to develop a hierarchically structured survey design that can provide stratified sampling of predefined strata (e.g. ecoregions) and achieve the desired spatial balance and habitat representation while minimizing access costs. The probability-based BOSS design allows for valid statistical inference from field samples across the sampling frame and reduces operational costs. We found that when compared with equal-probability spatial designs (spatial design) or habitat-stratified unequal-probability spatial designs (habitat

stratification design), our integrated approach was significantly less expensive. Predicted access costs were 1.3−1.7 times more expensive for the habitat stratification design and 1.6−2.7 times more expensive on average for the spatial design compared to using our hierarchical BOSS design. Additionally, habitat representation in the BOSS design was statistically indistinguishable from the habitat stratification design for two out of three jurisdictions and was only marginally different for Newfoundland. While we did find minor statistical differences in spatial balance between the designs considered, spatial balance was always close to a value of one indicating the samples were well spread over jurisdictions irrespective of the type of design. In addition, the distribution of spatial balance metrics across iterations did not overlap one across all jurisdictions for the spatial design, and thus it does not appear that the inclusion of unequal sampling probabilities introduced any systematic biases in spatial representation. Combined with our optimization, we therefore simultaneously achieved habitat and spatial representation goals while minimizing access costs.

Our pilot field seasons suggest that logistic considerations might result in access costs differing from those suggested by our cost models. While cost models for Newfoundland have thus far provided accurate prediction of average access costs per PSU, our cost models slightly underestimated access costs in Yukon. Overall, regression analysis suggested that field implementation was 52% cheaper than suggested by our cost models. Many reasons likely contributed to the cost differences we observed, including external logistical support. One key difference is that our cost models largely portray cost of access to a sample unit as independent of access to other nearby sampling units. In practice, we were able to combine logistics and access

methods for many sampling units and thereby substantially lowered the average cost per sample unit. For example, in the summer of 2019 we chartered a Twin Otter to drop off two crews on or near opposite sides of Cree Lake, Saskatchewan (57°23′57″N 106°40′10″W) to access six PSUs via canoe. When the plane returned to retrieve crews, an additional crew was dropped off to canoe down a river back to a roadside pickup location, allowing access to two additional PSUs. We were therefore able to access eight PSUs for the cost of two charter flights, substantially reducing the average cost per PSU. We therefore recommend that programs revise their cost models as field implementation improves knowledge of local logistics. As a result, final program costs will likely be lower than initially predicted by preliminary cost models,

Our sampling design has several key differences from other large-scale terrestrial bird monitoring programs that improve its' utility for remote areas such as the boreal forest. Other programs such as the Integrated Monitoring in Bird Conservation Regions (IMBCR) program [9], the UK Breeding Bird Survey [56], the North American BBS [12] and the US National Park Service Vital Signs Monitoring program [57] also have defined sampling frames and randomly selected sample units that facilitate valid statistical inference from field samples [58]. Importantly, our design has fewer restrictions on the sampling frame while minimizing sampling costs, and implicitly accommodates health and safety considerations. While other programs restrict the sampling frame to roadsides or trail networks to facilitate access and alleviate safety concerns, we instead dealt with access by incorporating access costs into sample unit selection, and designed primary and secondary sampling units to have first responders available if needed. Thus, our design increases safety and reduces costs without

limiting statistical inference about bird populations to accessible areas [58,59,60]. In addition, our hierarchical stratification with static strata (BCRs, jurisdictions and ecoregions) allows for assessment of the population-level responses of birds to natural and human disturbance and climate change [9,61]. Indeed, Pavlacky et al. [9] recently demonstrated the ability of their hierarchical spatial design to allow estimation of both strata-specific population sizes and species-habitat relationships. Our approach of integrating both cost and weighted habitat-inclusion probabilities within a similar spatially balanced design will allow us to draw similar inferences to Pavlacky et al. [9] at multiple spatial scales, but at reduced costs compared to alternative approaches. The ability to model species-habitat relationships is a key feature of the BOSS design, which will greatly improve our capacity to develop and test models of species response to habitat, which are crucial to predicting responses to industrial development [15,16,62] and climate change [18].

Our design directly incorporated several factors into the stratification and layout of primary and secondary sampling units to address the need to minimize costs and provide for the health and safety of field staff. Field implementation of the optimal designs selected here during pilot field seasons across all three jurisdictions showed that the BOSS design was achievable on the ground. We were able to apply the design across a wide variety of challenging conditions from mountainous terrain (e.g. >2000 m), wetlands (floating bogs, fens and marshes), forests with blow-down (recent burns, tornados and insect outbreaks), as well as steep, rocky, and densely vegetated slopes across all three jurisdictions. Furthermore, drawing an oversample using the GRTS algorithm allowed us to replace unsafe PSUs without altering the representativeness of

the sample [25]. Using pairs of observers to conduct surveys within each PSU means that staff can work alone to each survey a plot of nine SSU within a PSU or they can work together to complete one plot of nine SSU before travelling together to complete the second plot. The latter option may be appropriate if a PSU contains dangerous terrain or wildlife or limits communication between staff (radio, satellite transmitter).

One complication imposed by our design is that most analyses assume that sampling is proportional to habitat availability and, by extension, population sizes. We used habitat stratification to ensure representation across the range of available habitat classes and thereby provide reasonable sample sizes to facilitate modeling abundance and distributions of rare species often associated with rare habitats. Habitat stratification should also improve our future capacity to detect trends of species using rare habits. As a result, extrapolating from the sample to estimate stratum-specific population sizes and/or population trends is more complicated because strata boundaries and sizes can change with shifts in vegetation through time [10]. This problem can be addressed analytically via post-hoc stratification [63,64], weighting via the inclusion probabilities [65,66], including random-effects for the strata indexed by the inclusion probabilities [67], or inclusion of interval-specific covariates reflecting habitat supply [66].

While our BOSS design presented here incorporates several key improvements over current large-scale bird monitoring designs, we foresee several areas where further continued improvements could occur. First, we stratified habitat using coarse satellite-derived products due to their availability and consistency across the entire sampling frame. Incorporating higher-resolution products capable of further stratifying habitat classes, and potentially better quantifying the amount and distribution of rare

habitats, should improve our capacity to monitor species associated with these habitats. One possible approach worth investigation would be to create an avian habitat classification (*sensu* [42]) from higher-resolution data, such as forest inventory data, for the portion of the sample frame for which they may be available. Higher-resolution data could be integrated into the current framework by (1) using the coarse-resolution satellite data to select PSUs and using the high-resolution habitat classification to stratify SSUs within the selected PSUs, or (2) splitting the selection of PSUs between areas with versus without access to these high-resolution data products.

Second, once sufficient on-the-ground data are available, it would be desirable to re-examine the stratification by using the design based sample to directly estimate spatial and temporal variance in bird community composition for Neyman allocation rather than the proxy variables used in the initial sample. Regional differences in timing of implementation, available resources, and historic sampling efforts mean that this data will be available earlier in some regions than others. We anticipate that sampling efficiency will improve by incorporating these direct estimates of variability into the design, but overall costs could be inflated if these data are not incorporated until they are available for all regions. Using data from preliminary rounds of sampling under the sampling design to validate Neyman allocation based on multivariate dispersion of proxy variables will ensure allocation between strata is cost effective.

To avoid inefficiencies, there is a clear need for improvements in cost models or habitat layers to be reflected in revised sample draws; however, revised sample draws should account for existing sampling under the BOSS design to avoid inducing spatial imbalance. Foster et al. [26] recently proposed using a squared loss distance metric to

alter the inclusion probabilities around “legacy” monitoring sites (i.e. pre-existing sampling locations are known to be a randomly selected representative sample from a known sample frame [26]). This approach would provide a clear way to improve future sampling under the BOSS design without introducing biases and inefficiencies due to spatial imbalances. In addition to data from legacy sites, a large collection of historic data exist within the Boreal Avian Modeling (*hereafter* BAM) project database [68] for a large portion of boreal Canada. The difficulty with incorporating data from the BAM database is that these largely represent “iconic sites” (*sensu* [26]) which are either known to be non-randomly selected and/or there is insufficient documentation to know whether the sampling frame included the full suite of habitat classes. Including iconic sites in a randomized draw could introduce bias into the design and result in biased status and trend estimates that may not match those in the population [26]. Since these historic datasets could potentially add valuable ecological insights into factors affecting populations of boreal birds, it would be fruitful to consider when and how they could be included within a long-term monitoring design.

We have demonstrated that including access costs and habitat within a hierarchically structured sampling scheme was the most cost-efficient design that maintained the desired spatial and habitat representation. Although we have focused on a design for monitoring boreal birds, our approach should be broadly applicable and adaptable as a template for other regions where remote locations form a large proportion of the sampling frame. We recommend that our approach be adapted for use in other large-scale studies such as Breeding Bird Atlases [69] if access and sampling costs are limiting for much if not all of the study areas. For example, the Saskatchewan

Breeding Bird Atlas (https://sk.birdatlas.ca/) is currently using systematic sampling within accessible areas, and the BOSS design within the boreal portion of the province. To facilitate adaptation and implementation of our approach and/or further refinements to our method, we provide worked examples and scripts within the Supporting Information. Using design-based approaches to inform the design of large-scale studies would add to their statistical rigor and efficiency and facilitate their integration (i.e. treated as legacy sites) into long-term monitoring to provide more responsive conservation efforts in light of widespread declines in biodiversity [1-3]. Our hierarchical sampling design should be widely implemented to monitor boreal birds because it can provide an unbiased representation of when and where conservation and management should be targeted [9,70]. Using monitoring data to inform conservation decisions is crucial, given ongoing and projected ecological changes in the boreal biome [18,34] and population declines already observed for boreal birds [3].

Acknowledgments

This research was supported by Environment and Climate Change Canada. We are extremely grateful to R. Weeber for discussions leading to several of the concepts incorporated within this design. We thank M. Robertson and D. Hope for helpful comments on an earlier draft of this manuscript.

Author Contributions

Conceptualization: Van Wilgenburg, Mahon, G. Campbell, McLeod, M. Campbell, Evans, Drolet, Easton, Francis, Hache, Machtans, Russell, Smith, Thomas, Toms, Tremblay

Data curation: Van Wilgenburg, G. Campbell, McLeod

Formal analysis: Van Wilgenburg, G. Campbell, McLeod

Methodology: Van Wilgenburg, Mahon, G. Campbell, McLeod

Writing, review and editing: Van Wilgenburg, Mahon, G. Campbell, McLeod, M. Campbell, Evans, Drolet, Easton, Francis, Hache, Machtans, Russell, Smith, Thomas, Toms, Tremblay

References

1. Pimm SL, Jenkins CN, Abell R, Brooks TM, Gittleman JL, Joppa LN, et al. The biodiversity of species and their rates of extinction, distribution and protection. Science. 2014 May 30; 344(6187): p. 1246752.

2. Butchart SM, Walpole M, Collen B, van Strien A, Scharlemann JP, Almond RE, et al. Global biodiversity: indicators of recent declines. Science. 2010 May 28; 328(5982): p. 1164-1168.

3. Rosenberg KV, Dokter AM, Blancher PJ, Sauer JR, Smith AC, Smith PA, et al. Decline of the North American avifauna. Science. 2019 September 19; 365(6459): p. eaaw1313.

4. Sala OE, Chapin III FS, Armesto JJ, Berlow E, Bloomfield J, Dirzo R, et al. Global Biodiversity Scenarios for the Year 2100. Science. 2000 March 10; 287(5459): p. 1771-1774.

5. Bellard C, Bertelsmeier C, Leadley P, Thuiller W, Courchamp F. Impacts of climate change on the future of biodiversity. Ecology Letters. 2012 April; 15(4): p. 365-377.

6. Taylor MF, Suckling KF, Rachlinski JJ. The effectiveness of the Endangered Species Act: a quantitative analysis. BioScience. 2005; 4(1): p. 360-367.

7. Kerr JT, Cihlar J. Patterns and causes of species endangerment in Canada. Ecological Applications. 2004 June 1; 14(3): p. 743-753.

8. Rice J. Environmental health indicators. Ocean & Coastal Managment. 2003; 46(3-4): p. 235-259.

9. Pavlacky Jr DC, Lukacs PM, Blakesley JA, Skorkowsky RC, Klute DS, Hahn BA, et al. A statistically rigorous sampling design to integrate avian monitoring and management within Bird Conservation Regions. PLoS ONE. 2017; 12(10): p. e0185924.

10. Gitzen RA, Millspaugh JJ. Ecological monitoring: the heart of the matter. In Gitzen RA, Millspaugh JJ, Cooper AB, Licht DS, editors. Design and analysis of long-term ecological monitoring studies. New York: Cambridge University Press; 2012. p. 3-22.

11. Nichols JD, Williams BD. Monitoring for conservation. Trends in Ecology & Evolution. 2006 December; 21(12): p. 668-673.

12. Hudson MAR, Francis CM, Campbell KJ, Downes CM, Smith AC, Pardieck KL. The role of the North American Breeding Bird Survey in conservation. The Condor. 2017; 119(3): p. 526-545.

13. Machtans CS, Kardynal KJ, Smith PA. How well do regional or national Breeding Bird Survey data predict songbird population trends at an intact boreal site. Avian Conservation and Ecology. 2014; 9(1): p. 5.

14. Bart J, Burnham KP, Dunn EH, Francis CM, Ralph CJ. Goals and strategies for estimating trends in landbird abundance. Journal of Wildlife Management. 2004; 68(3): p. 611-626.

15. Mahon CL, Bayne EM, Sólymos , Matsuoka SM, Carlson M, Dzus E, et al. Does expected future landscape condition support proposed population objectives for boreal birds? Forest Ecology and Management. 2014 January 15; 312.

16. Mahon CL, Holloway GL, Bayne EM, Toms JD. Additive and interactive cumulative effects on boreal landbirds: winners and losers in a multi-stressor landscape. Ecological Applications. 2019 July; 29(5): p. e01895.

17. Watson JE, Evans T, Venter O, Williams B, Tulloch A, Stewart C, et al. The exceptional value of intact forest ecosystems. Nature ecology & evolution. 2018 February 26; 2: p. 599–610.

18. Stralberg D, Bayne EM, Cumming SG, Sólymos P, Song SJ, Schmiegelow FK. Conservation of future boreal forest bird communities considering lags in vegetation response to climate change: a modified refugia approach. Diversity and Distributions. 2015 July 20; 21(9): p. 1112-1128.

19. SARA (Species at Risk Act). Bill C-5, an Act respecting the protection of wildlife Species at Risk in Canada. 2002..

20. Westwood A, Olszybski M, Fox CH, Ford AT, Jacob AL, Moore JW, et al. The role of science in contemporary Canadian environmental decision making: the example of environmental assessment. University of British Columbia Law Review. 2019 February; 52(1): p. 243-292.

21. COSEWIC (Committe on the Status of Endangered Wildlife in Canada). COSEWIC's assessment process and criteria. 2010..

22. Van Wilgenburg SL, Beck EM, Obermayer B, Joyce T, Weddle B. Biased representation of disturbance rates in the roadside sampling frame in boreal forests: implications for monitoring design. Avian Conservation and Ecology. 2015 December; 10(2): p. 5.

23. Tulloch AI, Mustin K, Possingham HP, Szabo JK, Wilson KA. To boldly go where no volunteer has gone before: predicting volunteer activity to prioritize surveys at the landscape scale. Diversity and Distributions. 2013 April 03; 19(4): p. 465-480.

24. Buckland ST, Marsden SJ, Green RE. Estimating bird abundance: making methods work. Bird Conservation International. 2008; 18(s1): p. S91-S108.

25. Stevens Jr DL, Olsen AR. Spatially-balanced sampling of natural resources. Journal of the American Statistical Association. 2004; 99(465): p. 262-278.

26. Foster SD, Hosack GR, Lawrence E, Przeslawski R, Hedge P, Caley MJ, et al. Spatially balanced designs that incorporate legacy sites. Methods in Ecology and Evolution. 2017; 8(11): p. 1433-1442.

27. Neyman J. On the two different aspects of the representative method: the method of stratified sampling and the method of purposive selection. Journal of the Royal Statistical Society. 1934; 97(4): p. 558–625.

28. Olsen AR, Kincaid TM, Payton Q. Spatially balanced survey designs for natural resources. In Gitzen RA, Millspaugh JJ, Cooper AB, Licht DS, editors. Design and analysis of long-term ecological monitoring studies. New York: Cambridge University Press; 2012. p. 126-150.

29. Clark RG. Sample design using imperfect design data. Journal of Survey Statistics and Methodology. 2013; 1(1): p. 6-23.

30. Van Wilgenburg SL, Hobson KA, Kardynal KJ, Beck EM. Temporal changes in avian abundance in aspen-dominated boreal mixedwood forests of central Saskatchewan, Canada. Avian Conservation and Ecology. 2018 June; 13(1): p. 3.

31. Stehman SV, Overton WS. Environmental sampling and monitoring Patil GP, Rao CR, editors. Amsterdam: Elsevier Science; 1994.

32. Brandt JP. The extent of the North American boreal zone. Environmental Reviews. 2009; 17: p. 101-161.

33. Synes NW, Brown C, Watts K, White SM, Gilbert MA, Travis JMJ. Emerging opportunities for landscape ecological modelling. Current Landscape Ecology Reports. 2016; 1(4): p. 146-167.

34. Langham GM, Schuetz JG, Distler T, Soykan CU, Wilsey C. Conservation status of North American birds in the face of future climate change. PLoS ONE. 2015 September; 10(9): p. e0135350.

35. Thompson WL, White GC, Gowan C. Monitoring vertebrate populations San Diego: Academic Press; 1998.

36. Government of Canada. Ecological Land Classification, 2017. Ottawa:, Statistics Canada; 2018. Report No.: 978-0-660-24501-0.

37. Cochran WG. Sampling techniques. 3rd ed. Toronto: John Wiley & Sons; 1977.

38. GeoInsight Corporation. An approach to assessing risk to terrestrial biodiversity in Canada. State of the Environment Reporting Occasional Paper Series No. 11. Ottawa:, Environment Canada; 1999.

39. Anderson MJ, Ellingsen KE, McArdle BH. Multivariate dispersion as a measure of beta diversity. Ecology Letters. 2006; 9(6): p. 683-693.

40. Anderson MJ. Distance-based tests for homogeneity of multivariate dispersions. Biometrics. 2006; 62(1): p. 245-253.

41. Cumming SG, Stralberg D, Lefevre KL, Sólymos P, Bayne EM, Fang S, et al. Climate and vegetation hierarchically structure patterns of songbird distribution in the Canadian boreal region. Ecography. 2014 February; 37(2): p. 137-151.

42. Mahon CL, Holloway G, Sólymos P, Cumming SG, Bayne EM, Schmiegelow F. Community structure and niche characteristics of upland and lowland western boreal birds at multiple spatial scales. Forest Ecology and Management. 2016 February; 361: p. 99-116.

43. Hobson KA, Bayne E. The effects of stand age on avian communities in aspen-dominated forests of central Saskatchewan, Canada. Forest Ecology and Management. 2000; 136(1-3): p. 121-134.

44. Latifovic R, Homer C, Ressl R, Pouliot D, Hossain S, Colditz R, et al. North American land-change monitoring system. In Giri CP, editor. Remote Sensing of Land Use and Land Cover: Principles and Applications. Boca Raton: CRC/Taylor & Francis; 2012. p. 303-343.

45. Morissette JL, Kardynal KJ, Bayne EM, Hobson KA. Are boreal riparian bird communities unique? Contrasting riparian and upland bird assemblages in the Boreal Plain of western Canada. Wetlands. 2018 December; 38(6): p. 1299-1311.

46. Harris I, Jones PD, Osborn TJ, Lister DH. Updated high-resolution grids of monthly climatic observations – the CRU TS3.10 Dataset. International Journal of Climatology. 2014; 34(3): p. 623-642.

47. Parisien MA, Parks SA, Krawchuk MA, Little JM, Flannigan MD, Gowman LM, et al. An analysis of controls on fire activity in boreal Canada: comparing models built with different temporal resolutions. Ecological Applications. 2014; 24(6): p. 1341-1356.

48. Matsuoka SM, Mahon CL, Handel CM, Sólymos P, Bayne EM, Fontaine PC, et al. Reviving common standards in point-count surveys for broad inference across studies. The Condor: Ornithological Applications. 2014; 116(4): p. 599-608.

49. Shonfield J, Bayne EM. Autonomous recording units in avian ecological research: current use and future applications. Avian Conservation and Ecology. 2017; 12(1): p. 14.

50. Kalton G, Anderson DW. Sampling rare populations. Journal of the Royal Statistical Society: Series A. 1986; 149(1): p. 65-82.

51. Oksanen J, Blanchet FG, Kindt R, Legendre P, Minchin PR, O'Hara RB, et al. Vegan: community ecology package. R Package 2.3-3. 2016..

52. R Core Team. R: A language and environment for statistical computing. Version 3.3.1. R Foundation for Statistical Computing, Vienna, Austria. 2016..

53. Kincaid TM, Olsen AR. spsurvey: spatial survey design and analysis. R package version 4.0.0. 2019..

54. Zeileis A, Hothorn T. Diagnostic checking in regression relationships. R News. 2002; 2(3): p. 7-10.

55. Zeileis A. Object-oriented computation of sandwich estimators. Journal of Statistical Software. 2006; 16: p. 1-16.

56. Freeman SN, Nobble DG, Newson SE, Baillie SR. Modelling population changes using data from different surveys: the Common Birds Census and the Breeding Bird Survey. Bird Study. 2007; 54(1): p. 61-72.

57. Fancy SG, Gross JE, Carter SL. Monitoring the condition of natural resources in US national parks. Environmental Monitoring and Assessment. 2009; 151(1-4): p. 161-174.

58. Smith AN, Anderson MJ, Pawley MD. Could ecologists be more random? Straightforward alternatives to haphazard spatial sampling. Ecography. 2017 November; 40: p. 1251-1255.

59. Siegel RB, Wilkerson RL, The Institute for Bird Populations , Jenkins KJ, U.S. Geological Survey , Kuntz II RC, et al. Landbird monitoring protocol for National Parks in the North Coast and Cascades Network. Techniques and Methods 2-A6. U.S. Geological Survey, U.S. Department of the Interior; 2007.

60. Weber S, Woodward A, Freilich J. North Coast and Cascades Network Vital Signs Monitoring Report. Natural Resource Report NPS/NCCN/NRR—2009/098. Fort Collins: National Park Service, U.S. Department of the Interior; 2005.

61. Metzger MJ, Brus DJ, Bunce RGH, Carey PD, Gonçalves J, Honrado JP, et al. Environmental stratifications as the basis for national, European and global ecological monitoring. Ecological Indicators. 2013 October; 33: p. 26-35.

62. Roy C, Michel NL, Handel CM, Van Wilgenburg SL, Burkhalter JC, Gurney KEB, et al. Monitoring boreal avian populations: how can we estimate trends and trajectories from noisy data? Avian Conservation and Ecology. 2019; 14(2): p. 8.

63. Nahorniak M, Larsen DP, Volk C, Jordan CE. Using inverse probability bootstrap sampling to eliminate sample induced bias in model based analysis of unequal probability samples. PLoS ONE. ; 10(6): p. e0131765.

64. van Turnhout CAM, Willems F, Plate C, van Strien A, Teunissen W, van Dijk A, et al. Monitoring common and scarce breeding birds in the Netherlands: applying a post-hoc stratification and weighting procedure to obtain less biased population trends. Revista Catalana d'Ornitologia. 2008; 24: p. 15-29.

65. Lumley T. Analysis of complex survey samples. Journal of statistical software. 2004; 9(1): p. 1-19.

66. Pfeffermann D, Skinner CJ, Holmes DJ, Goldstein H, Rasbash J. Weighting for unequal selection probabilities in multilevel models. Journal of the Royal Statistical Society: series B (statistical methodology). 1998; 60(1): p. 23-40.

67. Little RJ. Estimating a finite population mean from unequal probability samples. Journal of the American Statistical Association. 1983; 78(383): p. 596-604.

68. Barker NK, Fontaine PC, Cumming SG, Stralberg D, Westwood A, Bayne EM, et al. Ecological monitoring through harmonizing existing data: Lessons from the boreal avian modelling project. Wildlife Society Bulletin. 2015; 39(3): p. 480-487.

69. McCabe JD, Anich NM, Brady RS, Zuckerberg B. Raising the bar for the next generation of biological atlases: using existing data to inform the design and implementation of atlas monitoring. Ibis. 2018 July; 160(3): p. 528-541.

70. Wilson KA, Underwood EC, Morrison SA, Klausmeyer KR, Murdoch WW, Reyers B, et al. Conserving biodiversity efficiently: what to do, where, and when. PLOS Biology. 2007 August 21; 5(9): p. e223.

71. Handel CM, Sauer JR. Alaska, analysis of roadside and off-road breeding bird survey data to assess population change in Alaska. 2017; 119(3): p. 557-575.

72. DeWan AA, Zipkin EF. An integrated sampling and analysis approach for improved biodiversity monitoring. Environmental Management. 2010; 45(5): p. 1223-1230.

73. Lindenmayer DB, Likens GE. Effective ecological monitoring Collingwood: CSIRO Publishing; 2010.

74. Hewitt JE, Thrush SF, Dayton PK, Bonsdorff E. The effect of spatial and temporal heterogeneity on the design and analysis of empirical studies of scale-dependent systems. The American Naturalist. 2007 March; 169(3): p. 398-408.

75. Lindenmayer DB, Likens GE. The science and application of ecological monitoring. Biological Conservation. 2010; 143(6): p. 1317–1328.

76. Héon J, Arseneault D, Parisien MA. Resistance of the boreal forest to high burn rates. Proceedings of the National Academy of Sciences. 2014; 111(38): p. 3888-13893.

77. Bart J. Monitoring the abundance of bird populations. Auk. 2005; 122(1): p. 15-25.

78. Urquhart NS, Kincaid TM. Designs for detecting trend from repeated surveys of ecological resources. Journal of Agricultural, Biological, and Environmental Statistics. 1999; 4(4): p. 404-414.

79. Sólymos P, Toms JD, Matsuoka SM, Cumming SG, Barker NKS, Thogmartin WE, et al. At the end of the road: Lessons learned from comparing model- and design-based. The Condor. in review;: p. XXX-XXX.

80. Stadt JJ, Schieck J, Stelfox HA. Alberta Biodiversity Monitoring Program–monitoring effectiveness of sustainable forest management planning. Environmental Monitoring and Assessment. 2006 September; 121(1-3): p. 33-46.

Table 1. Linear model results comparing variation in access costs ($) between alternative monitoring designs. The cost design was the reference category (Intercept). Bolded values are statistically “significant” after Bonferroni correction.

Design	β	SE	t-value	p
Newfoundland				
Intercept	1,006,006.0	2870.2	350.51	**<0.001**
BOSS design	121,535.7	3951.3	30.76	**<0.001**
Spatial design	362,012.3	4072.3	88.90	**<0.001**
Habitat Design	482,229.8	4023.7	119.85	**<0.001**
Saskatchewan				
Intercept	1,053,370.5	4927.6	213.77	**<0.001**
BOSS design	175,634.7	7288.8	24.10	**<0.001**
Spatial design	561,151.8	7587.2	73.96	**<0.001**
Habitat Design	1,020,042.6	8439.4	120.87	**<0.001**
Yukon				
Intercept	331,627.1	990.8	334.70	**<0.001**
BOSS design	8700.2	1615.1	5.39	**<0.001**
Spatial design	541,671.3	1658.7	326.57	**<0.001**
Habitat Design	121,812.7	1507.5	80.80	**<0.001**

Table 2. Linear model results comparing variation in habitat representation (i.e. sum of squared differences between area of land cover classes represented within sample draws against a balanced sample (see Methods)) between alternative sampling designs. The habitat design was the reference category (Intercept). Bolded values are statistically “significant” after Bonferroni correction.

Design	β	SE	t-value	p
Newfoundland				
Intercept	0.06	3.84×10^{-4}	158.59	**<0.001**
BOSS design	4.90×10^{-3}	5.60×10^{-4}	8.75	**<0.001**
Spatial design	0.11	9.97×10^{-4}	108.35	**<0.001**
Cost Design	0.10	8.97×10^{-4}	114.37	**<0.001**
Saskatchewan				
Intercept	0.04	2.06×10^{-4}	215.01	**<0.001**
BOSS design	9.36×10^{-4}	3.10×10^{-4}	3.02	0.003
Spatial design	0.01	4.02×10^{-4}	32.31	**<0.001**
Cost Design	0.02	3.93×10^{-4}	41.56	**<0.001**
Yukon				
Intercept	0.10	4.78×10^{-4}	211.13	**<0.001**
BOSS design	1.53×10^{-3}	7.17×10^{-4}	2.14	0.03
Spatial design	0.04	6.47×10^{-4}	61.51	**<0.001**
Cost Design	0.04	8.40×10^{-4}	53.36	**<0.001**

Table 3. Linear model results examining variation in spatial balance (Pielou eveness) between alternative sampling designs. The equal probability spatially balanced sampling design as the reference category (Intercept). Bolded values are statistically “significant” after Bonferroni correction.

Design	β	SE	t-value	p
Newfoundland				
Intercept	0.99	6.52×10^{-4}	1514.78	**<0.001**
BOSS design	8.70×10^{-3}	8.80×10^{-4}	9.87	**<0.001**
Cost design	1.56×10^{-3}	9.03×10^{-4}	1.72	0.09
Habitat Design	4.18×10^{-3}	8.92×10^{-4}	4.68	**<0.001**
Saskatchewan				
Intercept	0.99	3.32×10^{-4}	2977.07	**<0.001**
BOSS design	-1.10×10^{-3}	4.23×10^{-4}	-2.61	0.009
Cost design	9.22×10^{-4}	4.52×10^{-4}	2.04	0.04
Habitat Design	-1.39×10^{-2}	4.41×10^{-4}	-31.61	**<0.001**
Yukon				
Intercept	1.00	3.12×10^{-4}	3204.27	**<0.001**
BOSS design	-1.59×10^{-2}	5.81×10^{-4}	27.41	**<0.001**
Cost design	-6.15×10^{-3}	5.69×10^{-4}	-10.82	**<0.001**
Habitat Design	-1.21×10^{-2}	5.89×10^{-4}	-20.68	**<0.001**

Figure Headings

Fig 1. Spatial extent of the sampling frame of the Boreal Optimal Sampling Strategy (BOSS) design. The green region represents the boundary of the boreal region as defined in [32] with a 100 km northern buffer within the 4-Northwestern Interior Forest, 6-Boreal Taiga Plains, 7-Taiga Shield and Hudson Plains, and 8-Boreal Softwood Shield Bird Conservation Regions.

Fig 2. The two-stage sampling design of the Boreal Optimal Sampling Strategy (BOSS) design. This figure illustrates the selection of Primary Sampling Units (PSU, 5 km diameter hexagonal grid cell) from ecoregions within the sampling frame and selection of Secondary Sampling Units (SSU, point count survey locations) within the selected PSUs.

Fig 3. Components of the Boreal Optimal Sampling Strategy (BOSS) design depicted for Newfoundland and Labrador, Saskatchewan, and Yukon Territory: (a) access cost; (b) weighted habitat inclusion probability; (c) cost and weighted habitat inclusion probability.

Fig 4. Allocation of sampling effort between boreal ecoregions of Canada under the Boreal Optimal Sampling Strategy (BOSS; see Methods) versus traditional allocation

based on Stratum areas to achieve a total sample size of 4980 Primary Sample Units (i.e. 2% sample). Dashed line indicates the 1:1 correspondence line.

Fig 5. Geographic distribution of sampling intensity (number of allocated primary sampling units per 1000 km^2) across the boreal ecoregions of Canada under the Boreal Optimal Sampling Strategy (BOSS) design (see Methods).

Fig 6. Variation in predicted costs (in Canadian dollars x 10^6) of accessing 400 primary sampling units in each of Newfoundland and Labrador, Saskatchewan and Yukon Territory. Data are from 100 separate random draws of 400 primary sampling units in each jurisdiction. Note differing y-axis scales due to variation in overall costs of operating in each jurisdiction.

Fig 7. Variation in habitat class representation (measured as the sum of square differences between the area of each habitat class within all PSUs in a randomized draw versus the proportional area of each habitat class, see Materials and Methods) between alternative sampling designs in Newfoundland and Labrador, Saskatchewan and Yukon Territory. Data are from 100 separate random draws of 400 primary sampling units in each jurisdiction.

Fig 8. Variation in spatial balance (Pielou evenness) between alternative sampling designs in Newfoundland, Saskatchewan and Yukon Territory. Data are from 100 separate random draws of 400 primary sampling units in each jurisdiction.

Fig 9. Between draw variation in combined spatial and habitat (weighted sum, see Methods) representativeness of sampling relative to access costs in Newfoundland and Labrador, Saskatchewan and Yukon Territory for four alternative sampling designs: cost based sampling design (open red triangles), the BOSS design (open black squares), the habitat design (open green triangles), and an equal probability spatial sampling design (blue filled triangles). Red filled square within the cluster of points representing the BOSS design represents the lowest cost solution that maximizes combined spatial and habitat representation. Note differing x-axis scales due to variation in overall costs of operating in each jurisdiction.

Fig 10. Predicted versus realized access costs (in Canadian dollars) for each of Newfoundland and Labrador, Saskatchewan and Yukon Territory. Solid line represents ordinary least squares fit and dashed lines represent 95% confidence intervals.

Figure 1

Click here to download Figure Fig 1.tif

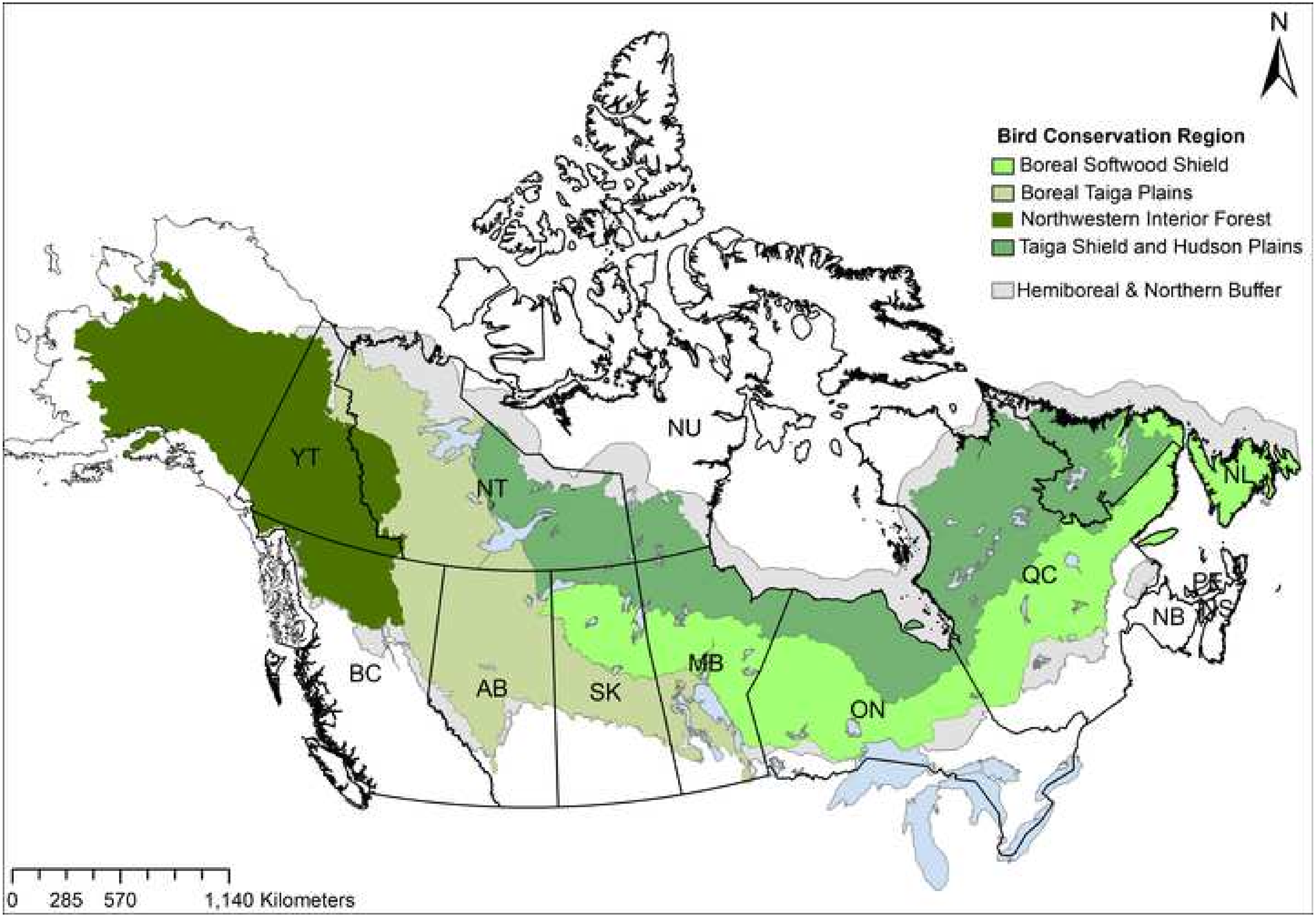

Figure 2

Click here to download Figure Fig 2.tif

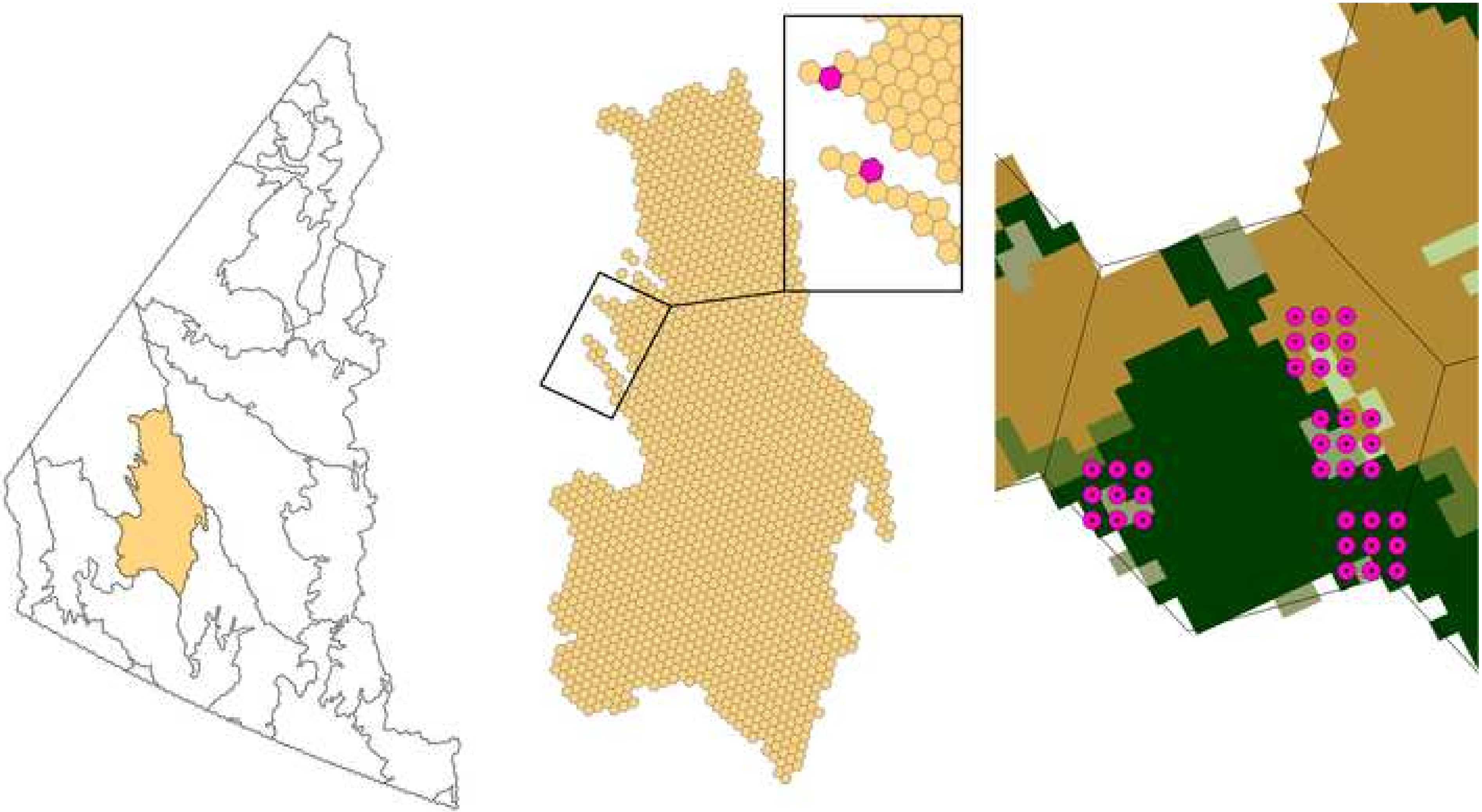

Figure 3

Click here to download Figure Fig 3.tif

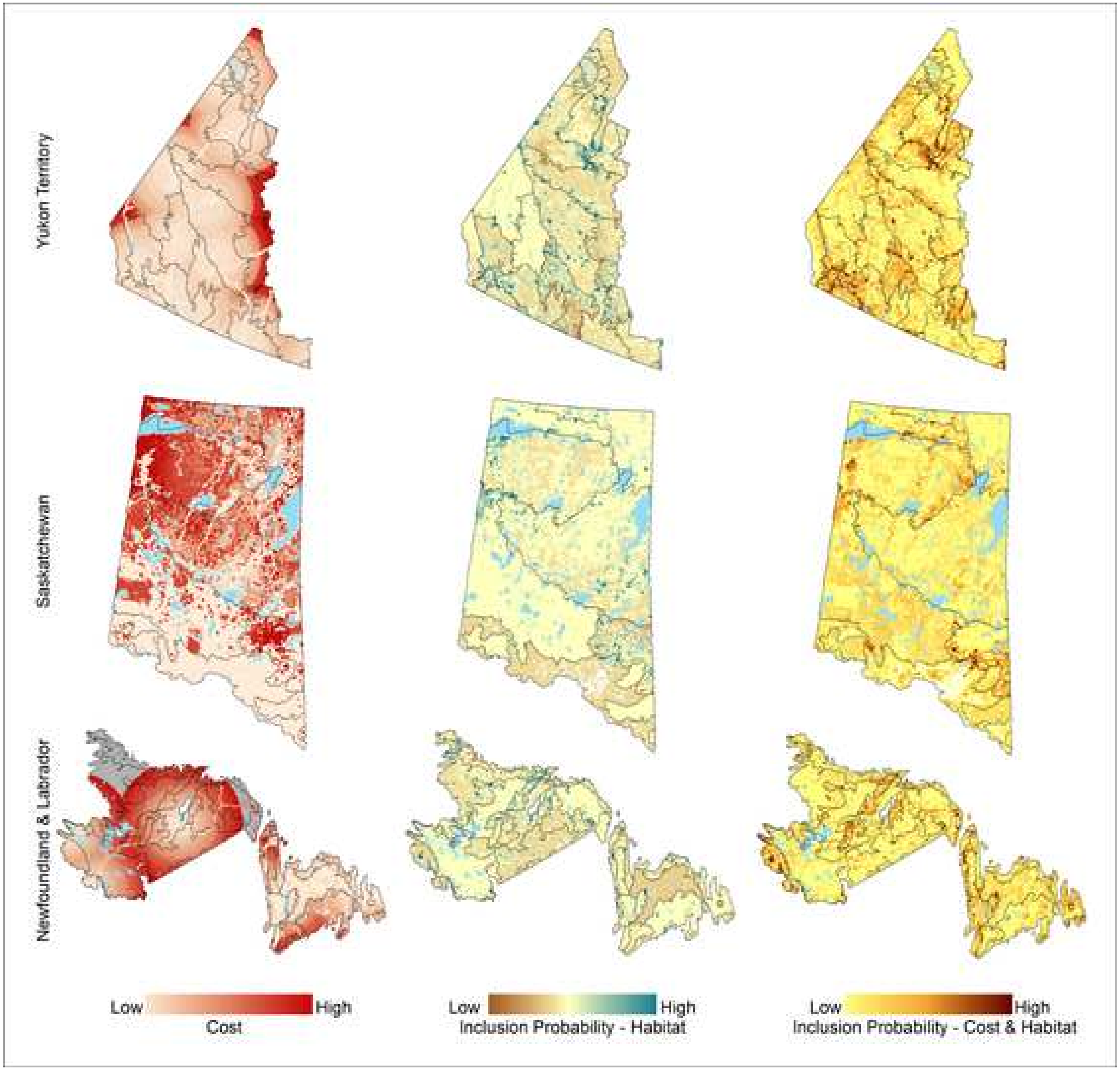

Figure 4

Click here to download Figure Fig 4.tif

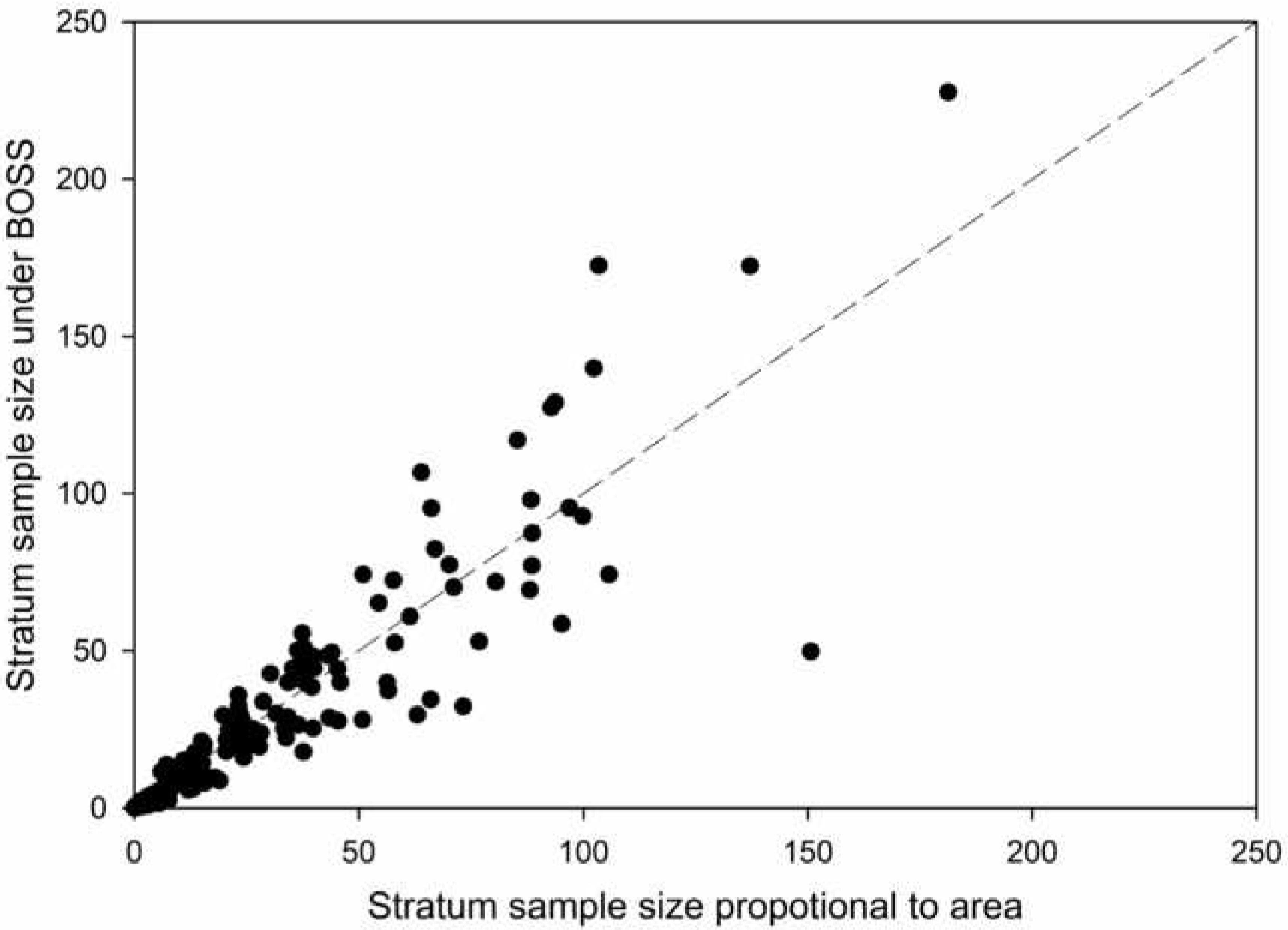

Figure 5

Click here to download Figure Fig 5.tif

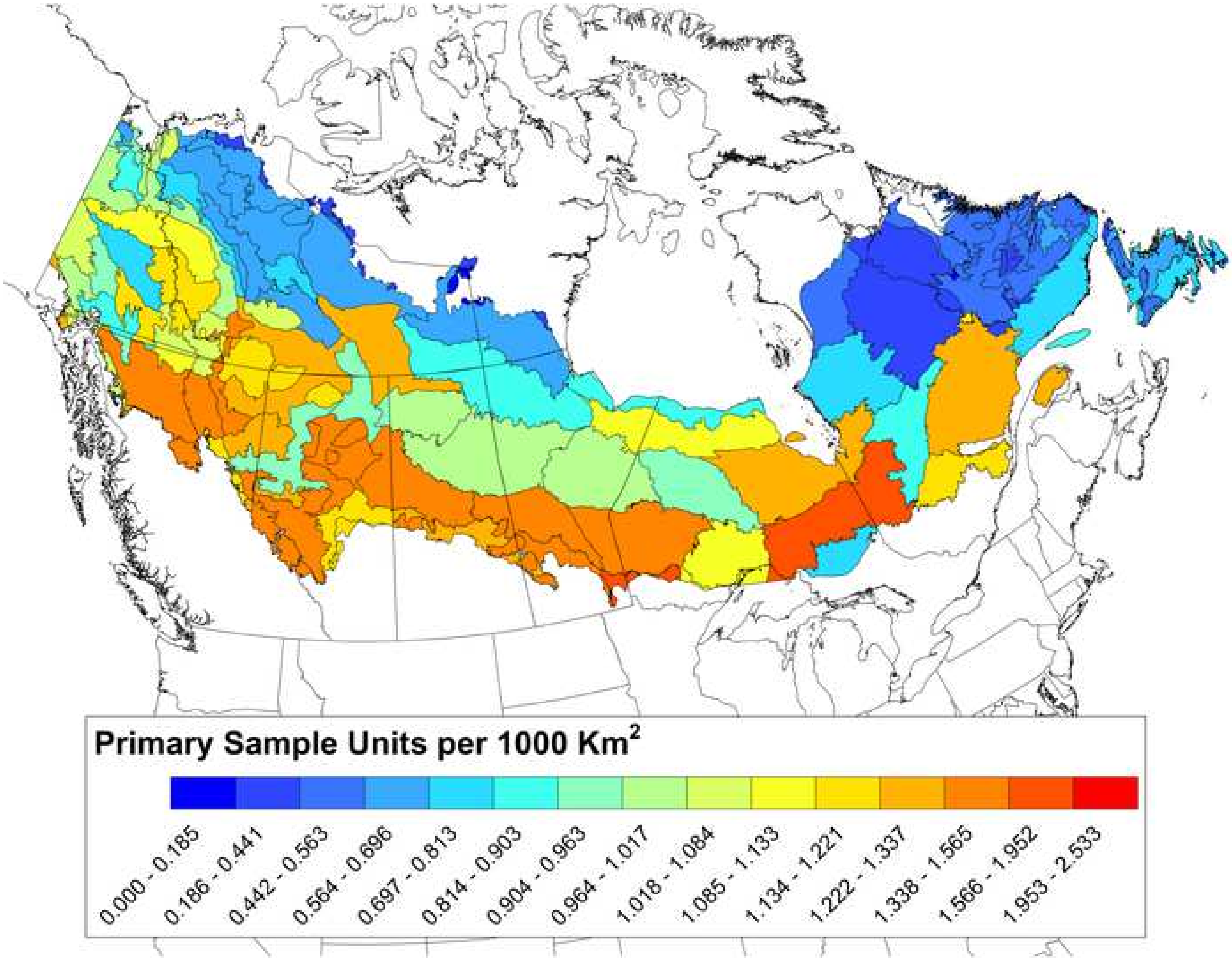

Figure 6

Click here to download Figure Fig 6.tif

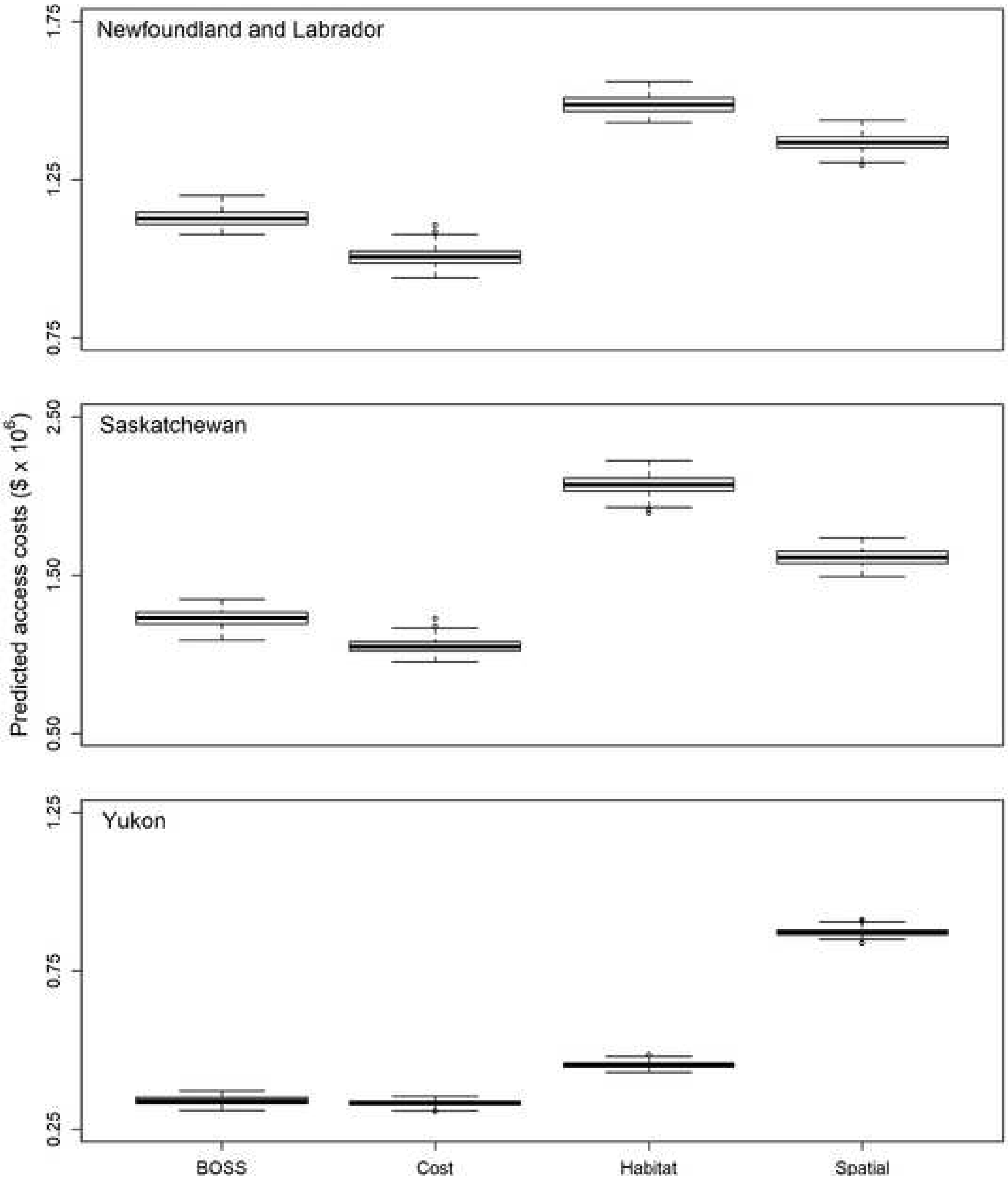

Figure 7

Click here to download Figure Fig 7.tif

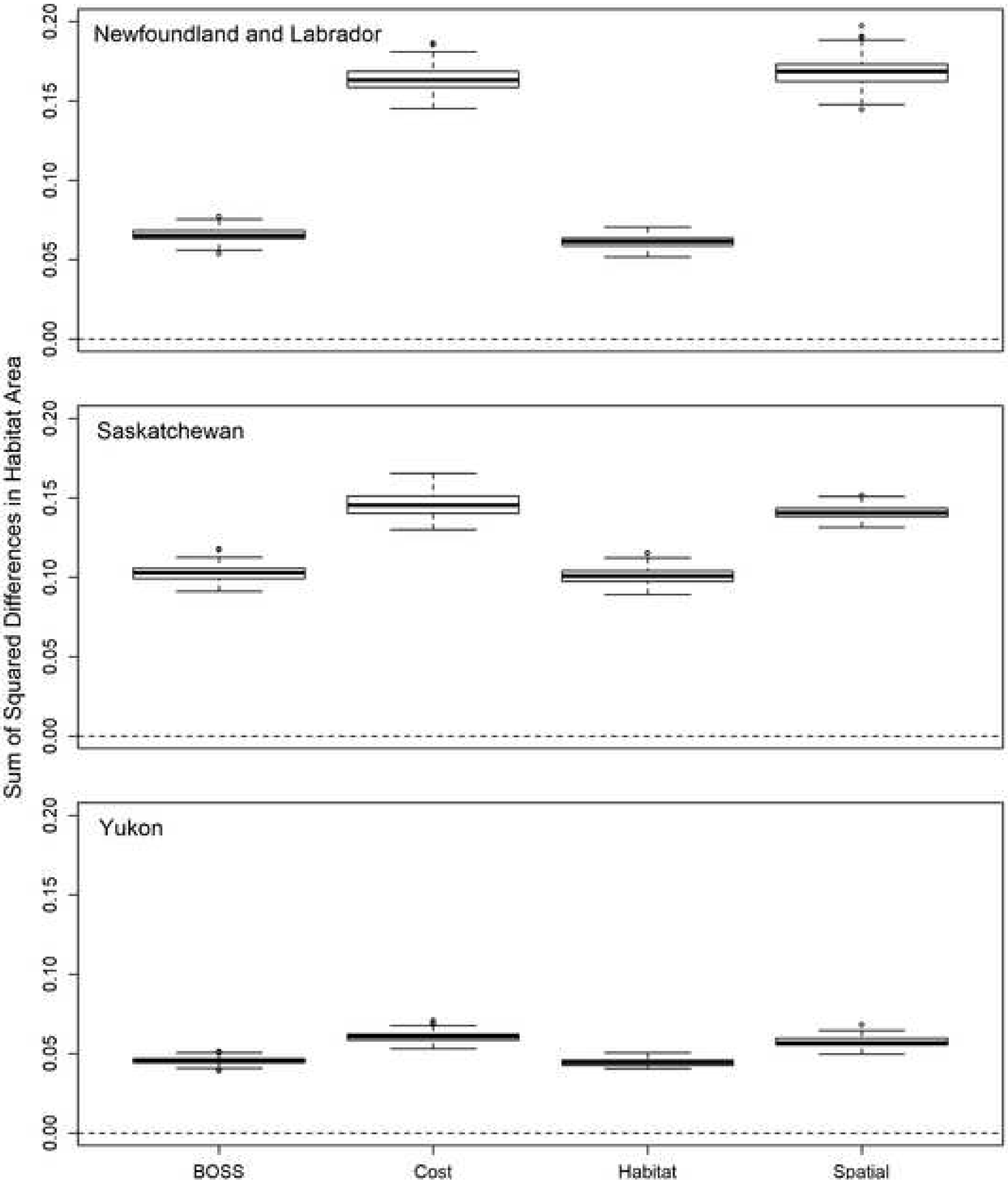

Figure 8

Click here to download Figure Fig 8.tif

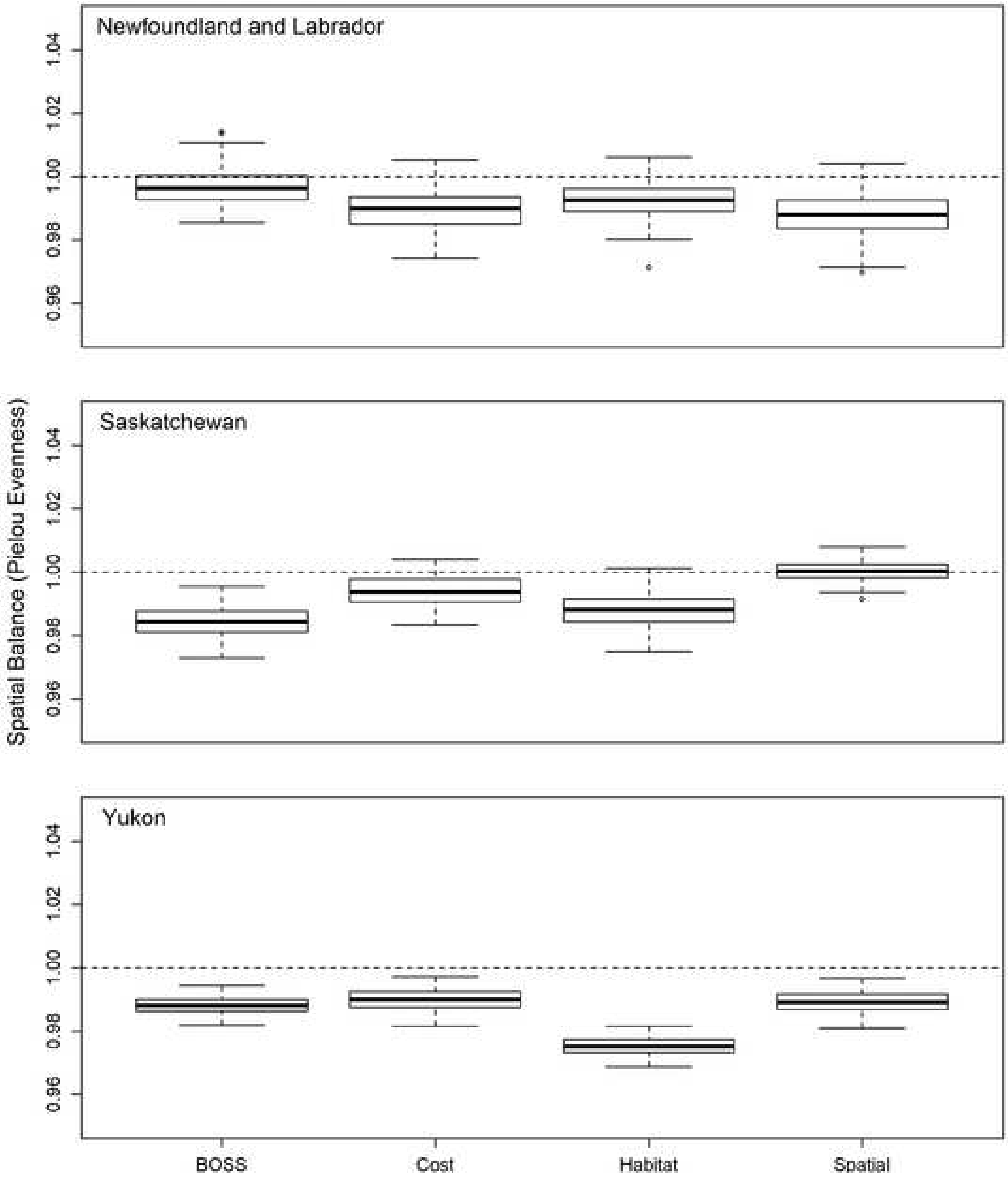

Figure 9

Click here to download Figure Fig 9.tif

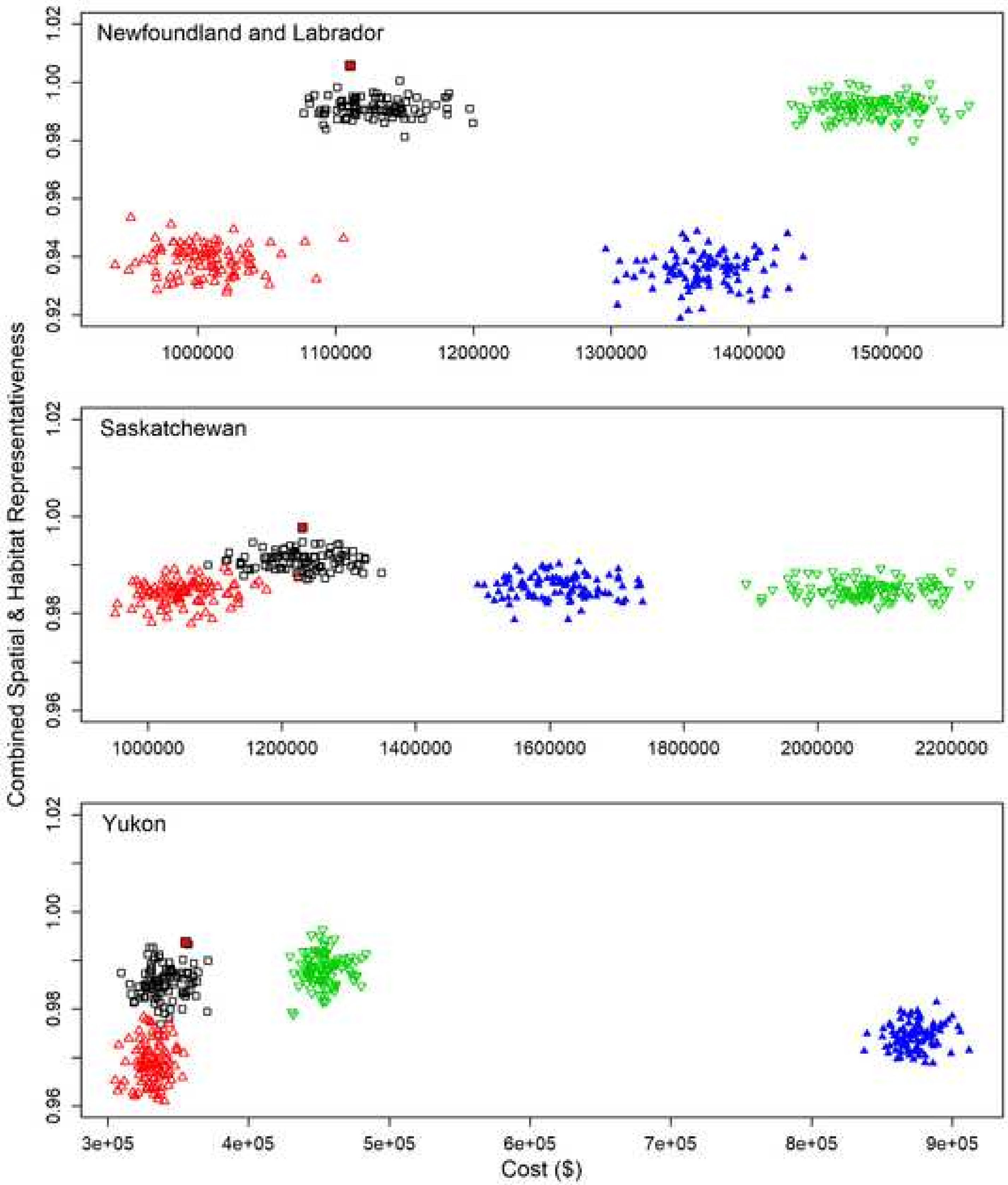

Figure 10

Click here to download Figure Fig 10.TIF

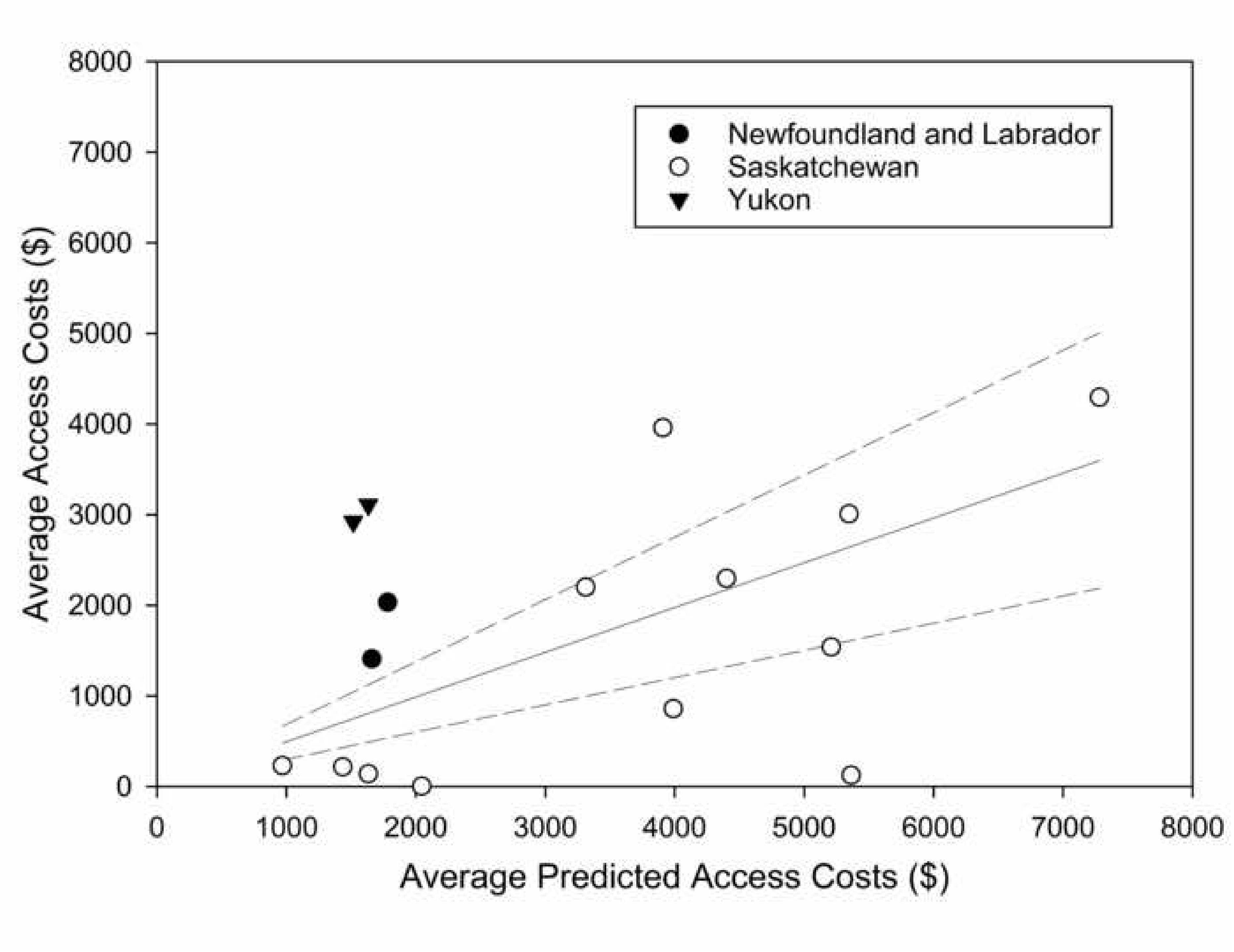

Supporting Information S1. Ecoregion sample sizes for Canada

Click here to access/download

Supporting Information

Sample_Size_Allocations.xlsx

Supporting Information S2. R script for conducting primary and secondary sample unit selection and sample site selection script

Click here to access/download
Supporting Information
BOSS_SiCode2019_20191218.r

Supporting Information S3. Landcover data for habitat stratification from Latifovic et al [44].

Click here to access/download

Supporting Information - Compressed/ZIP File Archive

Landcover.zip

Supporting Information S4. Shapefiles for required to run example analyses in script S2.

Click here to access/download

Supporting Information - Compressed/ZIP File Archive

Shapefiles.zip
