# Supplementary material for: A cost efficient spatially balanced hierarchical sampling design for monitoring boreal birds incorporating access costs and habitat stratification
Source: PLoS One. 2020 Jun 16;15(6):e0234494. doi: 10.1371/journal.pone.0234494 (PMC7297386; doi:10.1371/journal.pone.0234494)
Supplement: Attachment — Submitted filename: BOSS_PLoS_ONE_Revision1_fhkoch.docx [file pone.0234494.s011.docx]

RESEARCH ARTICLE

**A cost efficient spatially balanced hierarchical sampling design for monitoring boreal birds incorporating access costs and habitat stratification**

Steven L. Van Wilgenburg^1^*, C. Lisa Mahon^2,3^, Greg Campbell^4^, Logan McLeod^2^, Margaret Campbell^2^, Dean Evans^5^, Wendy Easton^6^, Charles M. Francis^7^, Samuel Haché^5^, Craig S. Machtans^2^, Caitlin Mader^8^, Rhiannon F. Pankratz^5^, Rich Russell^7^, Adam C. Smith^7^, Peter Thomas^9^, Judith D. Toms^3,8^, Junior A. Tremblay^10^

^1^Environment and Climate Change Canada, Saskatoon, SK, Canada

^2^Environment and Climate Change Canada, Whitehorse, YT, Canada

^3^Department of Biological Sciences, University of Alberta, Edmonton, AB, Canada

^4^Environment and Climate Change Canada, Mount Pearl, NL, Canada

^5^Environment and Climate Change Canada, Yellowknife, NT, Canada

^6^Environment and Climate Change Canada, Vancouver, BC, Canada

^7^Environment and Climate Change Canada, Ottawa, ON, Canada

^8^Environment and Climate Change Canada, Edmonton, AB, Canada

^9^Environment and Climate Change Canada, Sackville, NB, Canada

^10^Environment and Climate Change Canada, Quebec City, QC, Canada

*Corresponding author: [steven.vanwilgenburg@Canada.ca](mailto:steven.vanwilgenburg@Canada.ca) (SVW)

**Abstract**

Predicting and mitigating impacts of climate change and development within the boreal biome requires a sound understanding of factors influencing the abundance, distribution, and population dynamics of species inhabiting this vast biome. Unfortunately, the limited accessibility of the boreal biome has resulted in sparse and spatially biased sampling, and thus our understanding of boreal bird population dynamics is limited. To implement effective conservation of boreal birds, a cost-effective approach to sampling the boreal will be needed. Our objective was to devise a sampling scheme for monitoring boreal birds that would improve our ability to model species-habitat relationships and monitor changes in population size and distribution. A statistically rigorous design to achieve these objectives would have to be spatially balanced and hierarchically structured with respect to ecozones, ecoregions and political jurisdictions. Therefore, we developed a multi-stage hierarchically structured sampling design known as the Boreal Optimal Sampling Strategy (BOSS) that included cost constraints, habitat stratification, and optimization to provide a cost-effective alternative to other common monitoring designs. Our design provided similar habitat and spatial representation to habitat stratification and equal-probability spatially balanced designs, respectively. Not only was our design able to achieve the desired habitat representation and spatial balance necessary to meet our objectives, it was also significantly less expensive (1.3−2.6 times less) than alternative designs we considered. Field implementation of the design in three vastly different regions over three field seasons showed that the approach can be implemented in a wide variety of logistical scenarios and ecological conditions. We provide worked examples and scripts to allow our approach to be implemented or adapted elsewhere. We also provide recommendations for possible future refinements to our approach, but recommend that our design be implemented to provide unbiased information to assess the status of boreal birds and inform conservation and management actions.

# **Introduction**

Tackling ongoing [1,2,3] and projected [4,5] global biodiversity losses will require difficult decisions about resource allocation and the need to consider when, where, and how to focus conservation efforts. Species conservation is often most successful and least expensive when implemented early, before species require dedicated recovery efforts [6]. Early identification of species declines should also improve the likelihood of successful conservation. In addition, developing effective conservation strategies requires identification of the locations and factors contributing to species declines [7]. Sparse or biased data may inaccurately identify which species require conservation actions or misidentify key drivers, resulting in misdirected or ineffective conservation actions [8]. Thus, well-designed ecological monitoring is necessary to effectively prioritize conservation activities [9,10,11].

Even comparatively well monitored taxa such as terrestrial birds have significant gaps in species coverage that hinder effective status and trend assessments and associated conservation actions [12]. In North America, many species of terrestrial birds are primarily monitored using the North American Breeding Bird Survey (*hereafter* BBS; [12]). However, this survey, which is based on roadside surveys and mainly run by volunteers, has very limited coverage in remote northern locations such as the boreal forest [13,14]. Furthermore, much of the coverage for species that breed in the boreal forest is limited to the southern edges of their range, where population change may be quite different from elsewhere. The lack of data from most of the boreal forest may present significant risks to conservation given expanding resource development [15,16,17] and projected climate change impacts on boreal birds [18].

Monitoring data from northern ecoregions are needed to inform conservation actions (e.g., prioritize selection for protected areas), contribute to management decisions (e.g., adaptive management), detect range shifts, assess threats to species (e.g., species response to developments), and meet legislative requirements. In Canada, monitoring data are required to support conservation under the Migratory Birds Convention Act (1994, c. 22), which includes informing listing decisions under Canada’s Species at Risk Act (*hereafter* SARA) [19], and environmental impact assessments required by the Impact Assessment Act [20], or provinces and territories. Key criteria used by the Committee on the Status of Endangered Wildlife in Canada (COSEWIC) to recommend listing a species under SARA include rate of decline in the total number of mature individuals, population size, and extent of species occurrence [21]. In addition, habitat- and region-specific estimates of species’ abundance or density are required as baseline data in environmental impact assessments [20]. To support these diverse requirements, multi-species bird monitoring should be capable of accurately capturing not only trends in species’ population sizes, but also changes in both spatial and temporal variation in species status and distribution.

Given the breadth of these objectives and the large and remote areas to be monitored, a cost-effective sampling approach is required. As most of these locations have limited road networks [22] with few human settlements, there is little potential for volunteer surveys [23]. Similarly, a simple, design-based sampling strategy (e.g., a randomized design in which sample units are selected with the same probabilities) is logistically and financially impractical [9,24]. Spatially balanced sampling offers an attractive alternative as it tends to be more efficient, providing more precise estimates than alternative designs [25,26,27,28]. Despite the advantages offered by spatial sampling designs, they have not been more widely implemented in large-scale (e.g., biome- or continent-wide) studies, perhaps because they force sampling into locations that can be costly to access. Instead, we propose a design-based sampling scheme that integrates multiple approaches from the statistical sampling-theory literature including spatially balanced sampling [25,26], stratified sampling [29,30,31,32], cost-constrained sampling [31] and optimization [33,34]. The integration of these approaches facilitates simultaneous maximization of both spatial balance (i.e. evenness of the spatial coverage) and habitat representation while minimizing program costs.

Here, we describe a cost-effective hierarchically structured unequal probability sampling design known as the Boreal Optimal Sampling Strategy (BOSS). We compared our design against three competing spatially balanced sampling designs: a purely spatial (equal-probability) design, a habitat stratification design, and a design that minimized access costs. Specifically, our objectives are to: (1) outline the development of the BOSS sampling design; (2) describe BOSS design implementation in three ecologically distinct regions in Canada (Newfoundland and Labrador, Saskatchewan, Yukon Territory); (3) evaluate how the BOSS design meets spatial balance, habitat representation, and cost objectives in these three distinct regions; and (4) evaluate implementation costs in field trials. We hypothesized that the BOSS design should provide the second best habitat representation after the habitat stratification design since we incorporated habitat in the inclusion probabilities. We also hypothesized that our BOSS design should rank as the second-least expensive alternative after the cost design owing to incorporation of costs within the inclusion probabilities.

# **Materials and methods**

## **Sampling frame**

We defined the target population as all adult individuals of terrestrial bird populations occupying an ecoregion, province/territory (*hereafter* jurisdiction), and Bird Conservation Region (BCR) within the boreal region of Canada [35] during the breeding season for a particular year. We defined the Primary Sampling Unit (PSU) as a 5 km diameter hexagonal grid cell (i.e. circumscribed by a circle with a radius of 2500m). We generated a hexagonal grid of PSUs covering the entire sampling frame: the boreal region of Canada, plus a 100 km northern buffer (Fig 1). We used hexagonal geometry because it introduces fewer edge effects and performs well in nearest neighbour analyses since the distances between centroids are the same in all directions [36]. We included a northern buffer to allow the design to capture possible breeding range shifts associated with climatic changes in the north [37]. Each PSU was assigned a unique number/identifier. With the exclusion of PSUs that have cumulative inclusion probabilities of zero (e.g., exclusively open water or having too little land to fit secondary sampling locations), all PSUs in the sampling frame were available to be sampled and as a result. As a result, the BOSS sampling design can make valid inferences to entire populations of species inhabiting the boreal region [38].

## **Stratification**

We developed a hierarchical stratification scheme to estimate population variables at multiple spatial scales. At the first level, we stratified the sampling frame using the intersection of BCRs and jurisdictions (e.g., area of Yukon Territory within BCR 4-Northwestern Interior Forest). At the second level, we further stratified each BCR by jurisdiction combination by ecoregion (e.g., the area of Selwyn Mountains ecoregion within Yukon Territory within BCR 4), where ecoregions are a subdivision of an ecozone characterized by similarity in surface forms, flora, fauna, hydrology, soil, and macro climate [39]. Regional monitoring programs as part of the BOSS design are defined by the area of all boreal ecoregions falling within a given jurisdiction. We set target sample sizes for the number of PSUs to select within an ecoregion using the process outlined below (see sample size allocation).

### Sample size allocation

From sampling theory, a stratified sample is optimal when it provides the greatest precision for the lowest cost [40]. Optimal stratified sampling accounts for both the size of the stratum (e.g., land-area for geographic regions), and the variance within the stratum [40]. By placing greater sampling effort in strata that are larger and more variable, the overall precision of the estimate(s) is optimized [29,31]. Here, we calculated the sample size of PSUs (n) for a given jurisdiction using the following equation to allocate sampling effort between all (*k*) ecoregions within the jurisdiction:

$n_{e}=N*\frac{{Area}_{e}{* \sigma}_{e}*r_{e}}{\sum_{e_{1\ldots k}} {Area}_{e}{* \sigma}_{e}*r_{e}}$	(1)

Where n*_e_* is the target sample size for a given ecoregion within a given jurisdiction, *N* is the total target sample size to sample within the jurisdiction, *Area_e_* is the area of ecoregion *e*, *σ_e_* is a measure of observed or predicted variation within ecoregion *e* for the variable(s) of interest, and *r_e_* is the described species richness of the bird community in ecoregion *e* (i.e., the number of all bird species with ranges overlapping the ecoregion derived from overlays of species range maps with the ecodistricts of Canada [39] by GeoInsight Corporation [41]). Since our goal is to monitor multiple species, employing a univariate measure of variance would provide an inadequate representation of variance [31]. We included species richness as a weighting factor on within-stratum variation (*σ_e_*), to increase sampling in ecoregions where more species occur and improve the (1) likelihood of adequately monitoring a greater number of species, and (2) ability to monitor and predict species distributions.

Many of the ecoregions in our sampling frame have little or no historic sampling from which we could derive estimates of spatial or temporal variance in the bird community. As an alternative, we used proxy variables that correspond with the spatial and/or temporal variance in bird abundance within an ecoregion to set relative PSU sample size targets for the ecoregion (i.e., as proxies for *σ_e_*). We used these proxy variables [31] to calculate a measure of multivariate dispersion (variance estimate) for each ecoregion following [42,43], representing anticipated spatial and temporal variation in bird abundance and distribution. We used several well-described links between species abundance and distribution and spatial and temporal climatic variation [44], vegetation [44,45] and variation in forest age [45,46]. Specifically, we used the following proxy variables: (1) variance of mean temperatures for May-July across years (1981-2014); (2) variance of total precipitation for May-July across years (1981-2014); (3) standard deviation of annual burn rates (% area burned by wildfire per year [47]) between 1980 and 2015 within an ecodistrict; (4) richness of landcover classes [48] within an ecodistrict; (5) standard deviation of elevation; and (6) percent area of open water since riparian edges significantly increase avian species richness [49]. We calculated variance of mean May-July temperatures and variance of total precipitation from monthly gridded (0.5 x 0.5 degrees) values from the climate datasets of Harris et al. [50]. We calculated the between year standard deviation of burn rates from data obtained from [47]. Since burn rates from [47] were calculated in sample units of 10,000 km^2^ hexagons, we first calculated the between year standard deviation of burn rates within hexagons, and derived the mean of those values for ecodistricts. Landcover of Canada 2010 [48] is a 250 m resolution land cover layer of 15 land cover classes from 13,350 Landsat-7 satellite images taken between 2009 and 2011. Ecodistricts (subdivisions of ecoregions characterized by distinctive landforms, relief, geology, soils, water bodies, flora and fauna [39]) were treated as sampling units to allow us to calculate dispersion within ecoregions. Proxy variable data are provided in Supporting Information S1.

## **Sampling design**

We developed a two-stage sampling design in which the first stage of the design involved selection of PSUs from ecoregions within the sampling frame and the second stage involved selection of Secondary Sampling Units (SSU) within the selected PSUs (Fig 2). The SSU represent on-the-ground locations where point count surveys [51] will be conducted using trained observers and/or autonomous recording units (*hereafter* ARUs) [52].

### Selection of primary sampling units

To select PSUs within each jurisdiction (Stage 1) we first developed an approach to combine the following three design components: access costs, habitat representation, and spatial balance. We followed the steps outlined below in each of our three test jurisdictions (Newfoundland and Labrador, Saskatchewan, and Yukon Territory) to integrate all components of our sampling design across our sampling frame (Fig 3). First, we created separate cost surfaces for logistically feasible access methods within each of our jurisdictions. Specifically, we developed spatially explicit models predicting the cost to access a given PSU assuming access from roads (trucks), helicopters, float planes, snowmobile and/or all-terrain vehicles as appropriate for a given jurisdiction (see examples in Supporting Information S2). The resulting cost models were represented as pixel-based (250 x 250m) estimates of cost, from which we subsequently calculated the average cost of access for all pixels within a PSU separately for each access type (e.g., trucks, helicopters, float planes). We assumed that the lowest-cost access method will generally be used to access a given PSU and calculated the minimum cost across all access cost surfaces. We subsequently used this value as the estimated access cost for each PSU.

Second, we calculated inclusion probabilities based on landcover classes (hereafter habitat classes) from the North American Land Change Monitoring System [48]. The landcover product of Latifovic et al. [48] consisted of nineteen landcover classes in total, four of which only occur in the tropics and therefore there were only fifteen of relevance in our sampling frame. We calculated habitat-based inclusion probabilities in an attempt to achieve balanced representation of habitat classes within an ecoregion. Inclusion probabilities for habitat classes used a weighted sampling approach to balance sampling across common (high occurrence/high proportion) and uncommon (low occurrence/low proportion) habitats within an ecoregion. Weighted habitat inclusion probabilities were calculated as follows:

${p_{habitat}}_{pie}=\frac{\frac{1}{{\# of Habitat Classes in ecoregion}_{e}}}{{Area}_{ie}}$	(2)

Where the inclusion probability for a given pixel *p* of habitat class *i* within a given ecoregion (*p_habitatice_*) was a function of the inverse of the number of habitat classes within the ecoregion divided by the area of habitat *i* within ecoregion *e*. Our weighted habitat inclusion probability thus results in the cumulative probability of sampling a given habitat within an ecoregion being equal among all habitat classes despite differences in habitat area, to help ensure that we can draw inferences for rare habitat types.

Third, we combined access costs with weighted habitat inclusion probabilities to derive our final summed value for inclusion probabilities for each PSU as follows:

$I_{{SU}_{k}}=\sum\frac{{p_{habitat}}_{{pie}_{1\ldots j}}* \frac{1}{\sqrt{{cost}_{k}}}}{\sum{p_{habitat}}_{{pie}_{1\ldots j}}* \frac{1}{\sqrt{{cost}_{k}}}}$	(3)

Where *I_SUk_* = the sum of all inclusion probabilities within a given primary sample unit (PSU) *k*, *P_habitatpie_* = area-weighted selection probabilities for habitat class *i* for pixels (*p*) 1 through *j* in ecoregion *e* per equation 2, and cost for a given PSU was the minimum access cost for sample unit *k* (as described above). Cost was included as the inverse square root of costs to preferentially (all else being equal) favour sampling at PSUs that are less expensive to sample in accordance with optimal allocation theory [53]. Across all sample units within the given study area, the inclusion probabilities sum to unity based on equation 3.

Fourth, we used unequal-probability sampling based on the calculated inclusion probabilities (*P_SUk_*) to draw a spatially balanced sample within each ecoregion using Generalized Random Tessellation Stratified sampling (GRTS; [25]). In brief, the GRTS approach maps two-dimensional space into a single dimension (that maintains the spatial properties of the original space) and takes a systematic sample along that representation, yielding a sample that is neither over- nor under-represented across space. For a detailed description, we refer the reader to [25] and [30]. The random draw to select PSUs within ecoregions in each jurisdiction uses PSU sample size targets for each ecoregion (where ecoregion boundary was clipped to the jurisdiction boundary). We included a 20% oversample of PSUs for each ecoregion to allow substitution of inaccessible or unsafe PSUs (e.g., cliffs or other features not visible in the available input layers). All PSUs were labeled with their draw order and oversample PSUs were additionally labeled as oversamples. Should a PSU be inaccessible or unsafe to survey, the oversample and draw order can be used to select a replacement PSU that retains the spatial balance of the sample [25].

Finally, we derived an approximately optimal draw of PSUs using a combination of repeated random sampling and multi-criteria evaluation. Specifically, we propose that multiple random unequal-probability GRTS samples can be obtained for which the predicted sampling costs and metrics of habitat representation and spatial balance can be derived. The iteration resulting in the highest combined representativeness of habitat sampling combined with spatial balance for the lowest predicted sampling cost should provide an approximately optimal design. As a metric of habitat representation, we calculated the sum of squared differences (SSD) between the area of each habitat class within all PSUs in a randomized draw versus the proportional area of each habitat class. As a metric of spatial balance, we use Pielou evenness (*PE* in eq. 4 below) to represent the even spread of PSUs across the sample frame [30]. Possible values of *PE* range from 0–1. In order to evaluate which draw represents the best trade-off of cost versus combined spatial and habitat representation across multiple (1…*j*) iterations, we propose evaluating which iteration minimizes predicted costs while maximizing the weighted sum [34] of the two metrics:

$\left( \left( 1-\left( {SSD}_{i}-\min\left( {SSD}_{i\ldots j} \right) \right) \right) \bullet w_{h} \right)+ \left( w_{s} \bullet{PE}_{i} \right)$	(4)

Where *SSD* for sample draw *i* is relativized to the minimum *SSD* across draws 1 through *j*, and subtract the resulting value from 1 so that the metric scales from 0–1 and thus a value of 1 would represent perfect representation of habitat composition within the draw. We applied equal weights to both the habitat (*w_h_*) and spatial balance (*w_s_*) components as we did not want either component to be more heavily valued than the other in our final design (i.e., *w_h_ = w_s_* = 0.5). However, this approach can be generalized by allowing weights to vary between habitat representation and spatial representation if there is a desire to emphasize habitat representation (e.g., for environmental impact assessments) or spatial representation (e.g., for distribution modeling).

### Selection of secondary sampling units

To select secondary sample units (SSU) within each PSU in each jurisdiction (Stage 2), we first created a uniform (systematic) grid of potential secondary sample unit locations at 300 m spacing over the extent of each ecoregion. Each SSU was separated by 300 m since the effective detection radius for most boreal songbirds is less than 150 m [51] and therefore double counting should be infrequent for most species. We used the grid of SSUs to query the habitat-based inclusion probabilities. Within each PSU, we selected a stratified random sample of four SSUs where higher inclusion probability values (indicating rare/uncommon habitat classes) influenced the selection of SSUs. We used the four SSUs selected during this draw as plot centroids and the eight SSUs surrounding the plot centroid to construct plots of nine SSUs for sampling (see Fig 2). We set a sampling target of two plots of nine SSUs per PSU so that a team of two staff can complete one PSU per day in most circumstances. Furthermore, having two survey plots within a PSU allows staff to have a partner(s) to provide support in an emergency situation. Our PSU size of 5 km diameter was specifically selected to ensure field staff would be working within a reasonable walking or flight distance if assistance or emergency response is required. We drew a sample of four plots of SSUs per PSU to create an oversample in case of inaccessibility (e.g., due to large water barrier) or safety concerns (e.g., hazardous terrain or aggressive wildlife).

### Data collection

We tested the BOSS design within three jurisdictions across the boreal biome of Canada (Newfoundland and Labrador [NL], Saskatchewan [SK], and Yukon Territory [YT]) during 2017, 2018, and 2019. Newfoundland and Labrador is located at the eastern edge of the boreal region within BCR 7-Taiga Shield and Hudson Plains and BCR 8-Boreal Softwood Shield (Fig 1). Saskatchewan is located in the center within BCR 6-Boreal Taiga Plains, BCR 8, and BCR 7 (see Fig 1); while the Yukon Territory is located at the northwestern edge within BCR 4-Northwestern Interior Forest, BCR 6, and BCR 3-Arctic Plains and Mountains. Jurisdictions were also ecologically distinct, differing in their geology, topography, vegetation, terrestrial bird diversity, and climate as indicated by the number of ecozones (NL: 2; SK: 3; YT: 3), ecoregions (NL: 23; SK: 8; YT: 22), and elevation range (NL: 0−1,652 m; SK: 204−823 m; YT: 0−5,900 m). Differences in the location of mountain ranges, rivers and lakes, human settlements, and primary and secondary road networks within each jurisdiction present distinct logistical challenges and therefore useful comparative test cases. To conduct point count surveys at SSUs within selected PSUs we followed standard recommended protocols of Ralph et al. [54] and Matsuoka et al. [51] and conducted point count surveys during suitable weather conditions from 30 minutes prior until 4-5 hours after sunrise, from the last week of May to the first week of July. We conducted 10-minute point counts using trained observers following protocols outlined by Matsuoka et al. [51] or we analysed 10-minute recordings from ARUs [52]. ARUs were deployed on a variety of different schedules and using multiple access methods. In some cases, ARUs were deployed in February by snowmobile, and were retrieved in the summer. Wherever possible, we attempted to get recordings conducted over ≥ 4 mornings with good survey conditions; however, ARUs carried on canoe trips were deployed for as little as a single night. We programmed ARUs to record a minimum of six 10-minute intervals over the course of a morning during the same time-period as point counts, as well as additional times for other objectives. An analysis of the relative merits of different programming schedules and a comparison of field observers with ARUs is beyond the scope of this paper.

We sampled 295 PSUs selected using the BOSS design between 2017−2019 in Newfoundland and Labrador (n = 69), Saskatchewan (n = 103) and Yukon Territory (n = 123). Three PSUs in Saskatchewan had to be replaced with PSUs from the oversample owing to access and safety issues associated with open-pit uranium mines. No oversamples were required in either Newfoundland and Labrador or Yukon Territory. In Newfoundland and Labrador, we sampled 5 out of 9 ecoregions on the island of Newfoundland, with all ecoregions being in BCR 8. PSUs were sampled in the Avalon Forest ecoregion (n = 13), Central Newfoundland (n = 1), Maritime Barrens (n = 8), South Avalon-Burin Oceanic Barrens (n = 23), and Southwestern Newfoundland (n = 24). We sampled seven out of eight ecoregions within Saskatchewan: Selwyn Lake Upland (BCR 7, n = 12), Tazin Lake Upland (BCR 7, n = 9), Athabasca Plain (BCR 8, n = 19), Churchill River Upland (BCR 8, n = 22), Mid-boreal Uplands (BCR 6, n = 28), Mid-boreal Lowland (BCR 6, n = 5), and the Boreal Transition (BCR 6, n = 8). We sampled ten out of 22 ecoregions within Yukon Territory, sampling in the British-Richardson Mountains (BCR 3, n = 1), Old Crow Basin (BCR 4, n = 9), Old Crow Flats (BCR 4, n = 2), Eagle Plains (BCR 4, n = 3), North Ogilvie Mountains (BCR 4, n = 5), Mackenzie Mountains (BCR 4, n = 8), Klondike Plateau (BCR 4, n = 72), Yukon Plateau Central (BCR 4, n = 10), Yukon Plateau North (BCR 4, n = 10), and Ruby Ranges (BCR 4, n = 3).

### Ethics statement

We obtained the required permits to conduct avian monitoring to test the BOSS design from provinces/territories, Provincial and Territorial Parks, Parks Canada Agency, and Yukon First Nations with Traditional Territories and Settlement Areas. Field observers spend <15 minutes at SSU locations during observer surveys and <30 minutes (time for set up and subsequent retrieval) at SSU locations during ARU surveys, resulting in minimal disturbance to breeding birds.

## **Statistical analyses**

Prior to analysis of our multivariate proxy data, we centered and standardized all variables to zero means and unit variance and then calculated Euclidean distances between all points (ecodistricts) in the dataset. We derived the multivariate estimate of dispersion by calculating mean distances to ecoregion centroids using the ‘betadisper’ function in the vegan package [55] in the R statistical computing environment [56]. The resulting estimates of dispersion for all ecodistricts within the boreal region, as per Brandt [35], are available in Supporting Information S3.

In order to assess our BOSS design, we used the ‘spsurvey package’ [57] within the R statistical computing environment [56] to draw samples under four alternative spatially balanced sampling designs. Specifically, we drew samples under one equal-probability spatially balanced design and three unequal-probability designs: (a) a habitat stratification design, calculated as per equation (2) above; (b) a cost design in which inclusion probabilities were based solely on access costs as per equation (3); and (c) our BOSS design in which inclusion probabilities included both habitat stratification and access costs as per equation (4) above. For each scenario, we ran 100 iterations in which we used the GRTS algorithm [57] to draw a random sample of n = 400 PSUs from each of the three jurisdictions considered here. Within each iteration, our script calculated the access costs (CDN $), spatial balance (Pielou evenness [30,57]), and habitat representation as SSD (see above). All implementations are available within scripts in the supplemental material (Supporting Information S2 and S6) along with geospatial data examples (Supporting Information S4, S5 and S7).

We used linear models to test for differences (for each jurisdiction separately) in each of our response variables (cost, SSD, and Pielou evenness) between the alternative designs described above (i.e., spatial, habitat, cost, and BOSS). Study design was included as a factor, and in each model we specified an *a priori* reference factor level to test our hypotheses. We specified the cost design as the reference factor level for models examining access costs, as we anticipated this design should have the lowest access costs on average. Similarly, we specified the habitat stratification design as the reference level for models examining variation in SSD as we predicted this design should have the lowest SSD.

All four designs we considered employed a GRTS algorithm to generate spatially balanced samples; however, the use of unequal inclusion probabilities based on spatially structured variables could alter the ability of the design to achieve spatial balance. We therefore tested for differences in spatial balance between our designs and we treated the spatial design (equal-probability spatial sampling design) as the reference factor level when comparing spatial balance between sampling designs. We calculated robust standard errors (‘sandwich’ estimators) to overcome problems with heteroscedastic errors using the ‘lmtest’ [58] and ‘sandwich’ [59] packages within the R statistical computing environment [56]. All data are available in the Supporting Information (S8).

# **Results**

## **Sample Size Allocation**

Sample size allocation to geographic strata (ecoregions × jurisdiction) under the BOSS design resulted in a similar sample size on average as allocating samples based on stratum area, because data are centered along a 1:1 correspondence line (Fig 4). However, allocation to individual strata differed markedly, with the BOSS design suggesting significantly lower sample sizes for some strata than an area-based allocation, and allocating disproportionately larger sample sizes in other strata (Fig 4). For example, sampling proportional to area would allocate 151 PSUs to the New Québec Central Plateau ecoregion of Québec compared to only 50 based on the BOSS allocation because the stratum has lower than average dispersion (i.e., 0.36 vs average of 1.04). In contrast, 103 PSUs would be allocated to the Abitibi Plains ecoregion of Ontario under sampling proportional to stratum area, but 173 PSUs were allocated to the same stratum under the BOSS design owing to higher than average dispersion (i.e., 20.1 vs. average of 1.04). Several of the larger and more variable strata (sample sizes > 100) require more sampling under the BOSS design than predicted solely based on stratum area (Fig 4). Overall, the BOSS sample size allocation generally results in sampling intensities being greatest in the south and decreasing northward (Fig 5).

## **Predicted sampling costs**

Predicted costs to access 400 primary sample units varied by the jurisdiction and sampling design considered (Fig 6). Across all three jurisdictions, designs in which the inclusion probabilities derived solely based on access costs (cost design) were the least expensive designs (Fig 6) and all the alternative designs were more expensive regardless of jurisdiction (Table 1). The BOSS design had the next lowest cost (Fig 6), with costs predicted to be between 1.02 (Yukon Territory) and 1.2 (Saskatchewan) times more expensive on average than the cost design for a given jurisdiction (Table 1). In both Newfoundland and Saskatchewan the equal-probability spatial design had the third-lowest cost followed by the habitat stratification design, whereas it was the most expensive design for Yukon Territory (Fig 6); it was predicted to be 1.4−2.6 times more expensive on average (~$362,000−562,000; Table 1) than the cost design (maximum of 1.5−3.0 times; Fig 6). The habitat stratification design was predicted to be 1.4−2.0 times more expensive on average (~$122,000−1,020,000; Table 1), or a maximum of 1.6−2.3 times more expensive across draws and jurisdictions (Fig 6).

## **Habitat representation**

As predicted, the habitat stratification design had the lowest sum of squared differences between the habitat areas represented within sample draws and the desired equal habitat representation (Fig 7). The BOSS design provided the second-most representative sampling of habitat classes on average across all three jurisdictions (Fig 7; Table 2). In contrast, both the equal-probability spatial design and the cost design (Fig 7) had substantially lower habitat representation relative to the habitat stratification design in all three jurisdictions (Table 2).

## **Spatial representation**

All sampling designs provided relatively balanced spatial sampling of all three jurisdictions (range of Pielou evenness across all designs: 0.96−1.01; Fig 8). Interestingly, the spatial balance of the cost design provided the closest approximation to the equal-probability spatial design (Fig 8) and with substantial overlap of the data distribution compared to the equal-probability spatial design (Table 3). The BOSS design showed small differences in spatial balance relative to a purely spatial design in both Newfoundland and Yukon Territory (Fig 8, Table 3), but not Saskatchewan (Table 3). The habitat stratification design had the largest overall differences in spatial balance relative to the equal-probability spatial design, with the largest difference occurring in Yukon Territory (Fig 8, Table 3).

## **Optimal design selection**

Across 100 random sample draws, the BOSS design provided the best combination of cost savings and both spatial and habitat representation amongst the four designs considered. Regardless of jurisdiction, the BOSS design had costs competitive with the cost design (Fig 6 and 9), with no loss of habitat or spatial representation. For all three jurisdictions combined, selecting the draw that simultaneously maximized representation while minimizing cost (see red-filled squares in Fig 9) only results in an estimated savings of approximately $2,000 relative to the median of all BOSS sample draws. However, access costs for 400 PSUs varied substantially between draws (Newfoundland: $1,076,897−1,199,693; Saskatchewan: $1,089,811 −1,348,550; and Yukon Territory: $309,497−371,174) and thus relative to the most expensive draws for each jurisdiction, the combined savings could be up to an estimated $225,000 for these three jurisdictions when using the draw providing the best trade-off based on our multi-criteria evaluation.

## **Field implementation**

Of the 295 PSUs sampled across all three jurisdictions, we sampled 85 PSUs in remote areas that we predicted would require charter flights to access. In Newfoundland, cost models accurately predicted an average cost of $1,721 (SD = $86) per PSU to access 16 PSUs, compared to actual costs which averaged $1,721 (SD = $441) per PSU (Fig 10). In comparison, the cost models for Saskatchewan predicted an average access cost of $3,741 (SD= $1,928) for the 43 remote PSUs sampled, but the actual access costs were substantially lower (mean = $1,573, SD = $1,562). In Yukon, the mean predicted access cost to reach the 26 remote PSUs sampled was $1,574 (SD = $82; Fig 10) per PSU, but the mean cost to access these PSUs was $3,016 (SD = $132). Ordinary least squares regression suggests across all three jurisdictions the access costs were on average 48% of the model predicted cost on average (β = 0.48, SE = 0.10; Fig 10).

# **Discussion**

We integrated key concepts from sampling theory to develop a hierarchically structured survey design that can provide stratified sampling of predefined strata (e.g., ecoregions) and achieve the desired spatial balance and habitat representation while minimizing access costs. The probability-based BOSS design allows for valid statistical inference from field samples across the sampling frame and reduces operational costs. We found that our integrated design was significantly less expensive than all but a design based only on access cost. Predicted access costs were 1.3−1.7 times more expensive for the habitat stratification design and 1.6−2.7 times more expensive on average for the spatial design compared to our BOSS design. Additionally, the distribution of habitat representation across sample draws from the BOSS design overlapped almost entirely with that from the habitat stratification design (see Fig 7). While we did find minor differences in the distribution of spatial balance between the designs considered (Fig 8), spatial balance was always close to a value of one, indicating the samples were well spread over jurisdictions irrespective of the type of design. Across jurisdictions, spatial balance metrics of the spatial design did not always overlap one, suggesting that the inclusion of unequal sampling probabilities did not introduce systematic biases in spatial representation. Combined with our randomizations to balance trade-offs, we therefore simultaneously achieved habitat and spatial representation goals while minimizing access costs.

Our pilot field seasons suggest that logistic considerations might result in access costs differing from those suggested by our cost models. While cost models for Newfoundland have thus far provided accurate prediction of average access costs per PSU, our cost models underestimated access costs in Yukon. Overall, regression analysis suggested that field implementation was 52% cheaper than suggested by our cost models. Many reasons likely contributed to the cost differences we observed, including external logistical support. One key difference is that our cost models largely portray the cost of access to a sample unit as independent of access to other nearby sampling units. In practice, we were able to combine logistics and access methods for many sampling units and thereby substantially lowered the average cost per sample unit. For example, in the summer of 2019 we chartered a Twin Otter to drop off two crews on or near opposite sides of Cree Lake, Saskatchewan (57°23′57″N 106°40′10″W) to access six PSUs via canoe. During crew retrieval, an additional crew was dropped off to canoe down river to a roadside pickup location, allowing access to two additional PSUs. We were therefore able to access eight PSUs for the cost of two charter flights (the base cost for accessing a single PSU), substantially reducing the average cost per PSU. We therefore recommend that programs revise their cost models as field implementation improves knowledge of local logistics. As a result, final program costs can be lowered by refinement of preliminary cost models.

Our sampling design has several key differences from other large-scale terrestrial bird monitoring programs that improve its utility for remote areas such as the boreal forest. Other programs such as the Integrated Monitoring in Bird Conservation Regions (IMBCR) program [9], the UK Breeding Bird Survey [60], the North American BBS [12] and the US National Park Service Vital Signs Monitoring program [61] also have defined sampling frames and randomly selected sample units that facilitate valid statistical inference from field samples [62]. Importantly, our design has fewer restrictions on the sampling frame while minimizing sampling costs, and implicitly accommodates health and safety considerations. While other programs restrict the sampling frame to roadsides or trail networks to facilitate access and alleviate safety concerns, we instead dealt with access by incorporating access costs into sample unit selection, and designing surveys to include multiple staff within primary sample units. Thus, our design increases safety and reduces costs without limiting statistical inference about bird populations to accessible areas [62,63,64]. In addition, our hierarchical stratification with static strata (BCRs, jurisdictions and ecoregions) allows for assessment of the population-level responses of birds to natural and human disturbance and climate change [9,65]. Indeed, Pavlacky et al. [9] recently demonstrated the ability of their hierarchical spatial design to allow estimation of both strata-specific population sizes and species-habitat relationships. Our approach of integrating both cost and weighted habitat-inclusion probabilities within a similar spatially balanced design will allow us to draw similar inferences to Pavlacky et al. [9] at multiple spatial scales, but at reduced costs compared to alternative approaches.

Our design directly incorporated several factors into the stratification and layout of primary and secondary sampling units to address the need to minimize costs and provide for the health and safety of field staff. Field implementation of the optimal designs selected here during pilot field seasons across all three jurisdictions showed that the BOSS design was achievable on the ground. We were able to apply the design across a wide variety of challenging conditions from mountainous terrain (e.g., >2000 m elevation), wetlands (floating bogs, fens and marshes), forests with blow-down (recent burns, tornados and insect outbreaks), as well as steep, rocky, and densely vegetated slopes across all three jurisdictions. Furthermore, drawing an oversample using the GRTS algorithm allowed us to replace unsafe PSUs without altering the representativeness of the sample [25]. Using pairs of observers to conduct surveys within each PSU means that staff can work alone to each survey a plot of nine SSUs within a PSU or they can work together to complete one plot of nine SSUs before travelling together to complete the second plot. The latter option would be appropriate if a PSU contains dangerous terrain, wildlife or limits to communication between staff (radio, satellite transmitter).

One complication imposed by our design is that most analyses assume that sampling is proportional to habitat availability and, by extension, population sizes. We used habitat stratification to ensure representation across the range of available habitat classes and thereby provide reasonable sample sizes to facilitate modeling abundance and distributions of species associated with rare habitats. Habitat stratification should also improve our future capacity to detect trends of species using rare habitats. In particular, our approach to habitat stratification should increase model precision for species using rare habitats at the cost of reducing precision for common species; a trade-off we feel is worthwhile because estimating trends, distribution and habitat relationships for rare species is always more limiting. However, extrapolating from the sample to estimate stratum-specific population sizes and/or population trends is more complicated because strata boundaries and sizes can change with shifts in vegetation through time [10]. This problem can be addressed analytically via post-hoc stratification [67,68], weighting via the inclusion probabilities [69,70], including random-effects for the strata indexed by the inclusion probabilities [71], or inclusion of interval-specific covariates reflecting habitat supply [70]. Recent spatially explicit approaches to avian population size estimation [72] provide one potential approach to build trend estimation models that could implicitly incorporate changes in strata sizes through time.

While the BOSS design presented here incorporates several key improvements over current large-scale bird monitoring designs, we foresee several areas where further improvements could occur. First, we stratified habitat using coarse satellite-derived products due to their availability and consistency across the entire sampling frame. Incorporating higher-resolution products capable of further stratifying habitat, and potentially better quantifying the amount and distribution of rare habitats, should improve our capacity to monitor species associated with these habitats. One possible approach worth investigation would be to create an avian habitat classification (*sensu* [45]) from higher-resolution data, such as forest inventory data, for the portion of the sample frame for which they may be available. Higher-resolution data could be integrated into the current framework by (1) using the coarse-resolution satellite data to select PSUs and using the high-resolution habitat classification to stratify SSUs within the selected PSUs, or (2) splitting the selection of PSUs between areas with versus without access to these high-resolution data products.

Second, once sufficient on-the-ground data are available, it would be desirable to re-examine the stratification by using the design-based sample to estimate spatial and temporal variance in bird community composition directly for optimal allocation rather than the proxy variables used in the initial sample. We anticipate that sampling efficiency will improve by incorporating these direct estimates of variability into the design, but overall costs could be inflated if these data are not incorporated until they are available for all regions. Using data from preliminary rounds of sampling under the sampling design to validate allocation based on multivariate dispersion of proxy variables will ensure allocation between strata is cost effective, and could be used for second-phase sampling to improve the precision of estimates further [28].

Future simulation studies are needed to examine the likely trend precision that can be achieved under the BOSS design under alternative PSU revisit schedules (e.g. every 5 or 10 years). Evaluating whether the BOSS emphasis on greater sampling in southern regions (Fig 5) could impede our ability to detect trends for northern distributed species or potentially improve our capacity to detect anthropogenic impacts on avian population due to the concentration of anthropogenic disturbance in southern regions would be an important addition to these simulation studies. Finally, optimizing the use and allocation of humans versus ARUs within our design is also a priority for future simulation modelling. Recent studies suggest that biases in species detection between human and ARU surveys can largely be controlled [73,74], but potential biases between single versus multiple visits would ideally be controlled by spatially balanced allocations of survey effort between humans and ARUs. However, logistical constraints and/or the aggregation of hard to detect species (e.g., secretive marsh birds) into a subset of habitat may make other approaches more efficient and is the subject of ongoing work.

To avoid inefficiencies, there is a clear need for improvements in cost models or habitat layers to be reflected in revised sample draws; however, revised sample draws should account for existing sampling under the BOSS design to avoid inducing spatial imbalance. Foster et al. [26] proposed using a squared loss distance metric to alter the inclusion probabilities around “legacy” monitoring sites (i.e., pre-existing sampling locations are known to be a randomly selected representative sample from a known sample frame [26]). This approach would provide a clear way to improve future sampling under the BOSS design without introducing biases and inefficiencies due to spatial imbalances. In addition to data from legacy sites, a large collection of historic data exist within the Boreal Avian Modelling (*hereafter* BAM) project database [75] for a large portion of boreal Canada. The difficulty with incorporating data from the BAM database is that these largely represent “iconic sites” (*sensu* [26]) which are either known to be non-randomly selected and/or there is insufficient documentation to know whether the sampling frame included the full suite of habitat classes. Including iconic sites in a randomized draw could introduce bias into the design and result in biased status and trend estimates that may not match those in the population [26]. Since these historic datasets could potentially add valuable ecological insights into factors affecting populations of boreal birds, it would be fruitful to consider when and how they could be included within a long-term monitoring design.

It is worth nothing that our approach to “optimizing” which randomized draw to use derives from the concept of Pareto optimality [34], but does not strictly meet these criteria. In optimization, a Pareto optimal solution is a solution in which the results cannot be modified without resulting in the objective function becoming worse. In our approach, this could only be accomplished by considering all permutations of a given sample size. Considering all permutations would become computationally difficult as well as result in a solution that is no longer truly random. As such, we feel our approach provides a reasonable approximation of optimality while maintaining the desirable feature of representing a randomized draw.

We have demonstrated that including access costs and habitat within a hierarchically structured sampling scheme was the most cost-efficient design that maintained the desired spatial and habitat representation. Although we have focused on a design for monitoring boreal birds, our approach should be broadly applicable and adaptable as a template for other regions where remote locations form a large proportion of the sampling frame. We recommend that our approach be adapted for use in other large-scale studies such as Breeding Bird Atlases [76] if access and sampling costs are limiting for much if not all of the study areas. For example, the Saskatchewan Breeding Bird Atlas (<https://sk.birdatlas.ca/>) is currently using systematic sampling within accessible areas, and the BOSS design within the boreal portion of the province. To facilitate adaptation and implementation of our approach and/or further refinements to our method, we provide worked examples and scripts within the Supporting Information. Using design-based approaches to inform the design of large-scale studies would add to their statistical rigor and efficiency and facilitate their integration (i.e., treated as legacy sites) into long-term monitoring to provide more responsive conservation efforts in light of widespread declines in biodiversity [1,2,3]. Our hierarchical sampling design should be widely implemented to monitor boreal birds because it can provide an unbiased representation of when and where conservation and management should be targeted [9,77]. Using monitoring data to inform conservation decisions is crucial, given ongoing and projected ecological changes in the boreal biome [18,37] and population declines already observed for boreal birds [3].

# **Acknowledgments**

We are extremely grateful to R. Weeber for discussions leading to several of the concepts incorporated within this design. We thank M. Robertson, D. Iles and D. Hope for helpful comments on an earlier draft of this manuscript. F. Koch, C. Handel, and two anonymous reviewers provided helpful comments that improved the manuscript.

**Author Contributions**

Conceptualization: Van Wilgenburg, Mahon, G. Campbell, McLeod, M. Campbell, Evans, Easton, Francis, Hache, Machtans, Russell, Smith, Thomas, Toms, Tremblay

Data curation: Van Wilgenburg, G. Campbell, McLeod

Formal analysis: Van Wilgenburg, G. Campbell, McLeod

Methodology: Van Wilgenburg, Mahon, G. Campbell, Mader, McLeod

Writing, review and editing: Van Wilgenburg, Mahon, G. Campbell, McLeod, M. Campbell, Evans, Easton, Francis, Hache, Machtans, Mader, Pankratz, Russell, Smith, Thomas, Toms, Tremblay

# **References**

x

| 1. | Pimm SL, Jenkins CN, Abell R, Brooks TM, Gittleman JL, Joppa LN, et al. The biodiversity of species and their rates of extinction, distribution and protection. Science. 2014 May 30; 344(6187): p. 1246752. |
| --- | --- |
| 2. | Butchart SM, Walpole M, Collen B, van Strien A, Scharlemann JP, Almond RE, et al. Global biodiversity: indicators of recent declines. Science. 2010 May 28; 328(5982): p. 1164-1168. |
| 3. | Rosenberg KV, Dokter AM, Blancher PJ, Sauer JR, Smith AC, Smith PA, et al. Decline of the North American avifauna. Science. 2019 September 19; 365(6459): p. eaaw1313. |
| 4. | Sala OE, Chapin III FS, Armesto JJ, Berlow E, Bloomfield J, Dirzo R, et al. Global Biodiversity Scenarios for the Year 2100. Science. 2000 March 10; 287(5459): p. 1771-1774. |
| 5. | Bellard C, Bertelsmeier C, Leadley P, Thuiller W, Courchamp F. Impacts of climate change on the future of biodiversity. Ecology Letters. 2012 April; 15(4): p. 365-377. |
| 6. | Taylor MF, Suckling KF, Rachlinski JJ. The effectiveness of the Endangered Species Act: a quantitative analysis. BioScience. 2005; 4(1): p. 360-367. |
| 7. | Kerr JT, Cihlar J. Patterns and causes of species endangerment in Canada. Ecological Applications. 2004 June 1; 14(3): p. 743-753. |
| 8. | Rice J. Environmental health indicators. Ocean & Coastal Managment. 2003; 46(3-4): p. 235-259. |
| 9. | Pavlacky Jr DC, Lukacs PM, Blakesley JA, Skorkowsky RC, Klute DS, Hahn BA, et al. A statistically rigorous sampling design to integrate avian monitoring and management within Bird Conservation Regions. PLoS ONE. 2017; 12(10): p. e0185924. |
| 10. | Gitzen RA, Millspaugh JJ. Ecological monitoring: the heart of the matter. In Gitzen RA, Millspaugh JJ, Cooper AB, Licht DS, editors. Design and analysis of long-term ecological monitoring studies. New York: Cambridge University Press; 2012. p. 3-22. |
| 11. | Nichols JD, Williams BD. Monitoring for conservation. Trends in Ecology & Evolution. 2006 December; 21(12): p. 668-673. |
| 12. | Hudson MAR, Francis CM, Campbell KJ, Downes CM, Smith AC, Pardieck KL. The role of the North American Breeding Bird Survey in conservation. The Condor. 2017; 119(3): p. 526-545. |
| 13. | Machtans CS, Kardynal KJ, Smith PA. How well do regional or national Breeding Bird Survey data predict songbird population trends at an intact boreal site. Avian Conservation and Ecology. 2014; 9(1): p. 5. |
| 14. | Bart J, Burnham KP, Dunn EH, Francis CM, Ralph CJ. Goals and strategies for estimating trends in landbird abundance. Journal of Wildlife Management. 2004; 68(3): p. 611-626. |
| 15. | Mahon CL, Bayne EM, Sólymos , Matsuoka SM, Carlson M, Dzus E, et al. Does expected future landscape condition support proposed population objectives for boreal birds? Forest Ecology and Management. 2014 January 15; 312. |
| 16. | Mahon CL, Holloway GL, Bayne EM, Toms JD. Additive and interactive cumulative effects on boreal landbirds: winners and losers in a multi-stressor landscape. Ecological Applications. 2019 July; 29(5): p. e01895. |
| 17. | Watson JE, Evans T, Venter O, Williams B, Tulloch A, Stewart C, et al. The exceptional value of intact forest ecosystems. Nature ecology & evolution. 2018 February 26; 2: p. 599–610. |
| 18. | Stralberg D, Bayne EM, Cumming SG, Sólymos P, Song SJ, Schmiegelow FK. Conservation of future boreal forest bird communities considering lags in vegetation response to climate change: a modified refugia approach. Diversity and Distributions. 2015 July 20; 21(9): p. 1112-1128. |
| 19. | SARA (Species at Risk Act). Bill C-5, an Act respecting the protection of wildlife Species at Risk in Canada. 2002.. |
| 20. | Westwood A, Olszybski M, Fox CH, Ford AT, Jacob AL, Moore JW, et al. The role of science in contemporary Canadian environmental decision making: the example of environmental assessment. University of British Columbia Law Review. 2019 February; 52(1): p. 243-292. |
| 21. | COSEWIC (Committe on the Status of Endangered Wildlife in Canada). COSEWIC's assessment process and criteria. 2010. |
| 22. | Van Wilgenburg SL, Beck EM, Obermayer B, Joyce T, Weddle B. Biased representation of disturbance rates in the roadside sampling frame in boreal forests: implications for monitoring design. Avian Conservation and Ecology. 2015 December; 10(2): p. 5. |
| 23. | Tulloch AI, Mustin K, Possingham HP, Szabo JK, Wilson KA. To boldly go where no volunteer has gone before: predicting volunteer activity to prioritize surveys at the landscape scale. Diversity and Distributions. 2013 April 03; 19(4): p. 465-480. |
| 24. | Buckland ST, Marsden SJ, Green RE. Estimating bird abundance: making methods work. Bird Conservation International. 2008; 18(s1): p. S91-S108. |
| 25. | Stevens Jr DL, Olsen AR. Spatially-balanced sampling of natural resources. Journal of the American Statistical Association. 2004; 99(465): p. 262-278. |
| 26. | Foster SD, Hosack GR, Lawrence E, Przeslawski R, Hedge P, Caley MJ, et al. Spatially balanced designs that incorporate legacy sites. Methods in Ecology and Evolution. 2017; 8(11): p. 1433-1442. |
| 27. | Delmelle EM. Spatial sampling. In Fotheringham AS, Rogerson PA, editors. The SAGE handbook of spatial analysis. Los Angeles: SAGE; 2009. p. 183-206. |
| 28. | Delmelle EM, Goovaerts P. Second-phase sampling designs for non-stationary spatial variables. Geoderma. 2009; 153(1-2): p. 205-216. |
| 29. | Neyman J. On the two different aspects of the representative method: the method of stratified sampling and the method of purposive selection. Journal of the Royal Statistical Society. 1934; 97(4): p. 558–625. |
| 30. | Olsen AR, Kincaid TM, Payton Q. Spatially balanced survey designs for natural resources. In Gitzen RA, Millspaugh JJ, Cooper AB, Licht DS, editors. Design and analysis of long-term ecological monitoring studies. New York: Cambridge University Press; 2012. p. 126-150. |
| 31. | Clark RG. Sample design using imperfect design data. Journal of Survey Statistics and Methodology. 2013; 1(1): p. 6-23. |
| 32. | Van Wilgenburg SL, Hobson KA, Kardynal KJ, Beck EM. Temporal changes in avian abundance in aspen-dominated boreal mixedwood forests of central Saskatchewan, Canada. Avian Conservation and Ecology. 2018 June; 13(1): p. 3. |
| 33. | Stehman SV, Overton WS. Environmental sampling and monitoring Patil GP, Rao CR, editors. Amsterdam: Elsevier Science; 1994. |
| 34. | Marler RT, Arora JS. The weighted sum method for multi-objective optimization: new insights. Structural and multidisciplinary optimization. 2010; 41(6): p. 853-862. |
| 35. | Brandt JP. The extent of the North American boreal zone. Environmental Reviews. 2009; 17: p. 101-161. |
| 36. | Synes NW, Brown C, Watts K, White SM, Gilbert MA, Travis JMJ. Emerging opportunities for landscape ecological modelling. Current Landscape Ecology Reports. 2016; 1(4): p. 146-167. |
| 37. | Langham GM, Schuetz JG, Distler T, Soykan CU, Wilsey C. Conservation status of North American birds in the face of future climate change. PLoS ONE. 2015 September; 10(9): p. e0135350. |
| 38. | Thompson WL, White GC, Gowan C. Monitoring vertebrate populations San Diego: Academic Press; 1998. |
| 39. | Government of Canada. Ecological Land Classification, 2017. Ottawa:, Statistics Canada; 2018. Report No.: 978-0-660-24501-0. |
| 40. | Cochran WG. Sampling techniques. 3rd ed. Toronto: John Wiley & Sons; 1977. |
| 41. | GeoInsight Corporation. An approach to assessing risk to terrestrial biodiversity in Canada. State of the Environment Reporting Occasional Paper Series No. 11. Ottawa:, Environment Canada; 1999. |
| 42. | Anderson MJ, Ellingsen KE, McArdle BH. Multivariate dispersion as a measure of beta diversity. Ecology Letters. 2006; 9(6): p. 683-693. |
| 43. | Anderson MJ. Distance‐based tests for homogeneity of multivariate dispersions. Biometrics. 2006; 62(1): p. 245-253. |
| 44. | Cumming SG, Stralberg D, Lefevre KL, Sólymos P, Bayne EM, Fang S, et al. Climate and vegetation hierarchically structure patterns of songbird distribution in the Canadian boreal region. Ecography. 2014 February; 37(2): p. 137-151. |
| 45. | Mahon CL, Holloway G, Sólymos P, Cumming SG, Bayne EM, Schmiegelow F. Community structure and niche characteristics of upland and lowland western boreal birds at multiple spatial scales. Forest Ecology and Management. 2016 February; 361: p. 99-116. |
| 46. | Hobson KA, Bayne E. The effects of stand age on avian communities in aspen-dominated forests of central Saskatchewan, Canada. Forest Ecology and Management. 2000; 136(1-3): p. 121-134. |
| 47. | Parisien MA, Parks SA, Krawchuk MA, Little JM, Flannigan MD, Gowman LM, et al. An analysis of controls on fire activity in boreal Canada: comparing models built with different temporal resolutions. Ecological Applications. 2014; 24(6): p. 1341-1356. |
| 48. | Latifovic R, Homer C, Ressl R, Pouliot D, Hossain S, Colditz R, et al. North American land-change monitoring system. In Giri CP, editor. Remote Sensing of Land Use and Land Cover: Principles and Applications. Boca Raton: CRC/Taylor & Francis; 2012. p. 303-343. |
| 49. | Morissette JL, Kardynal KJ, Bayne EM, Hobson KA. Are boreal riparian bird communities unique? Contrasting riparian and upland bird assemblages in the Boreal Plain of western Canada. Wetlands. 2018 December; 38(6): p. 1299-1311. |
| 50. | Harris I, Jones PD, Osborn TJ, Lister DH. Updated high‐resolution grids of monthly climatic observations – the CRU TS3.10 Dataset. International Journal of Climatology. 2014; 34(3): p. 623-642. |
| 51. | Matsuoka SM, Mahon CL, Handel CM, Sólymos P, Bayne EM, Fontaine PC, et al. Reviving common standards in point-count surveys for broad inference across studies. The Condor: Ornithological Applications. 2014; 116(4): p. 599-608. |
| 52. | Shonfield J, Bayne EM. Autonomous recording units in avian ecological research: current use and future applications. Avian Conservation and Ecology. 2017; 12(1): p. 14. |
| 53. | Kalton G, Anderson DW. Sampling rare populations. Journal of the Royal Statistical Society: Series A. 1986; 149(1): p. 65-82. |
| 54. | Ralph CJ, Geupel GR, Pyle P, Martin TE, DeSante DF. Handbook of field methods for monitoring landbirds. General Technical Report PSW-GTR-144. Albany, California: Pacific Southwest Research Station, USDA Forest Service; 1993. |
| 55. | Oksanen J, Blanchet FG, Kindt R, Legendre P, Minchin PR, O'Hara RB, et al. Vegan: community ecology package. R Package 2.3-3. 2016.. |
| 56. | R Core Team. R: A language and environment for statistical computing. Version 3.3.1. R Foundation for Statistical Computing, Vienna, Austria. 2016.. |
| 57. | Kincaid TM, Olsen AR. spsurvey: spatial survey design and analysis. R package version 4.0.0. 2019. |
| 58. | Zeileis A, Hothorn T. Diagnostic checking in regression relationships. R News. 2002; 2(3): p. 7-10. |
| 59. | Zeileis A. Object-oriented computation of sandwich estimators. Journal of Statistical Software. 2006; 16: p. 1-16. |
| 60. | Freeman SN, Nobble DG, Newson SE, Baillie SR. Modelling population changes using data from different surveys: the Common Birds Census and the Breeding Bird Survey. Bird Study. 2007; 54(1): p. 61-72. |
| 61. | Fancy SG, Gross JE, Carter SL. Monitoring the condition of natural resources in US national parks. Environmental Monitoring and Assessment. 2009; 151(1-4): p. 161-174. |
| 62. | Smith AN, Anderson MJ, Pawley MD. Could ecologists be more random? Straightforward alternatives to haphazard spatial sampling. Ecography. 2017 November; 40: p. 1251-1255. |
| 63. | Siegel RB, Wilkerson RL, The Institute for Bird Populations , Jenkins KJ, U.S. Geological Survey , Kuntz II RC, et al. Landbird monitoring protocol for National Parks in the North Coast and Cascades Network. Techniques and Methods 2-A6. U.S. Geological Survey, U.S. Department of the Interior; 2007. |
| 64. | Weber S, Woodward A, Freilich J. North Coast and Cascades Network Vital Signs Monitoring Report. Natural Resource Report NPS/NCCN/NRR—2009/098. Fort Collins: National Park Service, U.S. Department of the Interior; 2005. |
| 65. | Metzger MJ, Brus DJ, Bunce RGH, Carey PD, Gonçalves J, Honrado JP, et al. Environmental stratifications as the basis for national, European and global ecological monitoring. Ecological Indicators. 2013 October; 33: p. 26-35. |
| 66. | Roy C, Michel NL, Handel CM, Van Wilgenburg SL, Burkhalter JC, Gurney KEB, et al. Monitoring boreal avian populations: how can we estimate trends and trajectories from noisy data? Avian Conservation and Ecology. 2019; 14(2): p. 8. |
| 67. | Nahorniak M, Larsen DP, Volk C, Jordan CE. Using inverse probability bootstrap sampling to eliminate sample induced bias in model based analysis of unequal probability samples. PLoS ONE. ; 10(6): p. e0131765. |
| 68. | van Turnhout CAM, Willems F, Plate C, van Strien A, Teunissen W, van Dijk A, et al. Monitoring common and scarce breeding birds in the Netherlands: applying a post-hoc stratification and weighting procedure to obtain less biased population trends. Revista Catalana d'Ornitologia. 2008; 24: p. 15-29. |
| 69. | Lumley T. Analysis of complex survey samples. Journal of statistical software. 2004; 9(1): p. 1-19. |
| 70. | Pfeffermann D, Skinner CJ, Holmes DJ, Goldstein H, Rasbash J. Weighting for unequal selection probabilities in multilevel models. Journal of the Royal Statistical Society: series B (statistical methodology). 1998; 60(1): p. 23-40. |
| 71. | Little RJ. Estimating a finite population mean from unequal probability samples. Journal of the American Statistical Association. 1983; 78(383): p. 596-604. |
| 72. | Sólymos P, Toms JD, Matsuoka SM, Cumming SG, Barker NKS, Thogmartin WE, et al. Lessons learned from comparing spatially explicit models and the Partners in Flight approach to estimate population sizes of boreal birds in Alberta, Canada. Condor. 2020 XX; XX(XX). |
| 73. | Van Wilgenburg SL, Sólymos P, Kardynal KJ, Frey MD. Paired sampling standardizes point count data from humans and acoustic recorders. Avian Conservation and Ecology. 2017; 12(1): p. 13. |
| 74. | Vold ST, Handel CM, McNew LB. Comparison of acoustic recorders and field observers for monitoring tundra bird communities. Wildlife Scoiety Bulletin. 2017; 41(3): p. 566-576. |
| 75. | Barker NK, Fontaine PC, Cumming SG, Stralberg D, Westwood A, Bayne EM, et al. Ecological monitoring through harmonizing existing data: Lessons from the boreal avian modelling project. Wildlife Society Bulletin. 2015; 39(3): p. 480-487. |
| 76. | McCabe JD, Anich NM, Brady RS, Zuckerberg B. Raising the bar for the next generation of biological atlases: using existing data to inform the design and implementation of atlas monitoring. Ibis. 2018 July; 160(3): p. 528-541. |
| 77. | Wilson KA, Underwood EC, Morrison SA, Klausmeyer KR, Murdoch WW, Reyers B, et al. Conserving biodiversity efficiently: what to do, where, and when. PLOS Biology. 2007 August 21; 5(9): p. e223. |

x

Table 1. Linear model results comparing variation in access costs ($) between alternative monitoring designs. The cost design was the reference category (Intercept).

| Design | β | SE | t-value |
| --- | --- | --- | --- |
| *Newfoundland* |  |  |  |
| Intercept | 1,006,006.0 | 2870.2 | 350.51 |
| BOSS design | 121,535.7 | 3951.3 | 30.76 |
| Spatial design | 362,012.3 | 4072.3 | 88.90 |
| Habitat design | 482,229.8 | 4023.7 | 119.85 |
|  |  |  |  |
| *Saskatchewan* |  |  |  |
| Intercept | 1,053,370.5 | 4927.6 | 213.77 |
| BOSS design | 175,634.7 | 7288.8 | 24.10 |
| Spatial design | 561,151.8 | 7587.2 | 73.96 |
| Habitat design | 1,020,042.6 | 8439.4 | 120.87 |
|  |  |  |  |
| *Yukon* |  |  |  |
| Intercept | 331,627.1 | 990.8 | 334.70 |
| BOSS design | 8700.2 | 1615.1 | 5.39 |
| Spatial design | 541,671.3 | 1658.7 | 326.57 |
| Habitat design | 121,812.7 | 1507.5 | 80.80 |

Table 2. Linear model results comparing variation in habitat representation (i.e., sum of squared differences between area of land cover classes represented within sample draws against a balanced sample (see Methods)) between alternative sampling designs. The habitat design was the reference category (Intercept).

| Design | β | SE | t-value |
| --- | --- | --- | --- |
| *Newfoundland* |  |  |  |
| Intercept | 0.06 | 3.84 x 10^-4^ | 158.59 |
| BOSS design | 4.90 x 10^-3^ | 5.60 x 10^-4^ | 8.75 |
| Spatial design | 0.11 | 9.97 x 10^-4^ | 108.35 |
| Cost design | 0.10 | 8.97 x 10^-4^ | 114.37 |
|  |  |  |  |
| *Saskatchewan* |  |  |  |
| Intercept | 0.04 | 2.06 x 10^-4^ | 215.01 |
| BOSS design | 9.36 x 10^-4^ | 3.10 x 10^-4^ | 3.02 |
| Spatial design | 0.01 | 4.02 x 10^-4^ | 32.31 |
| Cost design | 0.02 | 3.93 x 10^-4^ | 41.56 |
|  |  |  |  |
| *Yukon* |  |  |  |
| Intercept | 0.10 | 4.78 x 10^-4^ | 211.13 |
| BOSS design | 1.53 x 10^-3^ | 7.17 x 10^-4^ | 2.14 |
| Spatial design | 0.04 | 6.47 x 10^-4^ | 61.51 |
| Cost design | 0.04 | 8.40 x 10^-4^ | 53.36 |

Table 3. Linear model results examining variation in spatial balance (Pielou evenness) between alternative sampling designs. The equal probability spatially balanced sampling design was the reference category (Intercept).

| Design | β | SE | t-value |
| --- | --- | --- | --- |
| *Newfoundland* |  |  |  |
| Intercept | 0.99 | 6.52 x 10^-4^ | 1514.78 |
| BOSS design | 8.70 x 10^-3^ | 8.80 x 10^-4^ | 9.87 |
| Cost design | 1.56 x 10^-3^ | 9.03 x 10^-4^ | 1.72 |
| Habitat design | 4.18 x 10^-3^ | 8.92 x 10^-4^ | 4.68 |
|  |  |  |  |
| *Saskatchewan* |  |  |  |
| Intercept | 0.99 | 3.32 x 10^-4^ | 2977.07 |
| BOSS design | -1.10 x 10^-3^ | 4.23 x 10^-4^ | -2.61 |
| Cost design | 9.22 x 10^-4^ | 4.52 x 10^-4^ | 2.04 |
| Habitat design | -1.39 x 10^-2^ | 4.41 x 10^-4^ | -31.61 |
|  |  |  |  |
| *Yukon* |  |  |  |
| Intercept | 1.00 | 3.12 x 10^-4^ | 3204.27 |
| BOSS design | -1.59 x 10^-2^ | 5.81 x 10^-4^ | 27.41 |
| Cost design | -6.15 x 10^-3^ | 5.69 x 10^-4^ | -10.82 |
| Habitat design | -1.21 x 10^-2^ | 5.89 x 10^-4^ | -20.68 |

**Figure Headings**

Fig 1. Spatial extent of the sampling frame of the Boreal Optimal Sampling Strategy (BOSS) design. The green region represents the boundary of the boreal region as defined in [35] with a 100 km northern buffer within the 4-Northwestern Interior Forest, 6-Boreal Taiga Plains, 7-Taiga Shield and Hudson Plains, and 8-Boreal Softwood Shield Bird Conservation Regions. Reprinted from [35] under a CC BY license, with permission from NRC Research Press, original copyright 2009.

Fig 2. The two-stage sampling design of the Boreal Optimal Sampling Strategy (BOSS) design. This figure illustrates the selection of Primary Sampling Units (PSU, 5 km diameter hexagonal grid cell) from ecoregions within the sampling frame and selection of Secondary Sampling Units (SSU, point count survey locations) within the selected PSUs. Reprinted from [35] under a CC BY license, with permission from NRC Research Press, original copyright 2009.

Fig 3. Components of the Boreal Optimal Sampling Strategy (BOSS) design depicted for Newfoundland and Labrador, Saskatchewan, and Yukon Territory: (a) access cost; (b) weighted habitat inclusion probability; (c) cost and weighted habitat inclusion probability. Reprinted from [35] under a CC BY license, with permission from NRC Research Press, original copyright 2009.

Fig 4. Allocation of sampling effort between boreal ecoregions of Canada under the Boreal Optimal Sampling Strategy (BOSS; see Methods) versus traditional allocation based on Stratum areas to achieve a total sample size of 4980 Primary Sample Units (i.e. 2% sample). Dashed line indicates the 1:1 correspondence line.

Fig 5. Geographic distribution of sampling intensity (number of allocated primary sampling units per 1000 km^2^) across the boreal ecoregions of Canada under the Boreal Optimal Sampling Strategy (BOSS) design (see Methods). Reprinted from [35] under a CC BY license, with permission from NRC Research Press, original copyright 2009.

Fig 6. Variation in predicted costs (in Canadian dollars x 10^6^) of accessing 400 primary sampling units in each of Newfoundland and Labrador, Saskatchewan and Yukon Territory. Data are from 100 separate random draws of 400 primary sampling units in each jurisdiction. Note differing y-axis scales due to variation in overall costs of operating in each jurisdiction. Dark solid line represents the median, box indicates the inter-quartile range, whiskers are 1.5 time the inter-quartile range, and dots indicate extreme values.

Fig 7. Variation in habitat class representation (measured as the sum of square differences between the area of each habitat class within all PSUs in a randomized draw versus the proportional area of each habitat class, see Materials and Methods) between alternative sampling designs in Newfoundland and Labrador, Saskatchewan and Yukon Territory. Data are from 100 separate random draws of 400 primary sampling units in each jurisdiction. Dark solid line represents the median, box indicates the inter-quartile range, whiskers are 1.5 time the inter-quartile range, and dots indicate extreme values.

Fig 8. Variation in spatial balance (Pielou evenness) between alternative sampling designs in Newfoundland, Saskatchewan and Yukon Territory. Data are from 100 separate random draws of 400 primary sampling units in each jurisdiction. Dark solid line represents the median, box indicates the inter-quartile range, whiskers are 1.5 time the inter-quartile range, and dots indicate extreme values.

Fig 9. Between draw variation in combined spatial and habitat (weighted sum, see Methods) representativeness of sampling relative to access costs in Newfoundland and Labrador, Saskatchewan and Yukon Territory for four alternative sampling designs: cost based sampling design (open red triangles), the BOSS design (open black squares), the habitat design (open green triangles), and an equal probability spatial sampling (blue filled triangles). Red filled square within the cluster of points representing the BOSS design represents the lowest cost solution that maximizes combined spatial and habitat representation. Note differing x-axis scales due to variation in overall costs of operating in each jurisdiction.

Fig 10. Predicted versus realized access costs (in Canadian dollars) for each of Newfoundland and Labrador, Saskatchewan and Yukon Territory. Solid line represents ordinary least squares fit and dashed lines represent 95% confidence intervals. NOTE: points represent average costs to access multiple (n = 1–17) primary sampling units in line with how charter services were billed.

**Supporting Information**

S1: Data used as proxy variables for assumed spatial and temporal variation in boreal forest bird communities.

S2: R script implementing primary and secondary sample unit selection, including example calculation of access costs for Northwest Territories, Canada.

S3: Allocation of 4980 primary sampling units amongst geographic strata (intersection between ecoregions and jurisdictions) across Canada.

S4: zip file containing landcover data for habitat stratification derived from Latifovic et al. [48] used in association with R scripts in S2.

S5: Shapefiles for required to run example analyses in script S2.

S6: R script implementing parallel computing for primary and secondary sample unit selection for Manitoba, Canada, where access cost models were created using ArcGIS using the Spatial Analyst™ extension.

S7: zip file containing example geospatial data, metadata describing of cost modeling, and supporting R scripts used in association with R scripts in S6.

S8. Comma separated values file of data from 100 iterations of drawing 400 primary sample units for three Canadian jurisdictions (Newfoundland & Labrador, Saskatchewan and Yukon Territory).
