# Supplementary material for: A cost efficient spatially balanced hierarchical sampling design for monitoring boreal birds incorporating access costs and habitat stratification
Source: PLoS One. 2020 Jun 16;15(6):e0234494. doi: 10.1371/journal.pone.0234494 (PMC7297386; doi:10.1371/journal.pone.0234494)
Supplement: Attachment — Submitted filename: Response to the reviewers.docx [file pone.0234494.s012.docx]

Frank H. Koch, PhD

Academic Editor

PLOS ONE

Re: Manuscript # PONE-D-19-35579R1

Dear Dr. Koch,

I had originally not included a response to the reviewers letter as your previous edits were solely in the manuscript copy in track changes. This still appears in the submitted revision. The editorial management system required this letter, so include for completeness. As such, there are no direct responses included here. I simply accepted all your changes.

I have included your original review below for reference.

We hope the manuscript is now suitable for publication.

Regards

-Steven Van Wilgenburg

PONE-D-19-35579R1
A cost efficient spatially balanced hierarchical sampling design for monitoring boreal birds incorporating access costs and habitat stratification
PLOS ONE

Dear Mr Van Wilgenburg,

Thank you for submitting your manuscript to PLOS ONE. After careful consideration, we feel that it has merit but does not fully meet PLOS ONE’s publication criteria as it currently stands. Therefore, we invite you to submit a revised version of the manuscript that addresses the points raised during the review process.

I appreciate the time and care you put into your responses to the reviewers. I believe you have addressed their concerns fully, and the manuscript is nearly suitable for publication. In reading through revision 1, I noticed some minor editorial errors (omitted words, punctuation, or the like). Rather than list them here, I've attached a tracked changes version of the manuscript for your reference. Once you address these and resubmit, I will move quickly to accept your manuscript. You do not need to submit point-by-point responses to any of my edits or comments.

We would appreciate receiving your revised manuscript by Jun 13 2020 11:59PM. When you are ready to submit your revision, log on to <https://www.editorialmanager.com/pone/> and select the 'Submissions Needing Revision' folder to locate your manuscript file.

If you would like to make changes to your financial disclosure, please include your updated statement in your cover letter.

To enhance the reproducibility of your results, we recommend that if applicable you deposit your laboratory protocols in protocols.io, where a protocol can be assigned its own identifier (DOI) such that it can be cited independently in the future. For instructions see: <http://journals.plos.org/plosone/s/submission-guidelines#loc-laboratory-protocols>

Please include the following items when submitting your revised manuscript:

- A marked-up copy of your manuscript that highlights changes made to the original (R1) version. This file should be uploaded as separate file and labeled 'Revised Manuscript with Track Changes'.
- An unmarked version of your revised paper without tracked changes. This file should be uploaded as separate file and labeled 'Manuscript'.

Please note while forming your response, if your article is accepted, you may have the opportunity to make the peer review history publicly available. The record will include editor decision letters (with reviews) and your responses to reviewer comments. If eligible, we will contact you to opt in or out.

We look forward to receiving your revised manuscript.

Kind regards,

Frank H. Koch, PhD
Academic Editor
PLOS ONE
